# Supplementary material for: Impact of the COVID-19 pandemic on remote mental healthcare and prescribing in psychiatry: an electronic health record study
Source: BMJ Open. 2021 Mar 30;11(3):e046365. doi: 10.1136/bmjopen-2020-046365 (PMC8728386; doi:10.1136/bmjopen-2020-046365)
Supplement: Supplementary data [file bmjopen-2020-046365supp002.pdf]

Impact of the COVID-19 pandemic on remote mental healthcare and prescribing in psychiatry: an electronic health record study

Supplementary Material

Table of Contents

Interpretation of the interactive dashboard 1

Supplementary Figure 1. Number of patients registered with SLaM services each week by gender 2

Supplementary Figure 2. Number of patients newly registered with SLaM services each week........ 3

Supplementary Figure 3. Number of remote consultations by video call vs telephone each month 3

Supplementary Figure 4. Remote consultations by video-call vs telephone as percentages of the total 4

Supplementary Figure 5. Total number of clinical events each week by gender........ 4

Supplementary Figure 6. Number of in-person, remote and non-attended clinical events each week by gender 5

Supplementary Figure 7. Number of antipsychotics recorded each week 5

Supplementary Figure 8. Number of antipsychotics recorded per week in structured fields vs free text 6

Supplementary Figure 9. Number of antipsychotics recorded each week by age group 6

Supplementary Figure 10. Number of antipsychotics recorded each week by gender 7

Supplementary Figure 11: Number of mood stabilisers recorded each week........ 7

Supplementary Figure 12. Number of mood stabilisers recorded each week by gender........ 8

Supplementary Figure 13. Number of mood stabilisers recorded each week by age group 8

Interpretation of the interactive dashboard

The data visualisation dashboard at http://rpatel.co.uk/TelepsychiatryDashboard has a number of interactive capabilities:

1. Click and drag along the axes to scroll to different values
2. To zoom in on a time period, click and drag horizontally in the chart space to highlight the time period of interest
3. To zoom in on a data series, click and drag vertically in the chart space to highlight the data series of interest
4. Double click to exit zoom mode and revert to default
5. Click on a legend item to hide that category of data from the chart temporarily

Patel R, *et al. BMJ Open* 2021; 11:e046365. doi: 10.1136/bmjopen-2020-046365

More information can be found at https://exploratory.io/note/2ac8ae888097/0937480844541865.

Supplementary Figure 1. Number of patients registered with SLaM services each week by gender

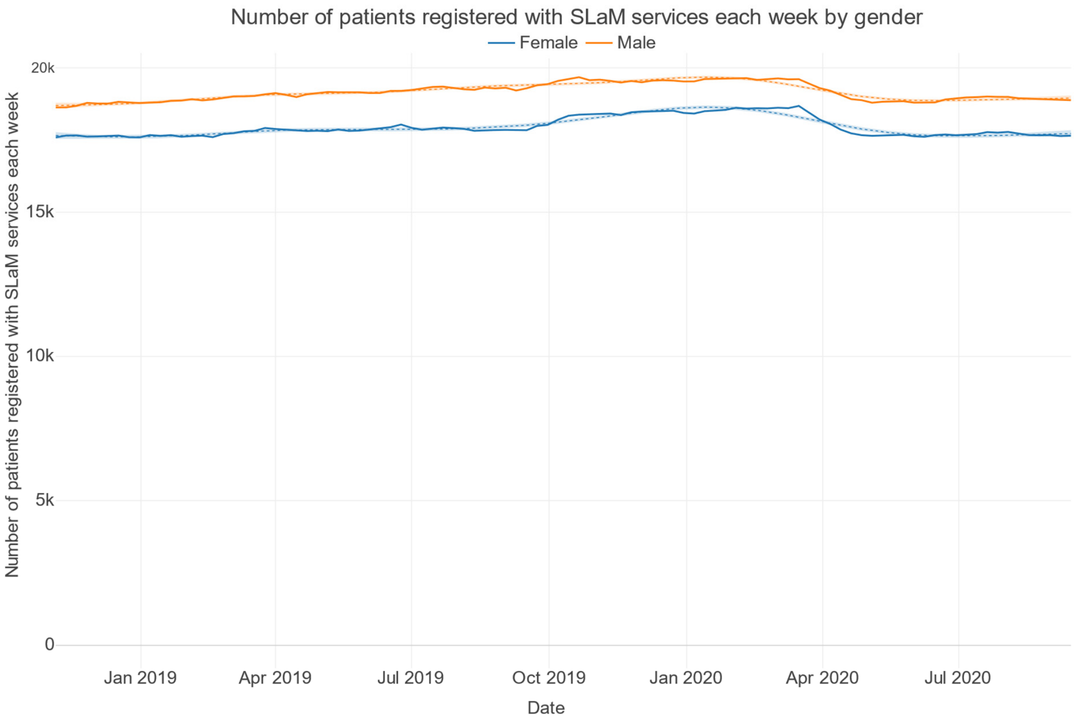

Patel R, *et al. BMJ Open* 2021; 11:e046365. doi: 10.1136/bmjopen-2020-046365

Supplementary Figure 2. Number of patients newly registered with SLaM services each week

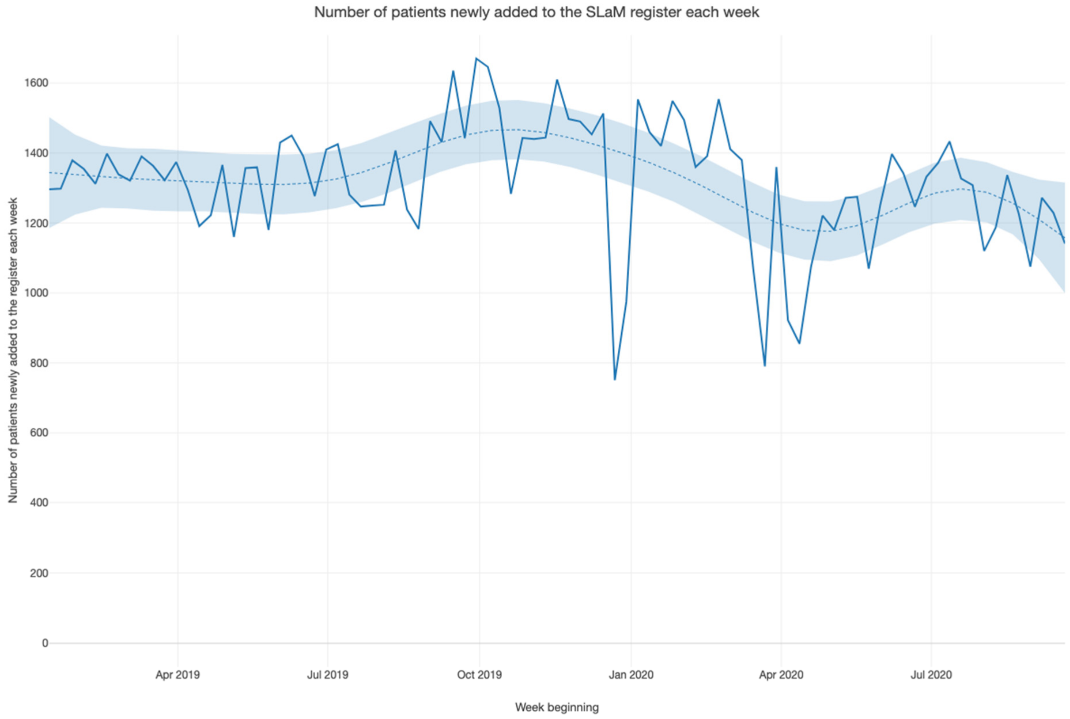

Supplementary Figure 3. Number of remote consultations by video call vs telephone each month

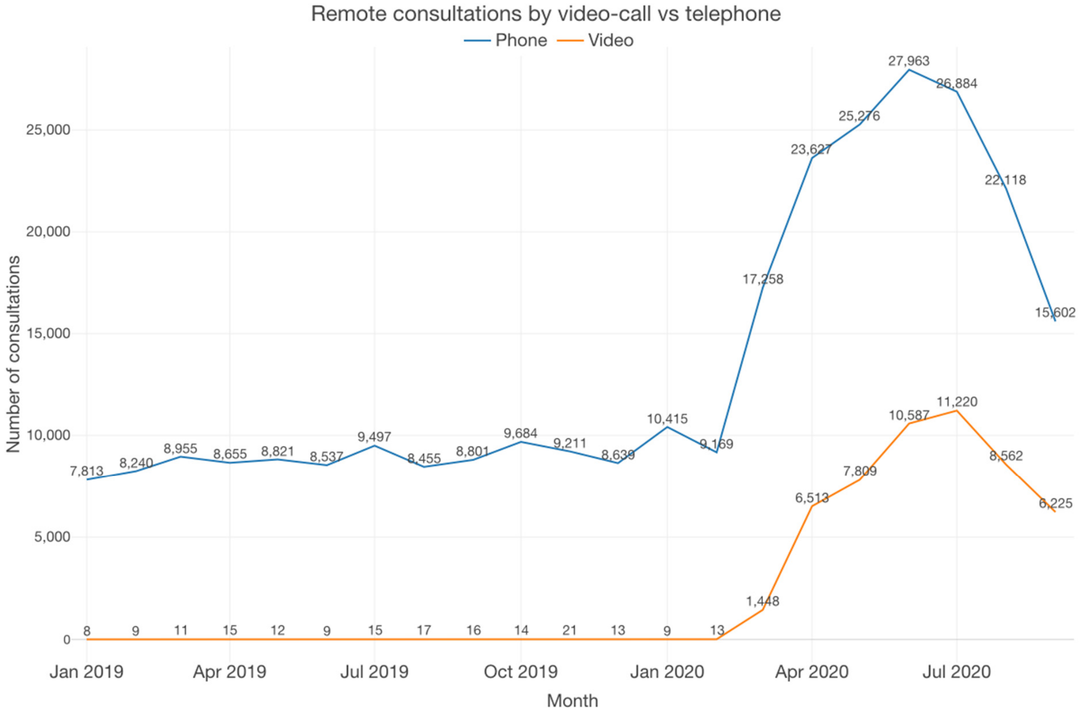

Patel R, *et al. BMJ Open* 2021; 11:e046365. doi: 10.1136/bmjopen-2020-046365

Supplementary Figure 4. Percentage of remote consultations by video-call vs telephone

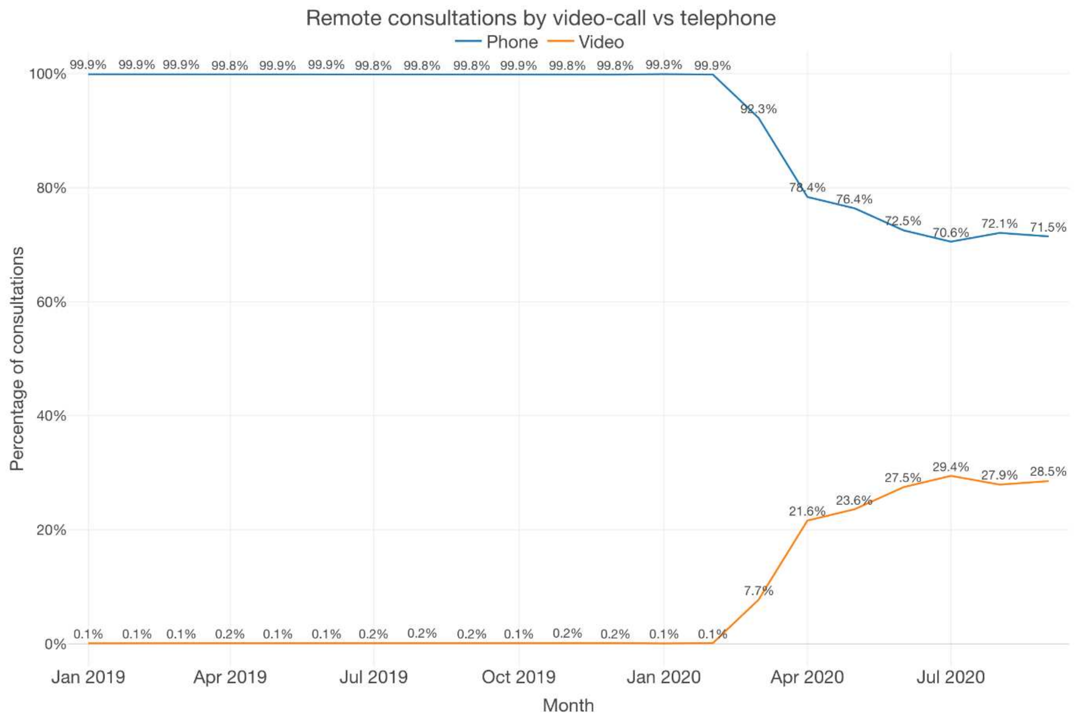

Supplementary Figure 5. Total number of clinical events each week by gender

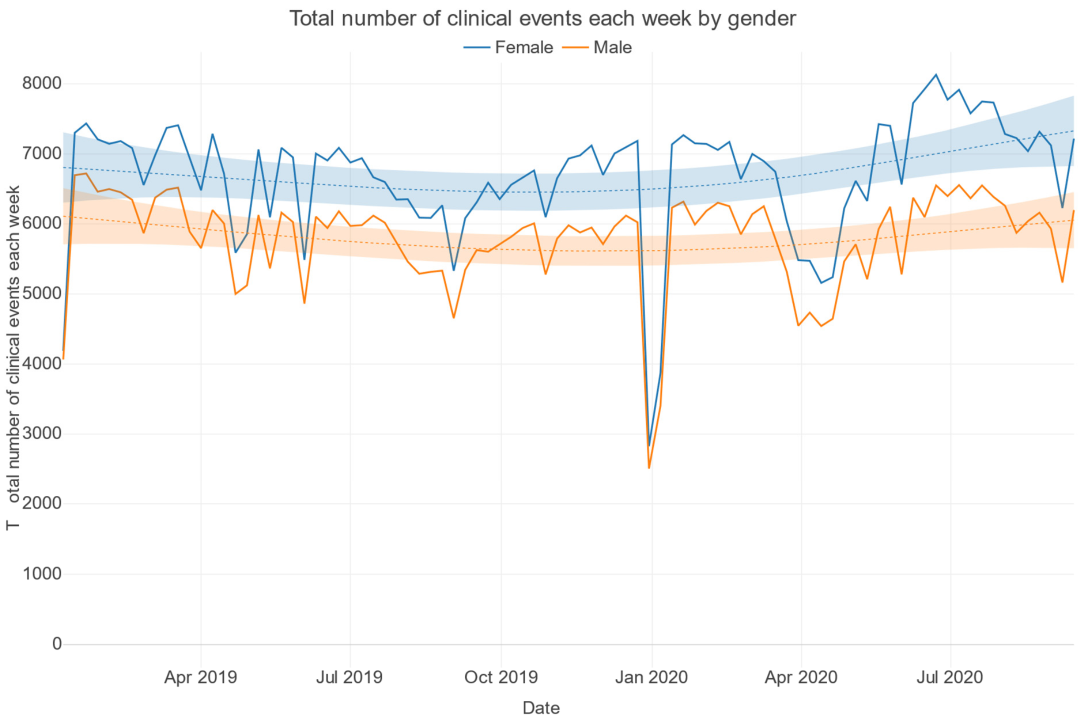

Patel R, *et al. BMJ Open* 2021; 11:e046365. doi: 10.1136/bmjopen-2020-046365

Supplementary Figure 6. Number of in-person, remote and non-attended clinical events each week by gender

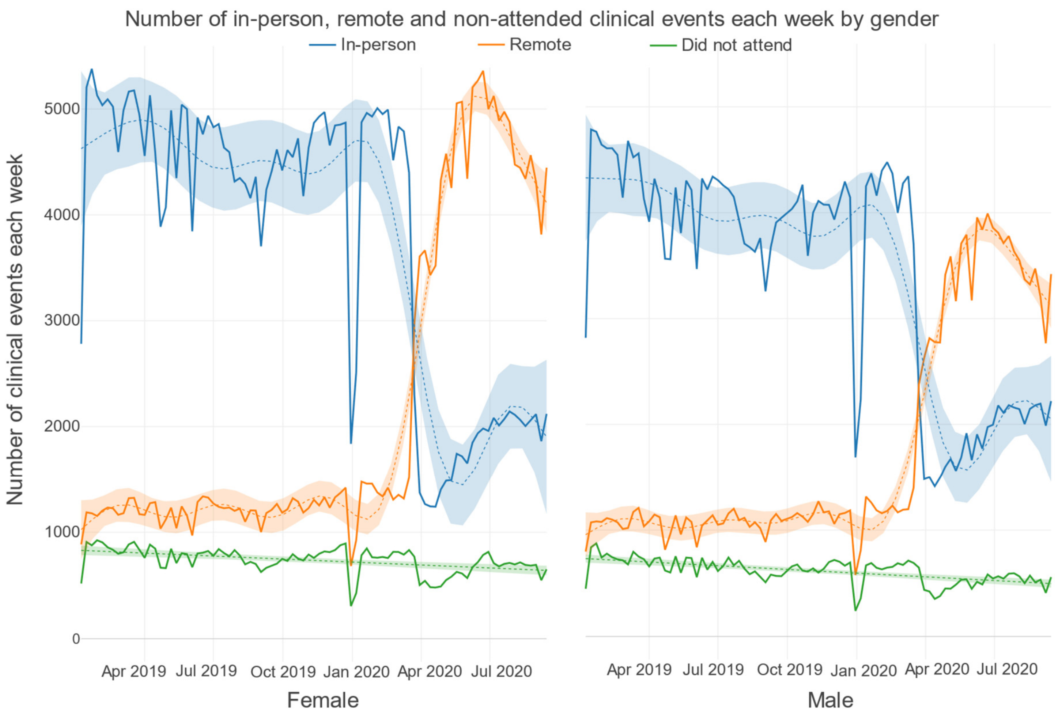

Supplementary Figure 7. Number of antipsychotics recorded each week

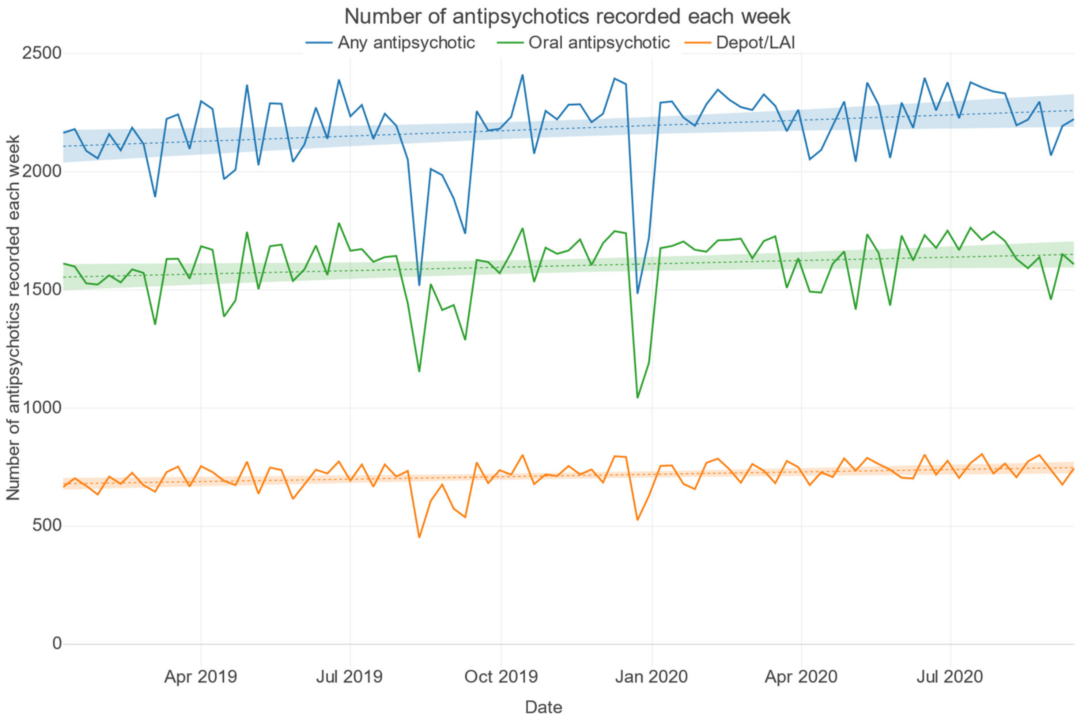

Patel R, *et al. BMJ Open* 2021; 11:e046365. doi: 10.1136/bmjopen-2020-046365

Supplementary Figure 8. Number of antipsychotics recorded per week in structured fields vs free text

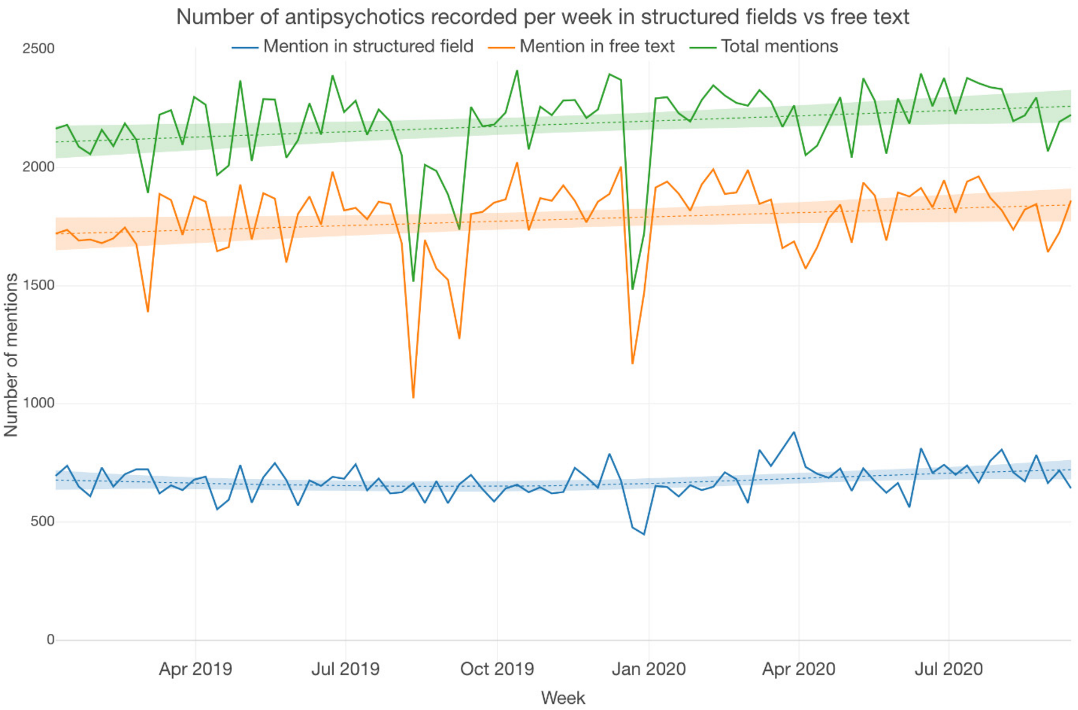

Supplementary Figure 9. Number of antipsychotics recorded each week by age group

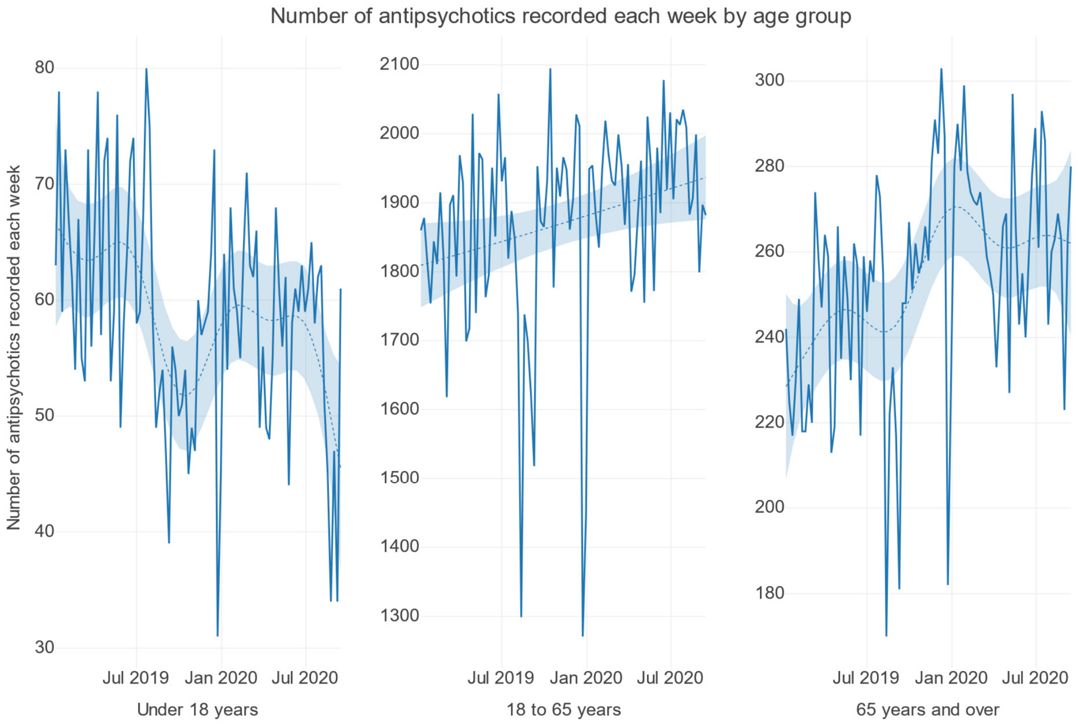

Note: The y-axes presented here do not all start at zero and are not standardised between charts.

Supplementary Figure 10. Number of antipsychotics recorded each week by gender

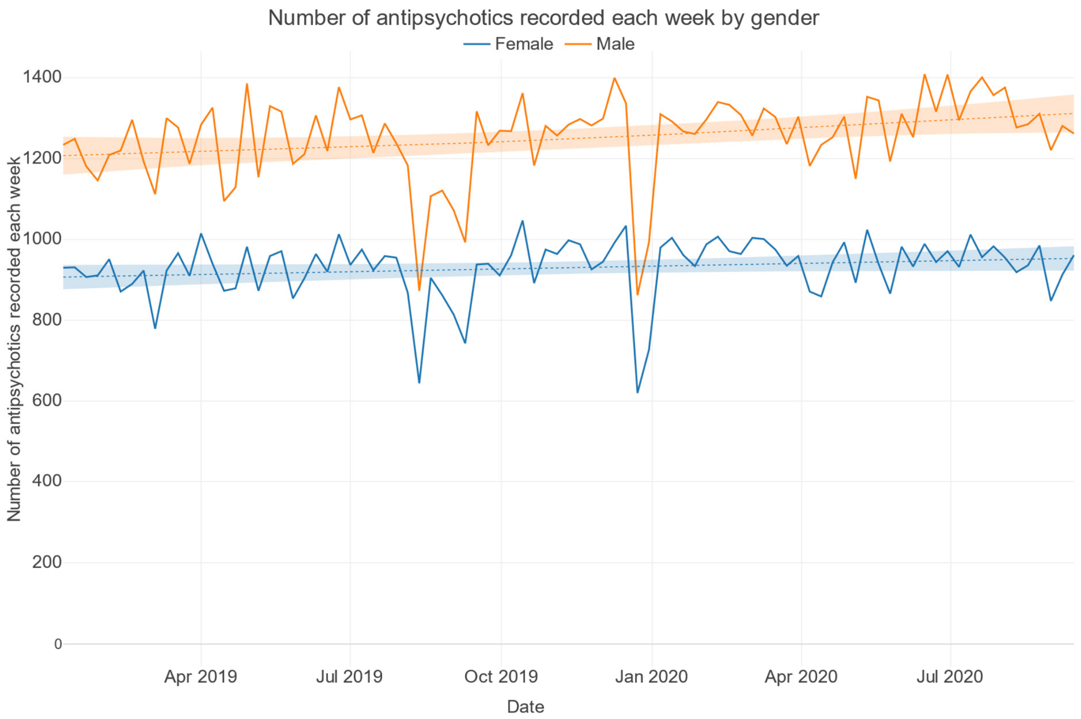

Supplementary Figure 11: Number of mood stabilisers recorded each week

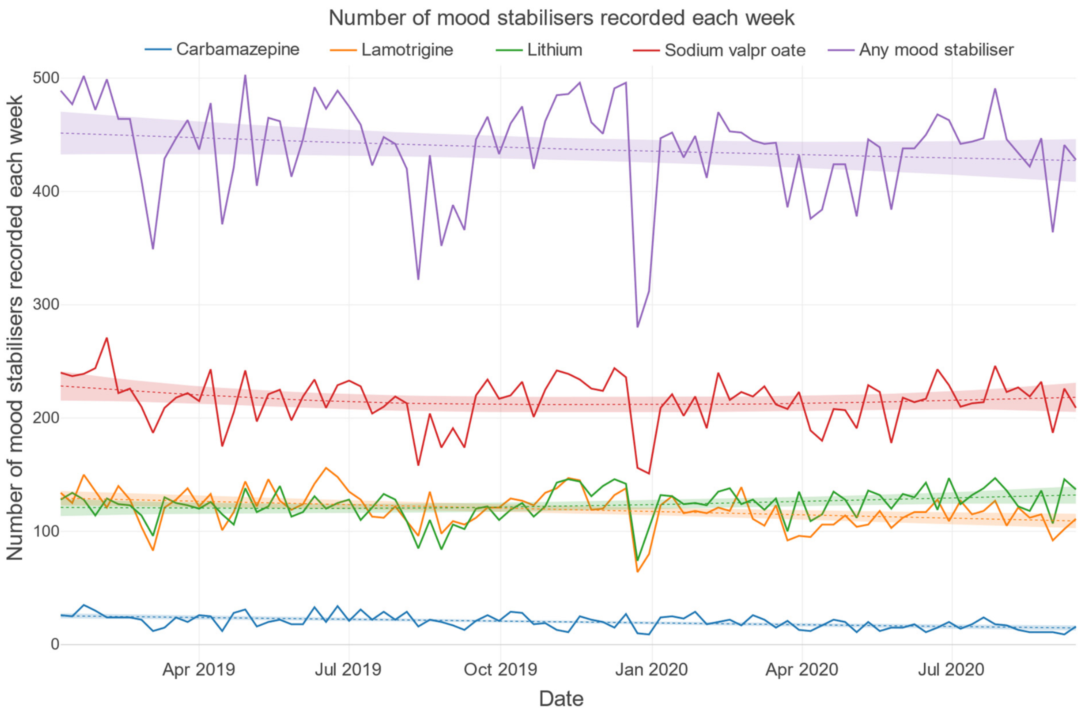

Patel R, *et al. BMJ Open* 2021; 11:e046365. doi: 10.1136/bmjopen-2020-046365

Supplementary Figure 12. Number of mood stabilisers recorded each week by gender

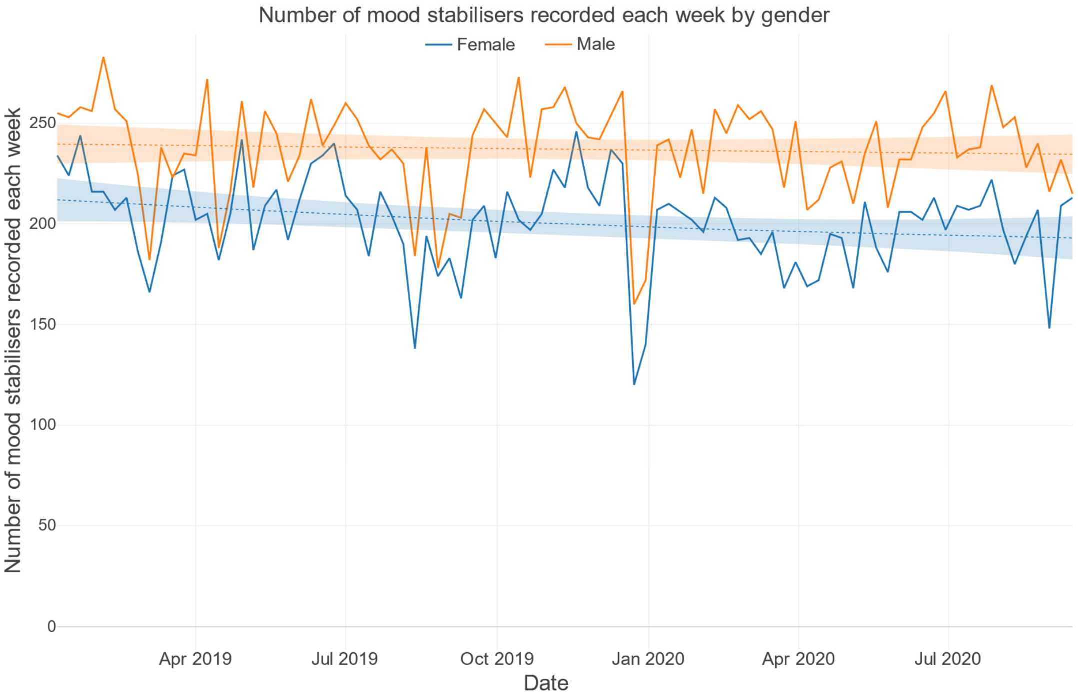

Supplementary Figure 13. Number of mood stabilisers recorded each week by age group

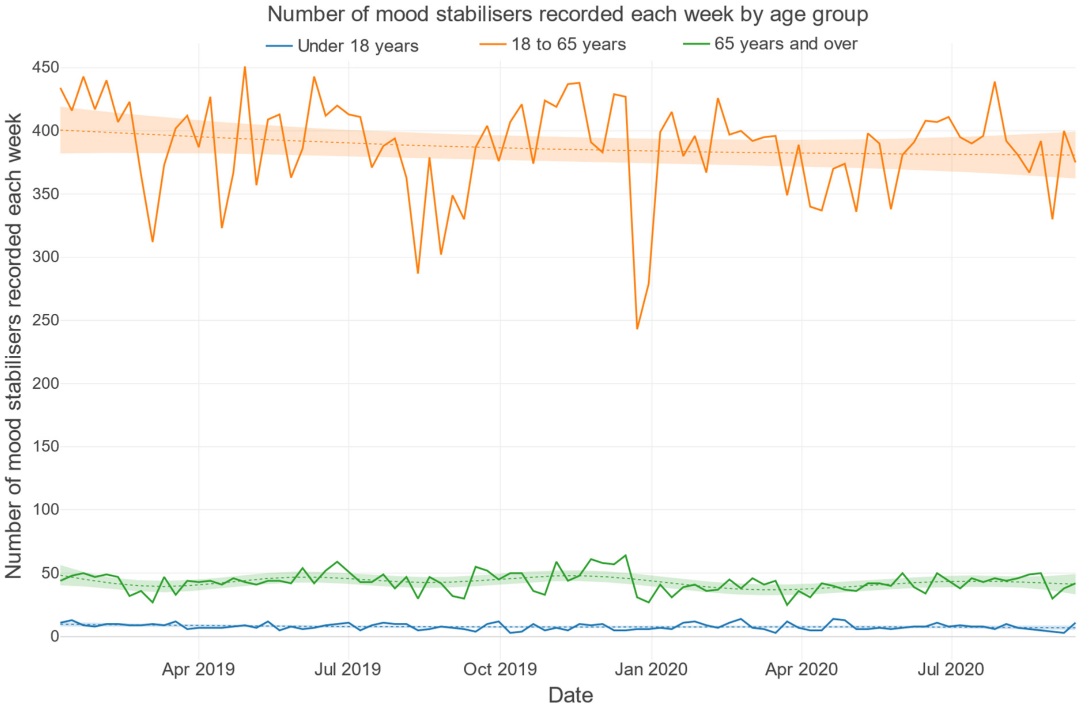

Patel R, *et al. BMJ Open* 2021; 11:e046365. doi: 10.1136/bmjopen-2020-046365
