# Supplementary material for: Impact of the COVID-19 pandemic on remote mental healthcare and prescribing in psychiatry: an electronic health record study
Source: BMJ Open. 2021 Mar 30;11(3):e046365. doi: 10.1136/bmjopen-2020-046365 (PMC8728386; doi:10.1136/bmjopen-2020-046365)
Supplement: Supplementary data [file bmjopen-2020-046365supp001.pdf]

Supplementary Table 1

Variable	Index period: 4 March 2019 to 1 March 2020	Index period vs March 2020				April 2020				May 2020			
Number of patients enrolled in mental health services	**Mean (95% Confidence Interval)**	**β coefficient: mean number of patients**	**p**	**95% CI Lower limit**	**95% CI Upper limit**	**β coefficient: number of patients**	**p**	**95% CI Lower limit**	**95% CI Upper limit**	**β coefficient: number of patients**	**p**	**95% CI Lower limit**	**95% CI Upper limit**
**Total**	37563.2 (37419.8 to 37706.6)	711.5	0.009	200.6	1222.5	-333.2	0.211	-849.6	183.2	-918.2	<0.001	-1373.5	-462.9
**Female**	18111.4 (18026.3 to 18196.4)	470.1	0.004	167.3	773.0	-149.9	0.341	-455.8	156.1	-453.2	0.002	-723.1	-183.3
**Male**	19355.6 (19296.5 to 19414.7)	221.9	0.044	11.0	432.8	-226.8	0.042	-440.5	-13.2	-515.6	<0.001	-703.5	-327.7
**Under 18 years**	8648.3 (8546.1 to 8750.4)	877.7	<0.001	514.7	1240.7	723.2	<0.001	360.2	1086.3	570.7	0.001	246.4	895.1
**18 to 64 years**	25810.7 (24945.4 to 25010.6)	-151.5	0.140	-349.7	46.8	-738.0	<0.001	-940.0	-535.9	-1076.9	<0.001	-1252.3	-901.6
**65 years and older**	3041.4 (3029.8 to 3053.0)	-21.2	0.365	-66.6	24.2	-349.4	<0.001	-396.2	-302.6	-456.6	<0.001	-493.5	-419.7
Number of contacts with mental health professionals	**Mean (95% Confidence Interval)**	**β coefficient: mean number of weekly contacts**	**p**	**Lower limit**	**Upper limit**	**β coefficient: number of weekly contacts**	**p**	**Lower limit**	**Upper limit**	**β coefficient: number of weekly contacts**	**p**	**Lower limit**	**Upper limit**
**In person**	8581.8 (8325.7 to 8838.0)	-847.6	0.174	-2052.7	357.6	-5829.6	<0.001	-6919.5	-4739.6	-5335.8	<0.001	-6312.5	-4359.1
**Remote**	2328.2 (2267.3 to 2389.2)	948.3	<0.001	546.5	1350.1	3338.5	<0.001	3074.4	3602.7	4773.4	<0.001	4501.0	5045.8
**Did not attend (DNA)**	1411.9 (1366.8 to 1456.9)	66.4	0.501	-125.7	258.5	-525.4	<0.001	-717.5	-333.2	-359.9	<0.001	-533.0	-186.8
Number of antipsychotic mentions	**Mean (95% Confidence Interval)**	**β coefficient: mean number of weekly antipsychotics**	**p**	**Lower limit**	**Upper limit**	**β coefficient: number of weekly antipsychotics**	**p**	**Lower limit**	**Upper limit**	**β coefficient: number of weekly antipsychotics**	**p**	**Lower limit**	**Upper limit**
**Any antipsychotic**	2056.0 (2020.1 to 2091.3)	89.8	0.256	-63.4	242.9	10.3	0.897	-144.3	164.9	42.2	0.559	-98.5	182.9
**Any depot**	666.9 (655.0 to 678.7)	30.6	0.252	-21.2	82.5	20.1	0.443	-30.9	71.2	58.1	0.015	12.5	103.7
**Any oral**	1495.2 (1465.7 to 1524.6)	55.3	0.398	-72.0	182.7	-13.9	0.830	-140.6	112.8	-15.4	0.797	-131.8	101.1
Number of individual antipsychotic mentions	**Mean (95% Confidence Interval)**	**β coefficient: mean number of weekly antipsychotics**	**p**	**Lower limit**	**Upper limit**	**β coefficient: number of weekly antipsychotics**	**p**	**Lower limit**	**Upper limit**	**β coefficient: number of weekly antipsychotics**	**p**	**Lower limit**	**Upper limit**
**Amisulpiride**	90.3 (87.4 to 93.2)	1.2	0.855	-11.8	14.2	-1.5	0.813	-14.2	11.2	-7.9	0.179	-19.2	3.5
**Aripiprazole**	336.6 (327.3 to 346.0)	34.1	0.102	-6.0	74.3	-27.9	0.180	-68.1	12.4	-3.4	0.854	-39.6	32.7
**Clozapine**	348.3 (340.0 to 356.7)	14.7	0.445	-22.7	52.0	79.2	<0.001	42.5	115.8	1.5	0.931	-31.8	34.7
**Haloperidol**	49.0 (47.2 to 50.7)	7.0	0.069	-0.4	14.5	7.3	0.058	-0.1	14.7	5.6	0.118	-1.3	12.6
**Lurasidone**	38.0 (36.3 to 39.7)	5.5	0.152	-1.9	12.8	-0.3	0.939	-7.6	7.0	0.6	0.868	-6.0	7.1
**Olanzapine**	424.5 (413.4 to 435.6)	11.0	0.589	-28.6	50.6	-38.8	0.063	-78.8	1.3	-2.3	0.903	-39.2	34.6
**Quetiapine**	189.0 (183.9 to 194.1)	4.3	0.707	-17.8	26.3	-11.3	0.325	-33.4	10.9	-8.8	0.385	-28.5	10.9
**Risperidone**	238.2 (232.8 to 243.7)	-11.0	0.364	-34.4	12.5	-32.0	0.009	-55.2	-8.7	-5.4	0.625	-27.0	16.2
**Sulpiride**	7.4 (6.7 to 8.0)	-2.3	0.092	-5.0	0.3	0.4	0.766	-2.2	3.1	-0.3	0.778	-2.7	2.0
**Zuclopenthixol**	9.8 (9.1 to 10.6)	-2.1	0.209	-5.3	1.1	0.9	0.573	-2.3	4.1	0.0	0.985	-2.8	2.8
Number of antipsychotic depot mentions	**Mean (95% Confidence Interval)**	**β coefficient: mean number of weekly depots**	**p**	**Lower limit**	**Upper limit**	**β coefficient: number of weekly depots**	**p**	**Lower limit**	**Upper limit**	**β coefficient: number of weekly depots**	**p**	**Lower limit**	**Upper limit**
**Aripiprazole depot**	115.6 (111.8 to 119.4)	31.9	<0.001	15.6	48.1	17.4	0.040	1.2	33.5	32.4	<0.001	17.9	46.8
**Paliperidone depot**	214.4 (207.9 to 221.0)	8.3	0.588	-21.6	38.2	0.3	0.741	-1.3	1.9	24.6	0.059	-0.4	49.6
**Haloperidol depot**	69.6 (67.5 to 71.7)	-4.8	0.303	-14.0	4.3	7.3	0.058	-0.1	14.7	-4.6	0.281	-12.9	3.7
**Risperidone depot**	38.4 (36.8 to 39.9)	-7.1	0.038	-13.6	-0.5	-6.3	0.060	-12.8	0.1	-4.3	0.176	-10.6	1.9
**Zuclopenthixol depot**	111.2 (109.0 to 113.4)	4.5	0.373	-5.3	14.4	-6.2	0.223	-16.1	3.7	4.2	0.342	-4.4	12.7
Number of mood stabiliser mentions	**Mean (95% Confidence Interval)**	**β coefficient: mean number of weekly mood stabilisers**	**p**	**Lower limit**	**Upper limit**	**β coefficient: number of weekly mood stabilisers**	**p**	**Lower limit**	**Upper limit**	**β coefficient: number of weekly mood stabilisers**	**p**	**Lower limit**	**Upper limit**
**Any mood stabiliser**	405.1 (395.7 to 414.5)	-17.8	0.391	-58.2	22.6	-31.3	0.137	-72.1	9.4	-36.5	0.053	-72.7	-0.3
Number of individual mood stabiliser mentions	**Mean (95% Confidence Interval)**	**β coefficient: mean number of weekly mood stabilisers**	**p**	**Lower limit**	**Upper limit**	**β coefficient: number of weekly mood stabilisers**	**p**	**Lower limit**	**Upper limit**	**β coefficient: number of weekly mood stabilisers**	**p**	**Lower limit**	**Upper limit**
**Carbamazepine**	20.8 (19.4 to 22.1)	-1.0	0.729	-6.9	4.8	-6.3	0.041	-12.2	-0.4	-6.2	0.025	-11.4	-0.9
**Lamotrigine**	116.7 (113.1 to 120.4)	-14.2	0.084	-30.1	1.6	-17.5	0.032	-33.1	-1.9	-14.5	0.046	-28.5	-0.6
**Lithium**	112.4 (109.3 to 115.4)	-1.8	0.786	-15.1	11.4	2.9	0.673	-10.5	16.3	-0.1	0.981	-11.9	11.6
**Valproate**	191.9 (187.3 to 196.4)	-5.4	0.588	-24.7	13.9	-15.4	0.140	-35.5	4.8	-17.5	0.058	-35.2	0.2

Variable	June 2020				July 2020				August 2020				September 2020			
Number of patients enrolled in mental health services	**β coefficient: number of patients**	**p**	**95% CI Lower limit**	**95% CI Upper limit**	**β coefficient: number of patients**	**p**	**95% CI Lower limit**	**95% CI Upper limit**	**β coefficient: number of patients**	**p**	**95% CI Lower limit**	**95% CI Upper limit**	**β coefficient: number of patients**	**p**	**95% CI Lower limit**	**95% CI Upper limit**
**Total**	-916.2	0.001	-1425.7	-406.8	-688.4	0.005	-1143.9	-232.9	-778.7	0.004	-1288.3	-269.1	-901.3	0.004	-1489.5	-313.0
**Female**	-461.1	0.004	-763.1	-159.2	-394.6	0.006	-664.7	-124.5	-398.2	0.013	-700.9	-95.5	-457.4	0.013	-806.8	-108.1
**Male**	-520.8	<0.001	-731.2	-310.5	-364.0	<0.001	-551.8	-176.2	-401.3	<0.001	-612.0	-190.7	-464.1	<0.001	-707.2	-220.9
**Under 18 years**	485.5	0.011	122.9	848.1	547.1	0.002	222.8	871.5	572.1	0.003	211.6	932.6	480.8	0.028	64.5	897.2
**18 to 64 years**	-967.0	<0.001	-1163.3	-770.6	-832.7	<0.001	-1007.8	-657.6	-951.2	<0.001	-1149.5	-752.9	-1020.1	<0.001	-1248.2	-792.1
**65 years and older**	-495.4	<0.001	-536.8	-454.0	-460.8	<0.001	-498.0	-423.6	-403.5	<0.001	-444.6	-362.4	-366.3	<0.001	-413.7	-318.9
Number of contacts with mental health professionals	**β coefficient: number of weekly contacts**	**p**	**Lower limit**	**Upper limit**	**β coefficient: number of weekly contacts**	**p**	**Lower limit**	**Upper limit**	**β coefficient: number of weekly contacts**	**p**	**Lower limit**	**Upper limit**	**β coefficient: number of weekly contacts**	**p**	**Lower limit**	**Upper limit**
**In person**	-4902.6	<0.001	-5994.2	-3810.9	-4421.6	<0.001	-5396.5	-3446.7	-4388.9	<0.001	-5478.9	-3299.0	-4401.4	<0.001	-5661.8	-3141.1
**Remote**	5190.0	<0.001	4890.3	5489.7	4914.0	<0.001	4676.1	5151.8	5571.0	<0.001	5308.0	5834.1	5008.2	<0.001	4672.4	5344.0
**Did not attend (DNA)**	-222.4	<0.001	-417.1	-27.6	-139.1	0.118	-310.9	32.8	-173.5	0.082	-365.0	18.1	-278.9	0.018	-502.5	-55.3
Number of antipsychotic mentions	**β coefficient: number of weekly antipsychotics**	**p**	**Lower limit**	**Upper limit**	**β coefficient: number of weekly antipsychotics**	**p**	**Lower limit**	**Upper limit**	**β coefficient: number of weekly antipsychotics**	**p**	**Lower limit**	**Upper limit**	**β coefficient: number of weekly antipsychotics**	**p**	**Lower limit**	**Upper limit**
**Any antipsychotic**	104.8	0.189	-49.5	259.0	122.4	0.087	-15.1	259.9	102.7	0.325	-99.8	305.2	3.0	0.980	-231.0	237.0
**Any depot**	21.9	0.412	-30.0	73.7	32.5	0.177	-14.0	79.1	59.3	0.110	12.3	130.9	17.2	0.683	-65.2	99.6
**Any oral**	81.8	0.208	-43.9	207.6	92.4	0.112	-19.8	204.7	51.9	0.522	-106.1	210.0	-17.3	0.854	-200.8	166.2
Number of individual antipsychotic mentions	**β coefficient: number of weekly antipsychotics**	**p**	**Lower limit**	**Upper limit**	**β coefficient: number of weekly antipsychotics**	**p**	**Lower limit**	**Upper limit**	**β coefficient: number of weekly antipsychotics**	**p**	**Lower limit**	**Upper limit**	**β coefficient: number of weekly antipsychotics**	**p**	**Lower limit**	**Upper limit**
**Amisulpiride**	0.7	0.912	-11.9	13.3	-4.3	0.455	-15.5	6.9	-3.5	0.603	-16.8	9.7	-15.9	0.047	-31.2	-0.6
**Aripiprazole**	31.4	0.130	-8.7	71.4	38.2	0.041	2.4	74.0	18.1	0.495	-33.6	69.8	-5.5	0.857	-65.3	54.2
**Clozapine**	18.2	0.342	-19.0	55.4	10.9	0.507	-21.0	42.8	31.4	0.100	-5.3	68.1	5.1	0.813	-37.2	47.5
**Haloperidol**	2.0	0.596	-5.4	9.5	-3.2	0.352	-9.8	3.4	-7.6	0.087	-16.2	1.0	-1.4	0.785	-11.8	8.9
**Lurasidone**	1.2	0.745	-6.0	8.5	4.0	0.238	-2.6	10.5	2.9	0.472	-5.0	10.8	-2.6	0.581	-11.8	6.6
**Olanzapine**	13.5	0.513	-26.7	53.7	13.7	0.457	-22.1	49.5	-0.6	0.983	-53.0	51.9	-10.3	0.742	-71.6	50.9
**Quetiapine**	25.5	0.025	3.8	47.2	17.0	0.103	-3.1	37.1	8.3	0.551	-18.7	35.2	4.9	0.758	-26.2	36.1
**Risperidone**	4.8	0.688	-18.4	28.0	3.8	0.728	-17.4	25.0	-20.5	0.183	-50.3	9.3	-23.5	0.190	-58.2	11.2
**Sulpiride**	0.9	0.511	-1.8	3.6	-1.1	0.360	-3.6	1.3	-1.7	0.230	-4.4	1.0	-1.7	0.291	-4.8	1.4
**Zuclopenthixol**	1.2	0.467	-2.0	4.3	2.6	0.080	-0.3	5.4	3.3	0.052	0.0	6.6	-0.4	0.824	-4.1	3.3
Number of antipsychotic depot mentions	**β coefficient: number of weekly depots**	**p**	**Lower limit**	**Upper limit**	**β coefficient: number of weekly depots**	**p**	**Lower limit**	**Upper limit**	**β coefficient: number of weekly depots**	**p**	**Lower limit**	**Upper limit**	**β coefficient: number of weekly depots**	**p**	**Lower limit**	**Upper limit**
**Aripiprazole depot**	26.4	0.003	9.6	43.1	32.8	<0.001	17.6	47.9	41.5	<0.001	23.6	59.5	37.6	0.001	16.7	58.6
**Paliperidone depot**	7.3	0.618	-21.3	35.9	12.8	0.326	-12.5	38.1	18.4	0.242	-12.1	49.0	-8.0	0.655	-42.9	26.9
**Haloperidol depot**	-5.1	0.291	-14.5	4.3	-3.6	0.405	-12.0	4.8	-5.3	0.393	-17.3	6.7	-3.0	0.671	-16.9	10.8
**Risperidone depot**	-13.6	<0.001	-20.1	-7.1	3.8	0.728	-17.4	25.0	-9.7	0.008	-16.6	-2.8	-9.8	0.018	-17.7	-1.9
**Zuclopenthixol depot**	0.3	0.957	-9.4	10.0	-2.6	0.565	-11.5	6.3	12.4	0.122	-3.1	27.8	4.9	0.596	-13.1	22.8
Number of mood stabiliser mentions	**β coefficient: number of weekly mood stabilisers**	**p**	**Lower limit**	**Upper limit**	**β coefficient: number of weekly mood stabilisers**	**p**	**Lower limit**	**Upper limit**	**β coefficient: number of weekly mood stabilisers**	**p**	**Lower limit**	**Upper limit**	**β coefficient: number of weekly mood stabilisers**	**p**	**Lower limit**	**Upper limit**
**Any mood stabiliser**	-8.3	0.686	-48.4	31.8	-3.9	0.833	-39.7	32.0	-0.3	0.989	-48.8	48.1	-26.6	0.361	-83.2	30.0
Number of individual mood stabiliser mentions	**β coefficient: number of weekly mood stabilisers**	**p**	**Lower limit**	**Upper limit**	**β coefficient: number of weekly mood stabilisers**	**p**	**Lower limit**	**Upper limit**	**β coefficient: number of weekly mood stabilisers**	**p**	**Lower limit**	**Upper limit**	**β coefficient: number of weekly mood stabilisers**	**p**	**Lower limit**	**Upper limit**
**Carbamazepine**	-8.3	0.007	-14.1	-2.5	-3.4	0.211	-8.6	1.9	-8.3	0.009	-14.3	-2.3	-9.3	0.012	-16.3	-2.3
**Lamotrigine**	-7.0	0.385	-22.6	8.6	-5.1	0.476	-19.1	8.9	-9.2	0.308	-26.7	8.3	-20.8	0.049	-41.0	-0.5
**Lithium**	5.4	0.426	-7.8	18.6	9.5	0.122	-2.3	21.2	6.6	0.409	-9.0	22.2	8.6	0.363	-9.8	27.0
**Valproate**	-6.4	0.525	-25.9	13.1	-8.1	0.368	-25.5	9.4	12.2	0.293	-10.3	34.8	-5.7	0.673	-32.1	20.7

< 0.001	<0.01	<0.05
