# Supplementary material for: Impact of the COVID-19 pandemic on remote mental healthcare and prescribing in psychiatry: an electronic health record study
Source: BMJ Open. 2021 Mar 30;11(3):e046365. doi: 10.1136/bmjopen-2020-046365 (PMC8728386; doi:10.1136/bmjopen-2020-046365)
Supplement: Author's manuscript [file bmjopen-2020-046365.draft_revisions.pdf]

BMJ Open is committed to open peer review. As part of this commitment we make the peer review history of every article we publish publicly available.

When an article is published we post the peer reviewers' comments and the authors' responses online. We also post the versions of the paper that were used during peer review. These are the versions that the peer review comments apply to.

The versions of the paper that follow are the versions that were submitted during the peer review process. They are not the versions of record or the final published versions. They should not be cited or distributed as the published version of this manuscript.

BMJ Open is an open access journal and the full, final, typeset and author-corrected version of record of the manuscript is available on our site with no access controls, subscription charges or pay-per-view fees (http://bmjopen.bmj.com).

If you have any questions on BMJ Open's open peer review process please email info.bmjopen@bmj.com

BMJ Open

Impact of the COVID-19 pandemic on remote mental healthcare and prescribing in psychiatry

Journal:	*BMJ Open*
Manuscript ID	bmjopen-2020-046365
Article Type:	Original research
Date Submitted by the Author:	28-Oct-2020
Complete List of Authors:	Patel, Rashmi; King's College London Institute of Psychiatry Psychology and Neuroscience, Department of Psychosis Studies<br>Irving, Jessica; King's College London Institute of Psychiatry Psychology and Neuroscience, Department of Psychosis Studies<br>Brinn, Aimee; King's College London Institute of Psychiatry Psychology and Neuroscience, Department of Psychosis Studies<br>Broadbent, Matthew; South London and Maudsley Mental Health NHS Trust<br>Shetty, Hitesh; South London and Maudsley Mental Health NHS Trust, Biomedical Research Centre Nucleus<br>Pritchard, Megan; South London and Maudsley Mental Health NHS Trust, Institute of Psychiatry, Psychology and Neuroscience<br>Downs, Johnny; King's College London Institute of Psychiatry Psychology and Neuroscience<br>Stewart, Robert; King's College London Institute of Psychiatry Psychology and Neuroscience<br>Harland, Robert; South London and Maudsley Mental Health NHS Trust<br>McGuire, Philip; King's College London Institute of Psychiatry Psychology and Neuroscience, Department of Psychosis Studies
Keywords:	EPIDEMIOLOGY, Telemedicine < BIOTECHNOLOGY & BIOINFORMATICS, Health informatics < BIOTECHNOLOGY & BIOINFORMATICS, MENTAL HEALTH, PSYCHIATRY

SCHOLARONE™
Manuscripts

I, the Submitting Author has the right to grant and does grant on behalf of all authors of the Work (as defined in the below author licence), an exclusive licence and/or a non-exclusive licence for contributions from authors who are: i) UK Crown employees; ii) where BMJ has agreed a CC-BY licence shall apply, and/or iii) in accordance with the terms applicable for US Federal Government officers or employees acting as part of their official duties; on a worldwide, perpetual, irrevocable, royalty-free basis to BMJ Publishing Group Ltd ("BMJ") its licensees and where the relevant Journal is co-owned by BMJ to the co-owners of the Journal, to publish the Work in this journal and any other BMJ products and to exploit all rights, as set out in our licence.

The Submitting Author accepts and understands that any supply made under these terms is made by BMJ to the Submitting Author unless you are acting as an employee on behalf of your employer or a postgraduate student of an affiliated institution which is paying any applicable article publishing charge ("APC") for Open Access articles. Where the Submitting Author wishes to make the Work available on an Open Access basis (and intends to pay the relevant APC), the terms of reuse of such Open Access shall be governed by a Creative Commons licence – details of these licences and which Creative Commons licence will apply to this Work are set out in our licence referred to above.

Other than as permitted in any relevant BMJ Author's Self Archiving Policies, I confirm this Work has not been accepted for publication elsewhere, is not being considered for publication elsewhere and does not duplicate material already published. I confirm all authors consent to publication of this Work and authorise the granting of this licence.

For peer review only

Impact of the COVID-19 pandemic on remote mental healthcare and prescribing in psychiatry

Rashmi Patel*[1,2], Jessica Irving*[1], Aimee Brinn[1], Matthew Broadbent[2], Hitesh Shetty[2], Megan Pritchard[1,2], Johnny Downs[1,2], Robert Stewart[1,2], Robert Harland[2], Philip McGuire[1,2]

1. King's College London (Institute of Psychiatry, Psychology and Neuroscience), London, UK
2. South London and Maudsley NHS Foundation Trust, London, UK

*Equal contributions to authorship

Correspondence to:

Rashmi Patel

E-mail: bmj@rpatel.co.uk

Manuscript word count (excluding references): 4,395

Abstract word count: 281

Keywords: SARS-CoV2, telepsychiatry, telemedicine, electronic health records, mental health

Licence declaration: I, the Submitting Author has the right to grant and does grant on behalf of all authors of the Work (as defined in the below author licence), an exclusive licence and/or a non-exclusive licence for contributions from authors who are: i) UK Crown employees; ii) where BMJ has agreed a CC-BY licence shall apply, and/or iii) in accordance with the terms applicable for US Federal Government officers or employees acting as part of their official duties; on a worldwide, perpetual, irrevocable, royalty-free basis to BMJ Publishing Group Ltd ("BMJ") its licensees and where the relevant Journal is co-owned by BMJ to the co-owners of the Journal, to publish the Work in BMJ Open and any other BMJ products and to exploit all rights, as set out in our licence.

The Submitting Author accepts and understands that any supply made under these terms is made by BMJ to the Submitting Author unless you are acting as an employee on behalf of your employer or a postgraduate student of an affiliated institution which is paying any applicable article publishing charge ("APC") for Open Access articles. Where the Submitting Author wishes to make the Work available on an Open Access basis (and intends to pay the relevant APC), the terms of reuse of such Open Access shall be governed by a Creative Commons licence – details of these licences and which Creative Commons licence will apply to this Work are set out in our licence referred to above.

For peer review only - http://bmjopen.bmj.com/site/about/guidelines.xhtml

For peer review only

Competing interests: All authors have completed the ICMJE uniform disclosure form at www.icmje.org/coi_disclosure.pdf and declare: RS has received funding from Janssen, GSK and Takeda outside the submitted work. RP has received funding from Janssen, Induction Healthcare and Holmusk outside the submitted work. The other authors declare no competing interests.

Author contributions: The study was conceived by RP. Data extraction and statistical analysis were performed by JI supervised by RP and supported by MB and HS. Reporting of findings were carried out by RP, JI and AB. All authors (RP, JI, AB, MB, HS, MP, JD, RS, RH, PM) contributed to study design, manuscript preparation and approved the final version.

Ethics approval: The CRIS data resource received ethical approval as a de-identified dataset for secondary mental health research analyses from Oxfordshire REC C (Ref: 18/SC/0372).

Source of funding: MB, HS, MP, RS and PM have received funding from the National Institute for Health Research (NIHR) Biomedical Research Centre at South London and Maudsley NHS Foundation Trust and King's College London, which also supports the development and maintenance of the BRC Case Register. RP has received funding from a Medical Research Council (MRC) Health Data Research UK Fellowship (MR/S003118/1) and a Starter Grant for Clinical Lecturers (SGL015/1020) supported by the Academy of Medical Sciences, The Wellcome Trust, MRC, British Heart Foundation, Arthritis Research UK, the Royal College of Physicians and Diabetes UK. RS has received funding from a Medical Research Council (MRC) Mental Health Data Pathfinder Award to King's College London, an NIHR Senior Investigator Award and the National Institute for Health Research (NIHR) Applied Research Collaboration South London (NIHR ARC South London) at King's College Hospital NHS Foundation Trust.

Role of funder: The views expressed are those of the authors and not necessarily those of the funders. The funders had no role in the design and conduct of the study; collection, management, analysis, and interpretation of the data; preparation, review, or approval of the manuscript; and decision to submit the manuscript for publication.

Data sharing: The data accessed by CRIS remain within an NHS firewall and governance is provided by a patient-led oversight committee. Access to data is restricted to honorary or substantive employees of the South London and Maudsley NHS Foundation Trust and governed by a local oversight committee who review and approve applications to extract and analyse data for research. Subject to these conditions, data access is encouraged and those interested should contact RS (robert.stewart@kcl.ac.uk), CRIS academic lead.

For peer review only - http://bmjopen.bmj.com/site/about/guidelines.xhtml

Abstract

Objectives:

The recent COVID-19 pandemic has disrupted mental healthcare delivery, with many services shifting from in-person to remote patient contact. We investigated the impact of the pandemic on the use of remote consultation and on the prescribing of psychiatric medications.

Design and setting:

The Clinical Record Interactive Search tool (CRIS) was used to examine de-identified electronic health records (EHRs) of people receiving mental healthcare from the South London and Maudsley (SLaM) NHS Foundation Trust. Data from the period before and after the onset of the pandemic were analysed using linear regression, and visualised using locally estimated scatterplot smoothing (LOESS).

Participants:

All patients receiving care from SLaM between 7th January 2019 and 20th September 2020 (around 37,500 patients per week).

Outcome measures:

i) The number of clinical contacts (in-person, remote or non-attended) with mental healthcare professionals per week

ii) Prescribing of antipsychotic and mood stabiliser medications per week

Results:

Following the onset of the pandemic, the frequency of in-person contacts was significantly reduced compared to that in the previous year (β coefficient: -5829.6 contacts, 95% CI -6919.5 to -4739.6, p<0.001), while the frequency of remote contacts significantly increased (β coefficient: 3338.5 contacts, 95% CI 3074.4 to 3602.7, p<0.001). Rates of remote consultation were lower in older adults than in working age adults, children and adolescents. Despite this change in the type of patient contact, antipsychotic and mood stabiliser prescribing remained at similar levels.

Conclusions:

The COVID-19 pandemic has been associated with a marked increase in remote consultation, particularly among younger patients. However, there was no evidence that this has led to changes in psychiatric prescribing. Nevertheless, further work is needed to ensure that older patients are able to access mental healthcare remotely.

For peer review only - http://bmjopen.bmj.com/site/about/guidelines.xhtml

Article Summary

Strengths and limitations

- In response to the COVID-19 pandemic, many mental health services have moved to remote consultation, without knowing if this has is having an adverse effect on clinical care.
- The present study was able to address this issue, by using electronic health record data to rapidly analyse the impact of the pandemic in a large sample of patients.
- Employing natural language processing (NLP) tools provided access to data on medication prescribing from routine clinical records.
- A short follow-up period precluded the investigation of associations between remote consultation and diagnoses and clinical outcomes.

For peer review only

For peer review only - http://bmjopen.bmj.com/site/about/guidelines.xhtml

Introduction

The COVID-19 pandemic caused by the SARS-CoV-2 coronavirus, and accompanying physical distancing and travel restrictions, has had a tremendous impact on the delivery of healthcare worldwide. It has particularly affected mental healthcare, making it more difficult for people to be seen in-person and harder to deliver treatments.[1–3] The pandemic has also increased the exposure of patients to factors known to exacerbate mental health disorders, including isolation, unemployment, inactivity and decreased social support.[4] The extent to which these changes are impacting on mental health is unknown,[5] but is critical to the ongoing reconfiguration of services in response to the current pandemic.[4]

The recent advent of technology to support remote communication in healthcare (telemedicine)[6] has enabled a shift from in-person to remote clinical contact in response to the COVID-19 pandemic. This has facilitated healthcare following the adoption of travel and physical distancing restrictions originally developed by the UK government in response to the spread of SARS-CoV-2.[7]

Prior to the COVID-19 pandemic, the application of telemedicine in mental health has been limited, and little is known about the clinical and sociodemographic factors that may influence the effectiveness of remote consultation in this context.[8] As the current pandemic evolves, while travel and physical distancing restrictions are expected to continue,[9–11] remote consultation is likely to constitute a major component of mental healthcare until the pandemic is over. Furthermore, it is possible that in the absence of a pandemic, remote consultation could help to improve access to and reduce costs of delivering community mental healthcare.[7] Thus, it is critical to determine the impact, potential benefits and disadvantages of remote consultation in mental healthcare.

Electronic health record (EHR) studies provide an opportunity to look at patterns of health care service delivery across populations.[12] Natural language processing (NLP) techniques can be used to enrich EHR datasets by extracting clinical relevant data from assessments, reviews and correspondence documented as unstructured free text.[13–16] Web-based data visualisation platforms can then be used to present population data derived from EHRs to support healthcare policy making.[17,18]

In the present study, we analysed EHR data from a large provider of mental healthcare in South London (UK) to assess the impact of adopting remote consultation in response to the current COVID-19 pandemic. We tested the hypothesis that moving to remote consultation could be achieved without compromising the delivery of key components of secondary mental health care.

For peer review only - http://bmjopen.bmj.com/site/about/guidelines.xhtml

Methods

Participants and setting

We extracted data from de-identified EHRs for individuals accessing secondary mental health care within the South London and Maudsley (SLaM) NHS Foundation Trust from 7th January 2019 to 20th September 2020. SLaM holds EHRs for over 450,000 people who have received mental healthcare since 2007.[12] The Trust provides community and inpatient mental healthcare within its catchment area which includes the London boroughs of Lambeth, Southwark, Croydon and Lewisham.

Data extraction

We extracted separate datasets for each week from January 2019 to September 2020. We chose this time period to provide a whole calendar year of data representing typical clinical activity prior to the onset of the COVID-19 pandemic, which was declared by the World Health Organisation in March 2020.[19] The cohort for each week included all patients with an active referral to SLaM, defined as having been accepted to a SLaM community or inpatient service on, or before the Sunday of each week and having been discharged on, or after the Monday of the same week (for those patients discharged from SLaM clinical services prior to September 2020). This method was chosen to enable assessment of changes in the frequency of data in the context of the number of active patients within a given week, taking into account fluctuations in rates of acceptance to, and discharge from clinical services during the period of data analysis, and the fact that some patients may have had an active referral to SLaM for a duration of less than a week (e.g. patients who receive a single assessment but are then discharged without further follow-up).

The Clinical Record Interactive Search (CRIS) tool was used to extract data. CRIS is a bespoke platform and governance framework to support an electronic mental health case register whose infrastructure is based on Microsoft Structured Query Language (SQL) and provides an automated pipeline for EHR data de-identification, database assembly and query to facilitate analyses of EHR-derived data of patients receiving care from SLaM.[12,20] The source EHR data are generated by SLaM clinicians, including psychiatrists, psychologists, nurses and other health and social care professionals who document clinical assessments, reviews and progress in the provision of mental healthcare in the community or psychiatric hospitals. The dataset has received ethical approval from Oxfordshire REC C (Ref: 18/SC/0372) for secondary analysis of de-identified EHR data to support mental health research. Access to data is restricted to honorary or substantive employees of SLaM.

Patient and public involvement

The project was approved by the local SLaM CRIS Oversight Committee (chaired by a service user representative) who review and approve applications to extract data for research. The CRIS infrastructure was designed and is managed with ongoing service user input to ensure all research projects comply with data governance, legal and ethical guidelines. Further details are available on the SLaM CRIS website.[21]

Defined variables

Data were extracted on the number of active patients at any point in a given week (referred to as "patients registered with SLaM services"), age, gender, the number of contacts with mental healthcare professionals which were attended in-person, remotely or where a patient did not attend (DNA), and psychotropic medications (antipsychotic drugs and mood stabilisers).

For peer review only - http://bmjopen.bmj.com/site/about/guidelines.xhtml

For peer review only

We defined 'in-person' contacts as attended appointments with a clinician either individually or in a group setting. 'Remote' contacts included e-mail, short message service (SMS) text, letter, telephone or video call. 'Did not attend' (DNA) contacts were defined as all unplanned appointment cancellations (in-person or remote).

Age was determined according to the age of each active patient on the Sunday of each week and was categorised in groups defined as under 18 years, 18 to 64 years and 65 years and over. These groups were chosen to reflect the age group structure of SLaM clinical services which provide care for children and adolescents, working age adults and older adults respectively.[12]

We extracted recorded mentions of antipsychotics and mood stabilisers using two sources: structured input fields within the EHR to record prescribed psychotropic medications and natural language processing (NLP) tools applied to free text EHR data including documentation of clinical assessments, and progress notes, clinical reports and correspondence between clinicians. The development of these NLP tools and example use cases have been previously described;[12,16,22,23] details on manual validation can be found on the CRIS website.[24]

Antipsychotics were defined according to the Maudsley Guidelines for Advanced Prescribing in Psychosis[25] and grouped as oral antipsychotics and long-acting injectable (LAI) depot antipsychotics. A further analysis of the most frequently prescribed antipsychotics was conducted including amisulpride, aripiprazole, clozapine, haloperidol, lurasidone, quetiapine, olanzapine, risperidone, sulpiride, zuclopenthixol, aripiprazole LAI, paliperidone LAI (including both 1-monthly and 3-monthly preparations), haloperidol LAI, risperidone LAI and zuclopenthixol LAI. Non-antipsychotic mood stabilisers were defined as carbamazepine, lamotrigine, lithium and valproate. The analyses focused on antipsychotics and mood stabilisers because these are the most frequently prescribed psychotropic medications in UK secondary mental healthcare. We chose not to analyse data on antidepressants, as these are often initiated in primary care and these data are not comprehensively represented in the secondary mental healthcare dataset analysed in this study.

Data analysis and visualisation

Data were cleaned and visualised using Exploratory Version 6.2.2 (https://docs.exploratory.io/), a user interface frontend to R 4.0.x for data analysis and visualisation. Results were communicated via an interactive dashboard which enables users to zoom and scroll within charts to highlight specific data points or series. This platform also facilitates updates to the dataset over time beyond the date of publication of this article and enables rapid visualisation of data to inform future healthcare policy. A brief guide on how to visualise data from the present study (available at http://rpatel.co.uk/TelepsychiatryDashboard) is provided in the supplementary material.

Based on the declaration of the COVID-19 pandemic in March 2020, we chose an index period of Monday 4th March 2019 to Sunday 1st March 2020 against which to compare subsequent data. We estimated the mean and 95% confidence interval for all weeks within the index period for each analysed variable. We performed separate univariate linear regression analyses to compare the weekly counts for each variable in each of the calendar months of March, April, May, June, July, August and September 2020 against the mean weekly counts in the index period. The β coefficient of each analyses represents the estimated difference from the index period mean for each variable within each analysed month. This method of monthly comparisons was chosen to enable an estimation of changes in frequency of each variable after the onset of the pandemic while taking into account weekly variations which would have occurred due to the non-working days of Easter in April and the UK bank holidays during May.

Data were aggregated and presented in charts using Exploratory software. Each data point in a given chart represents the count for the analysed variable on the Sunday of each week. Locally estimated scatterplot smoothing (LOESS) was used to superimpose a smoothed trendline (represented by a dotted line and shaded area for the confidence interval). LOESS was used to smooth out variation between each week and highlight data points for weeks indicating significant variation away from the lower or upper bound of the confidence

For peer review only - http://bmjopen.bmj.com/site/about/guidelines.xhtml

interval. In order to effectively visualise data in limited screen space, the y-axes of charts were adjusted to fit the majority of the data points within the viewable area for each chart. When viewing the results, care should be taken to examine the y-axis to determine scale and intercept prior to interpreting data points and fitted LOESS curves within the charts.

For peer review only

For peer review only - http://bmjopen.bmj.com/site/about/guidelines.xhtml

Results

Patients registered with mental health services

A mean number of 37,563 patients (95% CI 37,420 to 37,707) were registered with clinical services each week during the index period. Regression analyses indicated consistent decreases for each month of April 2020 to September 2020 in the number of patients registered per week relative to the index period, with the exception of registration for those under 18 years, which showed the opposite trend (Supplementary Table 1, Figure 1 and Supplementary Figure 1). However, the relative changes in the total number of patients registered with SLaM services during each week after April 2020 were modest, representing a maximum change of around 2% following the index period (Figure 1 and Supplementary Figure 1).

Contacts with mental health professionals

From January 2019 to February 2020, the number of weekly contacts reduced considerably around late December to early January, reflecting a slowdown in clinical activity during the Christmas and New Year period (Figure 2). From March 2020, in-person contacts reduced substantially from around 9,000 per week to 3,000 per week in early April 2020, corresponding with the implementation of travel and physical distancing restrictions. Over the same period there was an substantial increase in remote contacts from around 2,500 per week in early March 2020 to around 8,000 per week by the end of April 2020 (Figure 3), while the total number of clinical contacts per week dropped from around 12,500 in mid-March to around 10,000 in mid-April 2020 (Figure 2).

These visual trends were confirmed through linear regression analyses, which found consistent decreases in mean weekly in-person contacts each month from March to September relative to the previous year. This was matched by consistent increases in remote contacts compared with the previous year. The number of unattended appointments was temporarily reduced in April, May, June and September 2020 (Supplementary Table 1).

It is notable that while there were reductions in clinical contacts during the early phase of the COVID-19 pandemic, these were smaller than those observed during the Christmas and New Year period (December 2019/January 2020) prior to the pandemic.

Figure 4 illustrates the breakdown of clinical contacts per week by age. Children and adolescents under 18, working age adults and older adults show varying degrees of reduction in weekly contacts following the onset of the COVID-19 pandemic. The total number of clinical contacts for children and adolescents between mid-March and mid-May reduced to a lesser degree than for working age adults and older adults.

Figure 5 indicates a compensatory increase in remote consultations for children and adolescents, with a less pronounced increase seen in patients of working age and in older adults. Although there was no clear difference between number of weekly clinical events by gender (Supplementary Figure 2), there was a greater increase in remote consultations following March 2020 in female than male patients (Supplementary Figure 3).

Antipsychotics

Visual inspection of antipsychotic reporting trends yielded two main findings: first, there was no evidence of a substantial change in total antipsychotic mentions in EHRs following the onset of the pandemic (for either oral or LAI depot antipsychotics). Second, there was a large decrease in mentions of antipsychotics over Christmas and the New Year period (Supplementary Figure 4). When grouped by age, antipsychotic mentions reduced during 2019, before increasing towards January 2020 in children and adolescents. Working age adults showed a reduction in antipsychotic mentions during 2019, with a nadir around the Christmas and New Year period. In

For peer review only - http://bmjopen.bmj.com/site/about/guidelines.xhtml

contrast, antipsychotic mentions steadily increased among older adults over 65 (Supplementary Figure 5). There were no clear differences in antipsychotic mentions by gender (Supplementary Figure 6).

There was a statistically significant and consistent increase in weekly aripiprazole depot mentions in the months of March 2020 to September 2020 compared with mean rates across the index period. Risperidone depot mentions were reduced in March, June, August and September 2020 compared to the index period. A statistically significant increase in clozapine mentions was observed in April only ($\beta = 79.2$, $p < 0.001$) compared with the previous year (Supplementary Table 1).

Mood stabilisers

Total mentions of mood stabilisers slowly declined from the beginning of the observation period in January 2019 (Supplementary Figures 7, 8 and 9). In terms of individual mood stabilisers, from January 2019, lithium mentions increased and mentions of valproate and lamotrigine decreased (Supplementary Figure 7). Regression analysis found small decreases of marginal significance in carbamazepine and lamotrigine mentions between April 2020 and September 2020 relative to the pre-pandemic index period (Supplementary Table 1).

For peer review only

Discussion

We found that the onset of the COVID-19 pandemic was associated with a rapid and major shift in the mode of mental healthcare consultations, from in-person to remote in the early phase (between March and May 2020), followed by a gradual shift back towards in-person consultations over the following months. Reassuringly, these substantial changes do not appear to have been associated with dramatic shifts in the clinical activity of mental health services, as measured by the number of mentions of psychotropic medications in free text EHRs. A compensatory increase in remote mental health consultation in response to the COVID-19 pandemic mirrors similar developments within physical healthcare[26] and is in keeping with a previous analysis of working age adults using the same dataset.[27]

The shift from in-person to remote consultation was most pronounced for children and adolescents (and their parents and carers), less marked in working age adults, and least dramatic in older adults. Mental health services for older adults have not utilised remote methods for consultation to the same degree as services for working age adults and children, and this has been associated with a reduction in total clinical contacts during the early phase of the pandemic between March and May 2020. Importantly, some forms of remote contact rely on the availability of electronic hardware (a smartphone or computer), access to a stable electricity supply and high-speed internet connection, and the ability to use the technology. To enable successful remote consultation, these resources are required by both patients and clinicians, and both groups need to be able to trust the technology (with respect to safety, reliability and privacy) and be motivated to use it.[28]

From April 2020 the number of patients registered per week in mental health services decreased relative to the period of March 2019 to March 2020. This is in keeping with an earlier analysis of the same dataset examining the period from 1st February to 31st March 2020.[29] This reduction in patients registered following April 2020 may reflect the discharge of patients in the initial stages of the pandemic who may have previously had limited input from mental health services. This is supported by the fact that there was no demonstrable change in clinical activity measured through mentions of antipsychotic and mood stabiliser medications during the same period. Nonetheless, the relative reduction in number of active patients in a given week during the early stages of the pandemic was modest, representing around 2% of the total mean number of active patients between March 2019 and March 2020.

Throughout the pandemic period, the number of mentions of antipsychotics (whether oral or depot) remained steady. The only exception was an increase in mentions of aripiprazole depot, but this may reflect its recent addition to the NHS formulary. We found a reduction in mentions of antipsychotics for children and adolescents. This may be due to a reduction in the number of inpatient psychiatric beds for children and adolescents in SLaM due to the closure of two inpatient units during the same period which could have led to reduced documentation of antipsychotics in this patient group. It is important to note that change in frequency of clinical documentation of psychotropic medications is not necessarily correlated with changes in prescribing per se, and could reflect differences in the nature and frequency of EHR documentation between inpatient and community mental teams. We observed a steady increase in mentions of antipsychotics for older adults which predated the COVID-19 pandemic. Likewise, this gradual increase in documentation may or may not reflect a change in prescribing, and could be related to service reconfigurations associated with changing frequency of clinical documentation and the increasing focus on monitoring of antipsychotic use in dementia.

Clozapine prescribing differs from that for other antipsychotics, in that it requires regular blood monitoring, making in-person contact essential. The increase in clozapine mentions observed in April 2020 relative to the previous year may be due to the introduction of emergency guidelines regarding frequency of full blood count monitoring to manage possible disruptions during the initial period of travel and physical distancing restrictions.[30] The increase in mentions of clozapine in April could reflect increased activity among clinicians creating treatment plans to navigate the challenges of managing full blood count monitoring for these patients.

Our analysis also revealed a decreasing trend in overall mood stabiliser mentions over time that was not related to the COVID-19 pandemic period. Notably, lithium mentions have increased and mentions of

For peer review only - http://bmjopen.bmj.com/site/about/guidelines.xhtml

valproate and lamotrigine have decreased. The decreasing trend in valproate mentions may be explained by a reduction in prescriptions among female patients of child-bearing age due to increased awareness of its teratogenic effects.[31,32] It may be that the decrease in valproate prescribing is associated with a compensatory increase in mentions of lithium, or this could be related to efforts to increase access to lithium for people suffering with treatment resistant depression or bipolar disorder.[33,34]

Implications

The lack of digital technology resources among certain vulnerable groups creates barriers to accessing remote mental healthcare. People with low incomes (over-represented among people with serious mental disorders) may not be able to afford the necessary hardware/data connection.[35] Serious mental illnesses are associated with major impairments in motivation which impair the ability of affected individuals to use remote consultation technology.[36] And many people may not have had the opportunity to access training to gain skills in using digital healthcare tools.[28] Furthermore, people with serious mental illnesses are overrepresented in deprived areas, which are the very populations at increased risk for uncontained COVID-19 transmission resulting in greater travel and physical distancing restrictions which would necessitate remote consultation to gain access to mental healthcare.[7,37]

Our research indicated that the incidence of missed appointments decreased as appointments changed from in-person to remote between April and June. Adherence to a treatment plan is an essential component for successful mental healthcare management; for people diagnosed with schizophrenia, failure to attend outpatient care following admission increases risk of relapse and readmission.[38] Relapse of serious mental illness poses large additional costs the NHS: an estimate of four times greater costs for patients who relapse, compared to those who do not.[39] Considering non-attendance at mental health care appointments is reported to be greater than most other healthcare settings,[40,41] remote methods could be harnessed more often to reduce the financial burden of non-attended appointments.[41]

Future work must urgently identify how to remove the barriers faced in access to and ability to use digital technology for remote consultation. A Canadian study found that patients receiving telephone appointments felt able to present the same information as in-person and were equally as satisfied as patients receiving in-person appointments. However, the telepsychiatry group reported lower levels of support and encouragement than in-person patients.[42] Furthermore, a systematic review, which analysed the acceptability of online and mobile phone app interventions for serious mental illness, indicated that whilst hypothetical acceptability of online and mobile phone interventions was low, actual acceptability was generally high.[43] It is possible that certain groups may feel less able to communicate their mental health difficulties remotely, whilst others may find the opposite. At the same time, clinicians may feel less able to adequately assess the mental state of patients through remote technology compared to in-person assessment. Further in-depth, qualitative research is required to elucidate the views and experiences of different patient and clinician groups.

Strengths and limitations

The availability of electronic health records in SLaM has enabled the rapid development of a data visualisation platform to assess key metrics of mental health service delivery during a period of tremendous challenge to service provision during the COVID-19 pandemic. Our findings will support mental healthcare strategy to reduce barriers to those who facing difficulties accessing remote care. This data visualisation approach could be applied to other forms of EHR derived healthcare data on a real-time basis to enable healthcare service providers and policy makers to adapt service provision in time of crisis, and to measure the impact of changes to service provision on performance metrics such as access to remote care.

We were able to examine changes in the frequency of different types of mental health service consultation and mentions of psychotropic prescribing over time. However, it was not possible to analyse the nature of in-person and remote consultations. Remote consultations can take many forms and the ability to perform certain clinical tasks may vary depending on the modality employed. For example, e-mail and text messaging may enable communication between patients and clinicians, but opportunities for real-time clinical assessment and review are limited. In contrast, a phone call can provide some information on history and

For peer review only - http://bmjopen.bmj.com/site/about/guidelines.xhtml

mental state, but the availability of a video stream enables a more detailed assessment of mental state and environment. Certain clinical tasks may have been better suited to different forms of remote consultation. NLP could be used to interrogate the content of free text documentation of remote consultations and to compare these to documentation of in-person consultations to identify barriers to performing different clinical activities and how these could be overcome.

While we are able to examine associations with age and gender, it was not possible to meaningfully investigate variation in rates and nature of remote consultation based on psychiatric diagnosis owing to the short window of data available within the present study, and because diagnostic data are not comprehensively documented at every clinical encounter. A cohort study comparing people with different psychiatric diagnoses on the frequency of in-person, remote and non-attended consultations would help to determine which groups of patients and treating clinicians have more avidly taken up remote forms of consultation during the early phase of the pandemic and what can be done to ensure digital access across all clinical subgroups.

While case registers provide a general impression of medication prescribing, they cannot provide information on treatment adherence or prescribing rationale. Importantly, SLaM is a secondary mental health care setting. Patients with chronic serious mental disorder may however be predominantly managed by their general practitioner meaning their psychotropic medication prescribing may not be represented within secondary mental health EHR data. This is a consistent shortcoming in secondary care EHR research. Better linkages between primary and secondary care electronic health record systems at the individual patient level would help to facilitate more comprehensive analysis of psychotropic prescribing in people receiving secondary mental healthcare. The approach taken in the present study to examine psychotropic prescribing trends is only possible in healthcare settings with electronic case registers that capture prescribing data. Only ~50% of upper-middle- and high-income countries (n=23) have adopted national EHR systems (2016 data, World Health Organisation).[44] Adoption rates of EHRs are much lower in the lower-middle (35%; n=10) and low-income countries (15%; n=3). Adoption rates for psychiatric case registers for secondary analysis in research studies, such as that held by SLaM, are far lower.

While the present dashboard focuses on medication it will also be important to assess impacts on psychological and occupational therapies. One question is whether the shift to remote contacts is associated with changes to the delivery of psychological therapies and if certain groups of people are benefitting, or are marginalised, from remote therapies. This approach could be used to identify hard-to-reach groups and develop interventions tailored towards these groups and challenges they may face accessing remote care.

Conclusions

In summary, our findings indicate that the COVID-19 pandemic has led to a rapid shift from in-person to remote methods of mental healthcare consultation, without a significant change in the overall level of clinical activity. In addition, we found marked differences in the application of remote consultation by patient age, with lower rates of among older adults, and the highest among children and adolescents. Given that travel and physical distancing restrictions related to the COVID-19 pandemic are likely to persist to some degree in the near future, it is important to ensure all people who receive and provide mental healthcare have access to the digital technology, training and clinical and social support required to access remote consultation, which is likely to continue to be an important modality to support mental health service delivery in the years following the pandemic.

For peer review only - http://bmjopen.bmj.com/site/about/guidelines.xhtml

References

1 Chevance A, Gourion D, Hoertel N, *et al.* Ensuring mental health care during the SARS-CoV-2 epidemic in France: A narrative review. Encephale. 2020;**46**:193–201. doi:10.1016/j.encep.2020.04.005

2 Kozloff N, Mulsant BH, Stergiopoulos V, *et al.* The COVID-19 Global Pandemic: Implications for People With Schizophrenia and Related Disorders. *Schizophr Bull* 2020;**46**:752–7. doi:10.1093/schbul/sbaa051

3 Garriga M, Agasi I, Fedida E, *et al.* The role of mental health home hospitalization care during the COVID-19 pandemic. *Acta Psychiatr Scand* 2020;**141**:479–80. doi:10.1111/acps.13173

4 Moreno C, Wykes T, Galderisi S, *et al.* How mental health care should change as a consequence of the COVID-19 pandemic. *The Lancet Psychiatry* 2020;**0**. doi:10.1016/s2215-0366(20)30307-2

5 Holmes EA, O'Connor RC, Perry VH, *et al.* Multidisciplinary research priorities for the COVID-19 pandemic: a call for action for mental health science. The Lancet Psychiatry. 2020;**7**:547–60. doi:10.1016/S2215-0366(20)30168-1

6 Bashshur R, Doarn CR, Frenk JM, *et al.* Telemedicine and the COVID-19 Pandemic, Lessons for the Future. *Telemed e-Health* 2020;**26**:571–3. doi:10.1089/tmj.2020.29040.rb

7 Chen S, Jones PB, Underwood BR, *et al.* The early impact of COVID-19 on mental health and community physical health services and their patients' mortality in Cambridgeshire and Peterborough, UK. *J Psychiatr Res* 2020;**131**:244–54. doi:10.1016/j.jpsychires.2020.09.020

8 Bokolo Anthony Jnr. Use of Telemedicine and Virtual Care for Remote Treatment in Response to COVID-19 Pandemic. *J Med Syst* 2020;**44**:1–9. doi:10.1007/s10916-020-01596-5

9 Iacobucci G. Covid-19: New restrictions to curb rising infection rate are set throughout UK. *BMJ* 2020;**370**:m3712. doi:10.1136/bmj.m3712

10 Xu S, Li Y. Beware of the second wave of COVID-19. Lancet. 2020;**395**:1321–2. doi:10.1016/S0140-6736(20)30845-X

11 Panovska-Griffiths J, Kerr CC, Stuart RM, *et al.* Determining the optimal strategy for reopening schools, the impact of test and trace interventions, and the risk of occurrence of a second COVID-19 epidemic wave in the UK: a modelling study. *Lancet Child Adolesc Heal* Published Online First: August 2020. doi:10.1016/S2352-4642(20)30250-9

12 Perera G, Broadbent M, Callard F, *et al.* Cohort profile of the South London and Maudsley NHS Foundation Trust Biomedical Research Centre (SLaM BRC) Case Register: current status and recent enhancement of an Electronic Mental Health Record-derived data resource. *BMJ Open* 2016;**6**. doi:10.1136/bmjopen-2015-008721

13 Jackson RG, Patel R, Jayatilleke N, *et al.* Natural language processing to extract symptoms of severe mental illness from clinical text: the Clinical Record Interactive Search Comprehensive Data Extraction (CRIS-CODE) project. *BMJ Open* 2017;**7**.http://bmjopen.bmj.com/content/7/1/e012012.abstract

14 Kadra G, Stewart R, Shetty H, *et al.* Antipsychotic polypharmacy prescribing and risk of hospital readmission. *Psychopharmacology (Berl)* 2018;**235**:281–9. doi:10.1007/s00213-017-4767-6

15 Kesserwani J, Kadra G, Downs J, *et al.* Risk of readmission in patients with schizophrenia and schizoaffective disorder newly prescribed clozapine. *J Psychopharmacol* 2019;**33**:449–58. doi:10.1177/0269881118817387

16 Soysal P, Perera G, Isik AT, *et al.* The relationship between polypharmacy and trajectories of cognitive decline in people with dementia: A large representative cohort study. *Exp Gerontol* 2019;**120**:62–7. doi:10.1016/j.exger.2019.02.019

17 Jean-Baptiste R, Toubiana L, Le Mignot L, *et al.* A Web-based GIS for health care decision-support. *AMIA Annu Symp Proc* 2005;**2005**:365–9.

18 Ghetian CB, Parrott R, Volkman JE, *et al.* Cancer registry policies in the United States and geographic information systems applications in comprehensive cancer control. *Health Policy (New York)* 2008;**87**:185–93. doi:10.1016/j.healthpol.2007.12.007

For peer review only - http://bmjopen.bmj.com/site/about/guidelines.xhtml

19 Ghebreyesus TA. WHO Director-General's opening remarks at the media briefing on COVID-19 - 11 March 2020. 2020.https://www.who.int/dg/speeches/detail/who-director-general-s-opening-remarks-at-the-media-briefing-on-covid-19---11-march-2020

20 Stewart R, Soremekun M, Perera G, *et al.* The South London and Maudsley NHS Foundation Trust Biomedical Research Centre (SLAM BRC) case register: development and descriptive data. *BMC Psychiatry* 2009;**9**:51.http://www.biomedcentral.com/1471-244X/9/51

21 South London and Maudsley NHS Foundation Trust. Clinical Record Interactive Search (CRIS). https://slam.nhs.uk/quality-and-research/clinical-record-interactive-search-cris/

22 Downs J, Dean H, Lechler S, *et al.* Negative Symptoms in Early-Onset Psychosis and Their Association With Antipsychotic Treatment Failure. *Schizophr Bull* 2019;**45**:69–79. doi:10.1093/schbul/sbx197

23 Downs J, Hotopf M, Ford T, *et al.* Clinical predictors of antipsychotic use in children and adolescents with autism spectrum disorders: a historical open cohort study using electronic health records. *Eur Child Adolesc Psychiatry* 2016;**25**:649–58. doi:10.1007/s00787-015-0780-7

24 CRIS Natural Language Processing Applications Library. https://www.maudsleybrc.nihr.ac.uk/facilities/clinical-record-interactive-search-cris/cris-natural-language-processing/

25 Morrison P, Taylor DM, McGuire P. *The Maudsley Guidelines on Advanced Prescribing in Psychosis*. Wiley 2019. https://books.google.co.uk/books?id=88C_DwAAQBAJ

26 Stokel-Walker C. Why telemedicine is here to stay. *BMJ* 2020;**371**:m3603. doi:10.1136/bmj.m3603

27 Stewart R, Martin E, Broadbent M. Mental health service activity during COVID-19 lockdown: South London and Maudsley data on working age community and home treatment team services and mortality from February to mid-May 2020. *medRxiv* 2020;:2020.06.13.20130419. doi:10.1101/2020.06.13.20130419

28 Scott Kruse C, Karem P, Shifflett K, *et al.* Evaluating barriers to adopting telemedicine worldwide: A systematic review. *J Telemed Telecare* 2016;**24**:4–12. doi:10.1177/1357633X16674087

29 Stewart R, Martin E, Bakolis I, *et al.* Comparison of mental health service activity before and shortly after UK social distancing responses to the COVID-19 pandemic: February-March 2020. *medRxiv* 2020;:2020.09.26.20202150. doi:10.1101/2020.09.26.20202150

30 Clozapine drug monitoring during COVID-19 for stable adult patients – SPS - Specialist Pharmacy Service – The first stop for professional medicines advice. https://www.sps.nhs.uk/articles/clozapine-drug-monitoring-in-primary-care-during-covid-19-for-stable-patients/

31 Alsdorf R, Wyszynski DF. Teratogenicity of sodium valproate. Expert Opin. Drug Saf. 2005;**4**:345–53. doi:10.1517/14740338.4.2.345

32 Taylor D, Barnes TRE, Young AH. *The Maudsley Prescribing Guidelines in Psychiatry (Thirteenth edition)*. 13th ed. 2018.

33 Voineskos D, Daskalakis ZJ, Blumberger DM. Management of Treatment-Resistant Depression: Challenges and Strategies. *Neuropsychiatr Dis Treat* 2020;**16**:221–34. doi:10.2147/NDT.S198774

34 Rybakowski JK. Challenging the Negative Perception of Lithium and Optimizing Its Long-Term Administration . Front. Mol. Neurosci. . 2018;**11**:349.https://www.frontiersin.org/article/10.3389/fnmol.2018.00349

35 Choi NG, DiNitto DM. The Digital Divide Among Low-Income Homebound Older Adults: Internet Use Patterns, eHealth Literacy, and Attitudes Toward Computer/Internet Use. *J Med Internet Res* 2013;**15**:e93. doi:10.2196/jmir.2645

36 Krzystanek M, Krysta K, Skałacka K. Treatment Compliance in the Long-Term Paranoid Schizophrenia Telemedicine Study. *J Technol Behav Sci* 2017;**2**:84–7. doi:10.1007/s41347-017-0016-4

37 Wang Q, Xu R, Volkow ND. Increased risk of COVID-19 infection and mortality in people with mental disorders: analysis from electronic health records in the United States. *World Psychiatry* 2020;**n/a**. doi:10.1002/wps.20806

For peer review only - http://bmjopen.bmj.com/site/about/guidelines.xhtml

38 Balikci A, Erdem M, Zincir S, *et al.* Adherence with Outpatient Appointments and Medication: A Two-Year Prospective Study of Patients with Schizophrenia. *Klin Psikofarmakol Bülteni-Bulletin Clin Psychopharmacol* 2013;**23**:57–64. doi:10.5455/bcp.20121130085931

39 Almond S, Knapp M, Francois C, *et al.* Relapse in schizophrenia: Costs, clinical outcomes and quality of life. *Br J Psychiatry* 2004;**184**:346–51. doi:10.1192/bjp.184.4.346

40 Mitchell AJ, Selmes T. Why don't patients attend their appointments? Maintaining engagement with psychiatric services. *Adv Psychiatr Treat* 2007;**13**:423–34. doi:10.1192/apt.bp.106.003202

41 Magnes RM. Out-patient appointments: A necessary evil? A literature review and survey of patient attendance records. Psychiatr. Bull. 2008;**32**:458–60. doi:10.1192/pb.bp.108.020065

42 Urness D, Wass M, Gordon A, *et al.* Client acceptability and quality of life - telepsychiatry compared to in-person consultation. *J Telemed Telecare* Published Online First: 2006. doi:10.1258/135763306777889028

43 Berry N, Lobban F, Emsley DcPR, *et al.* Acceptability of interventions delivered online and through mobile phones for people who experience severe mental health problems: A systematic review. J. Med. Internet Res. 2016. doi:10.2196/jmir.5250

44 World Health Organization. *Global diffusion of eHealth: Making universal health coverage achievable*. 2016. https://www.who.int/goe/publications/global_diffusion/en/

For peer review only

For peer review only - http://bmjopen.bmj.com/site/about/guidelines.xhtml

Figure 1. Number of patients registered with SLaM services each week by age group

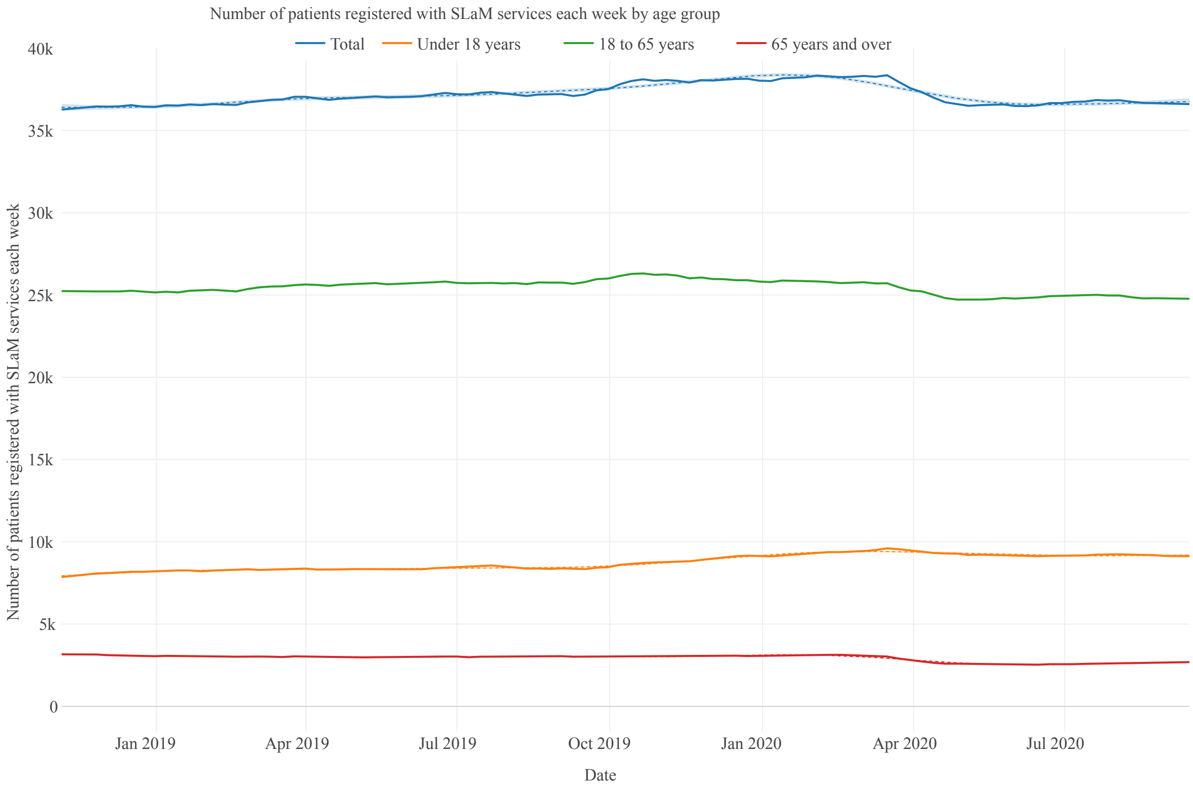

For peer review only

For peer review only - http://bmjopen.bmj.com/site/about/guidelines.xhtml

Figure 2: Total number of clinical events each week

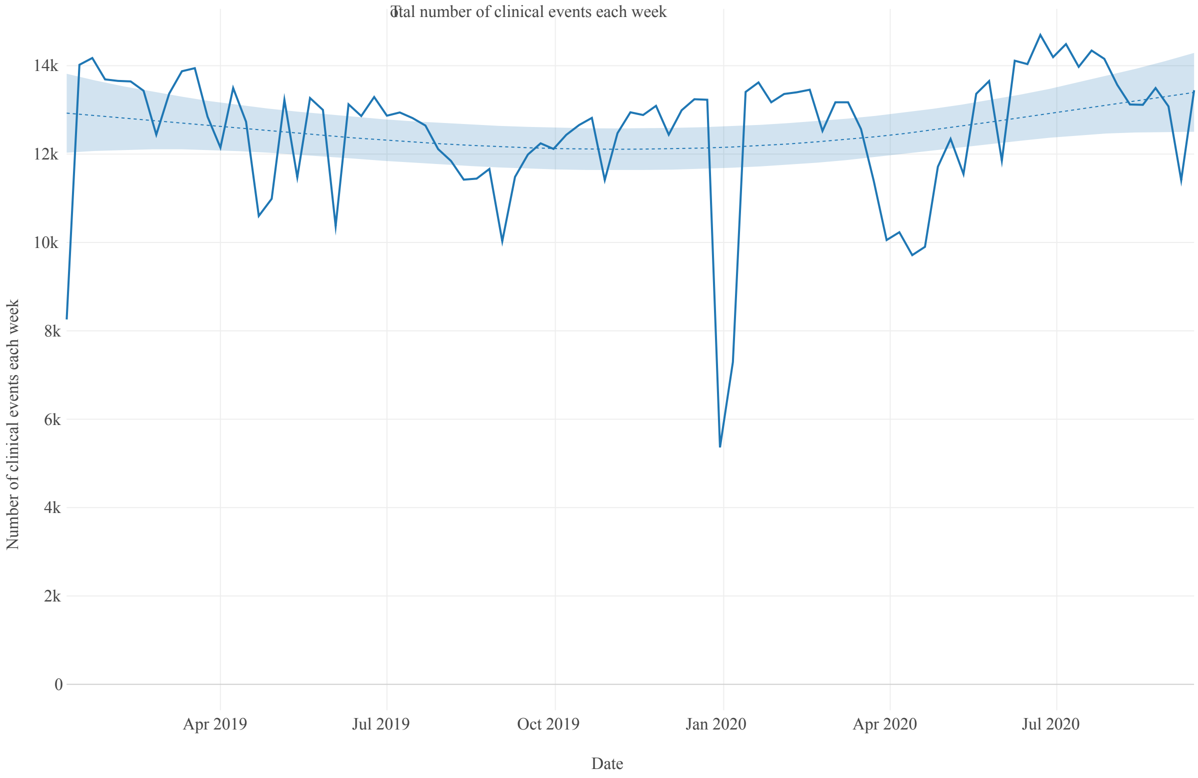

review only

For peer review only - http://bmjopen.bmj.com/site/about/guidelines.xhtml

Figure 3: Number of in-person, remote and did-not-attend (DNA) clinical events each week

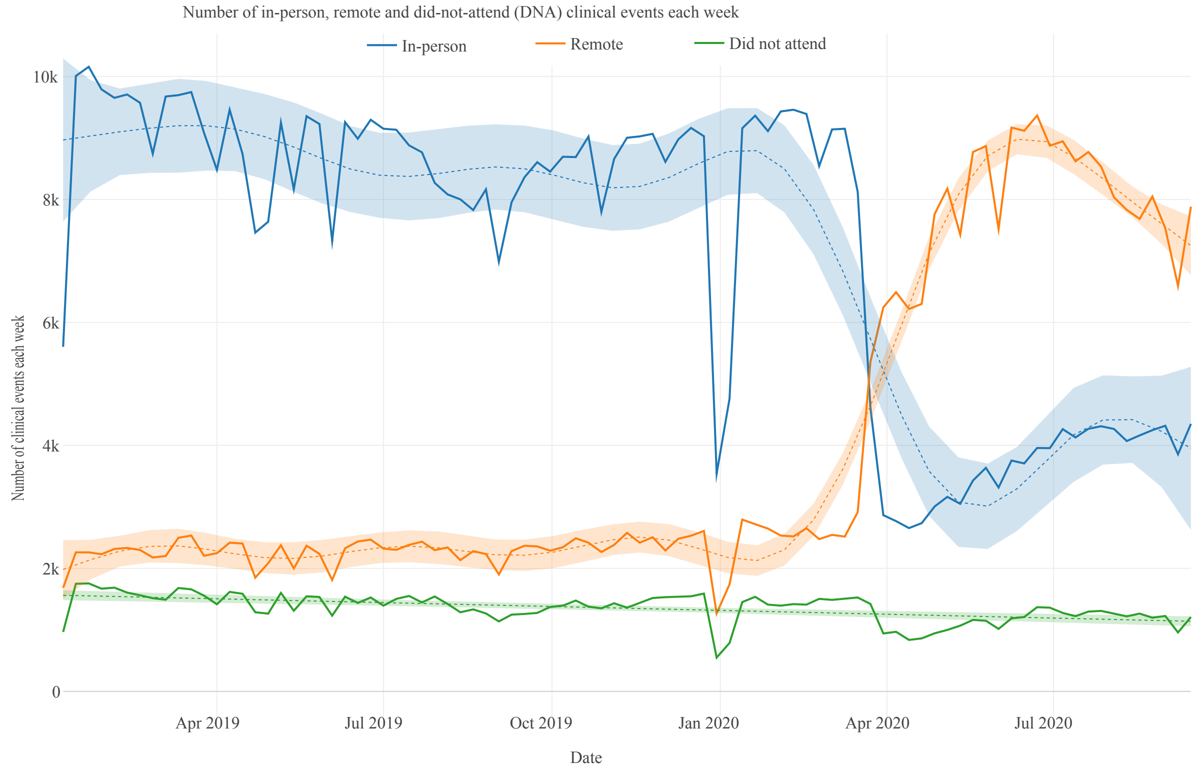

Figure 4: Total number of clinical events each week by age group

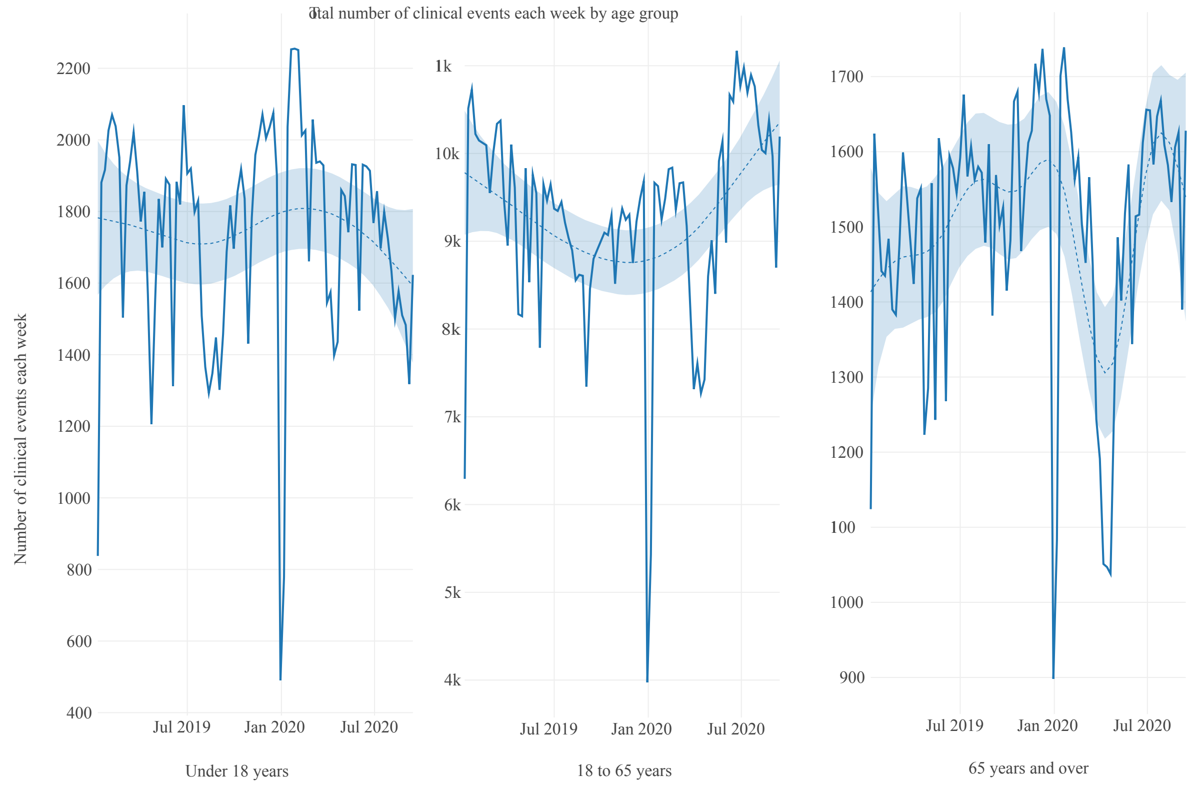

Note: The y-axes presented here do not start at zero and are not standardised between charts.

For peer review only

For peer review only - http://bmjopen.bmj.com/site/about/guidelines.xhtml

Figure 5: Modes of clinical contact in different age groups

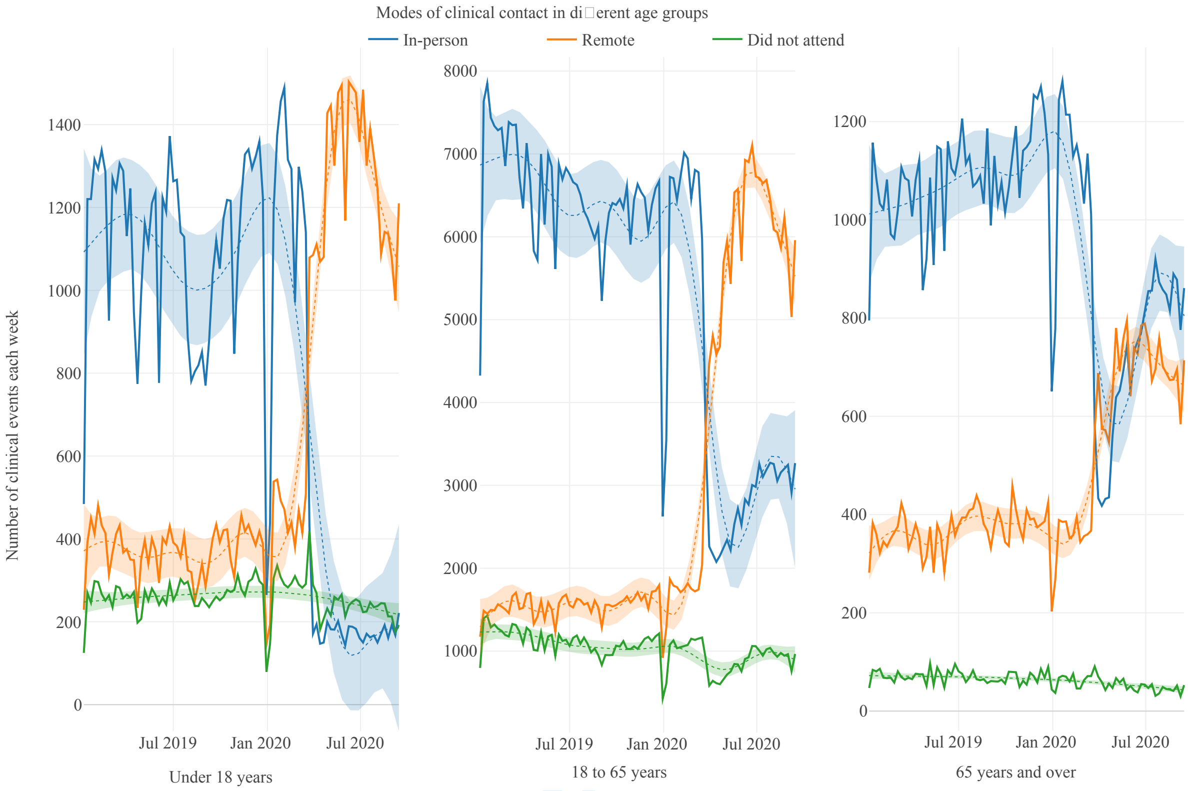

Note: The y-axes presented here do not all start at zero and are not standardised between charts.

For peer review only - http://bmjopen.bmj.com/site/about/guidelines.xhtml

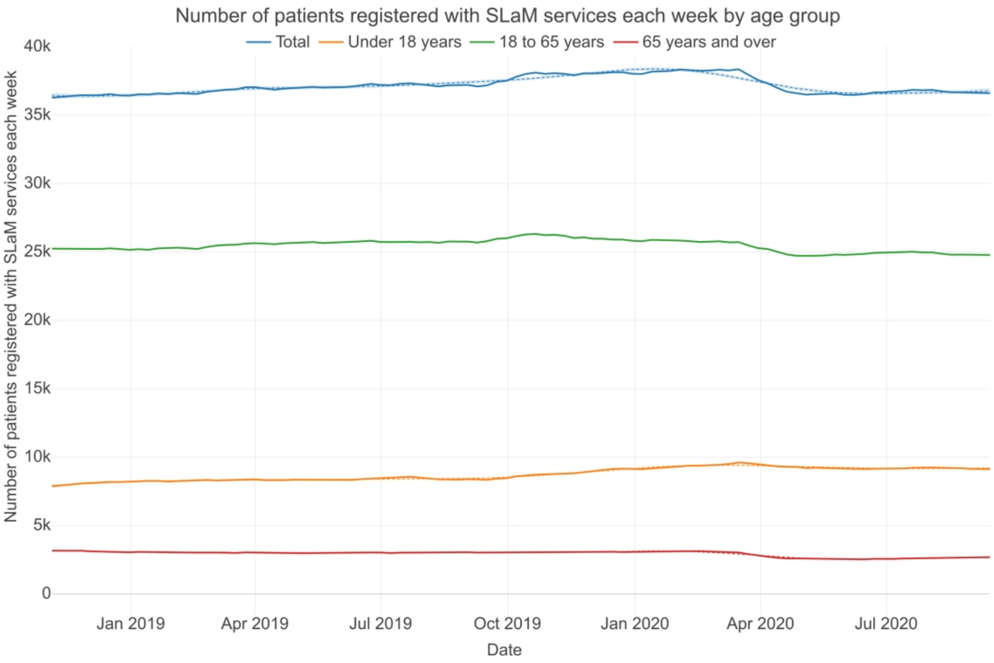

Figure 1. Number of patients registered with SLaM services each week by age group.

203x135mm (300 x 300 DPI)

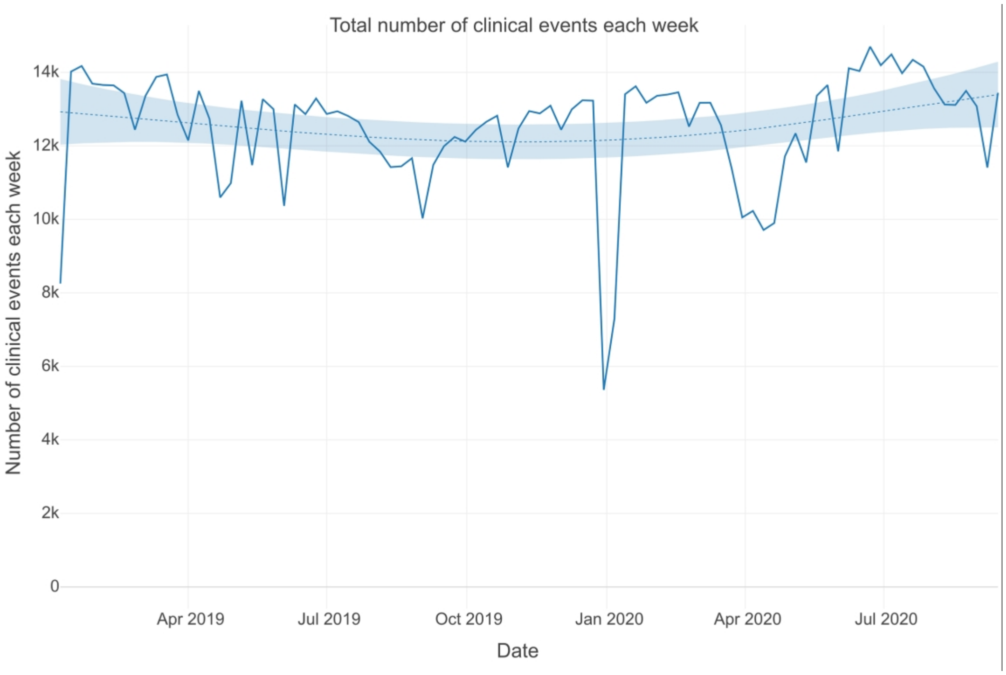

Figure 2: Total number of clinical events each week.

203x135mm (300 x 300 DPI)

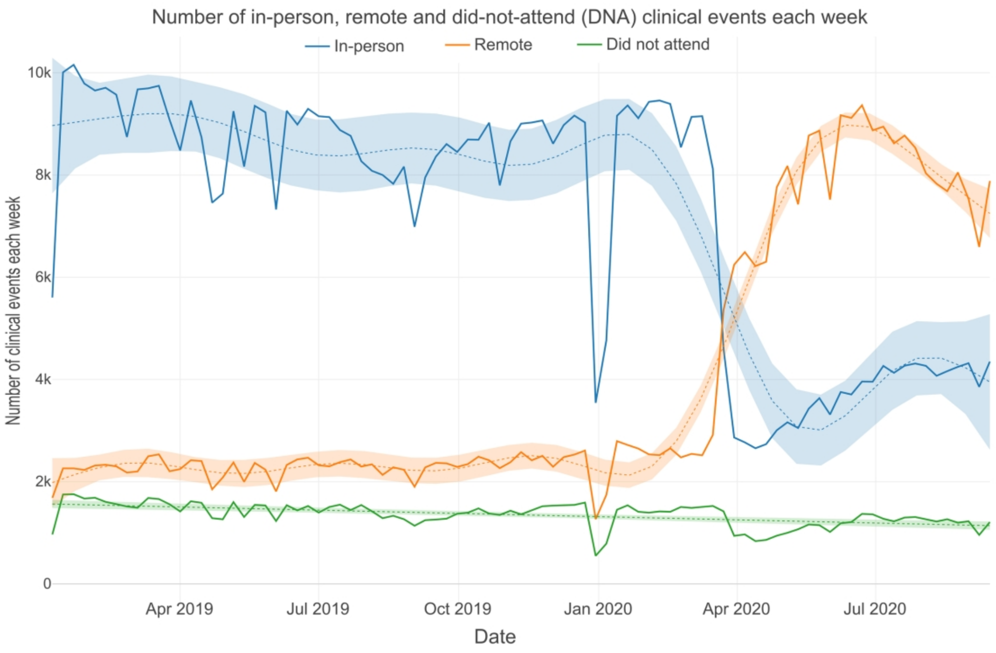

Figure 3: Number of in-person, remote and did-not-attend (DNA) clinical events each week.

203x135mm (300 x 300 DPI)

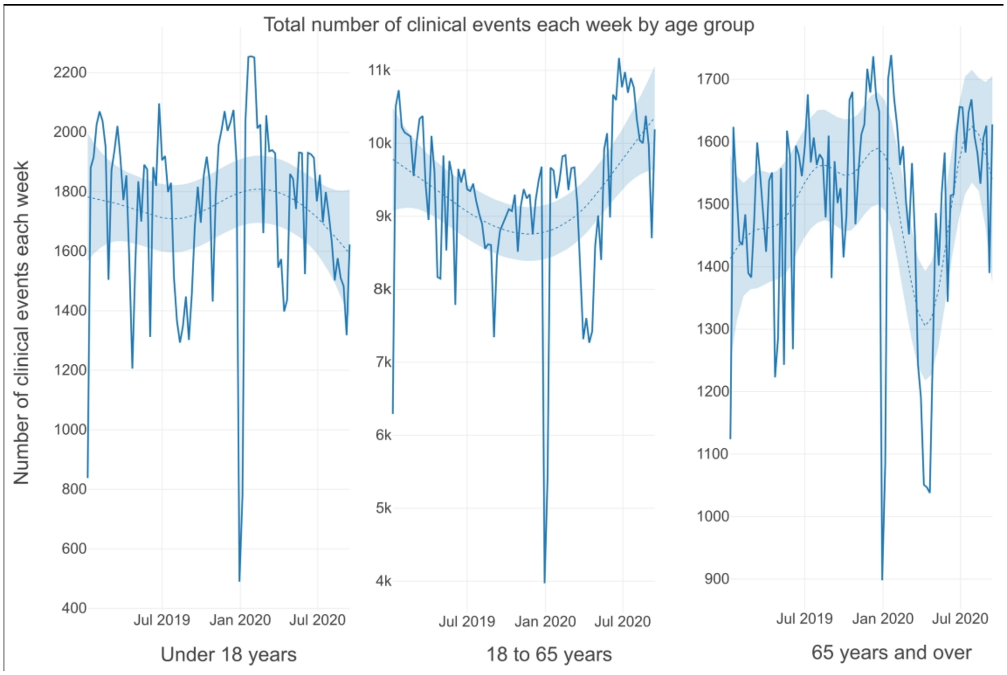

Figure 4: Total number of clinical events each week by age group. Note: The y-axes presented here do not start at zero and are not standardised between charts.

203x135mm (300 x 300 DPI)

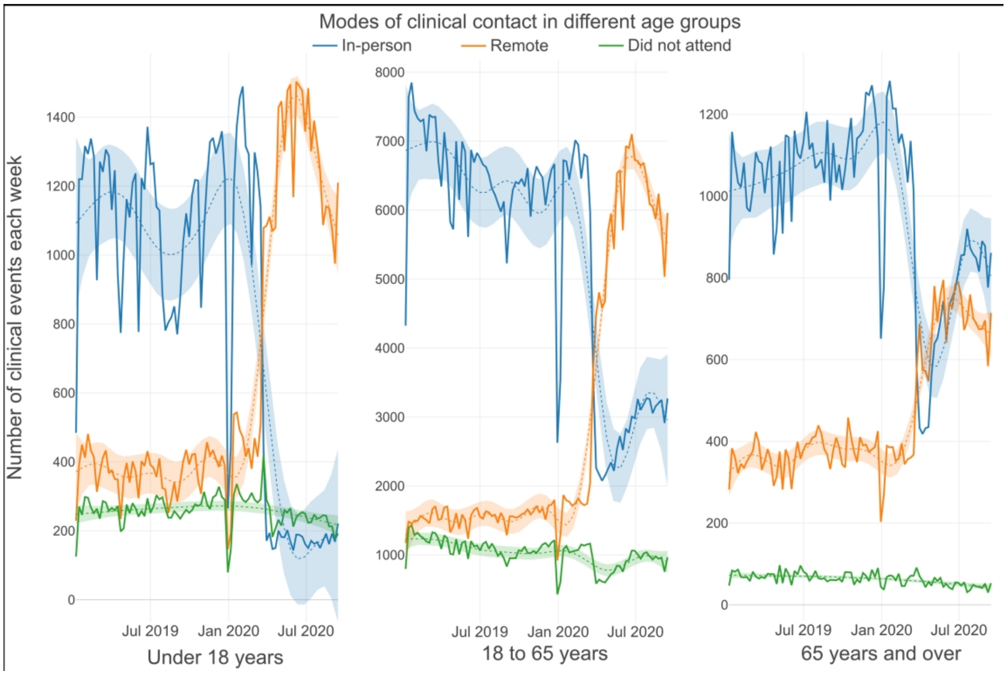

Figure 5: Modes of clinical contact in different age groups. Note: The y-axes presented here do not all start at zero and are not standardised between charts.

203x135mm (300 x 300 DPI)

Impact of the COVID-19 pandemic on remote mental healthcare and prescribing in psychiatry

Supplementary Material

Table of Contents

Interpretation of the interactive dashboard 1

Supplementary Figure 1. Number of patients registered with SLaM services each week by gender 2

Supplementary Figure 2. Total number of clinical events each week by gender.. 2

Supplementary Figure 3. Number of in-person, remote and non-attended clinical events each week by gender 3

Supplementary Figure 4: Number of antipsychotics recorded each week 3

Supplementary Figure 5. Number of antipsychotics recorded each week by age group 4

Supplementary Figure 6. Number of antipsychotics recorded each week by gender 4

Supplementary Figure 7: Number of mood stabilisers recorded each week... 5

Supplementary Figure 8. Number of mood stabilisers recorded each week by gender................................. 5

Supplementary Figure 9. Number of mood stabilisers recorded each week by age group 6

Interpretation of the interactive dashboard

The data visualisation dashboard at http://rpatel.co.uk/TelepsychiatryDashboard has a number of interactive capabilities:

1. Click and drag along the axes to scroll to different values
2. To zoom in on a time period, click and drag horizontally in the chart space to highlight the time period of interest
3. To zoom in on a data series, click and drag vertically in the chart space to highlight the data series of interest
4. Double click to exit zoom mode and revert to default
5. Click on a legend item to hide that category of data from the chart temporarily

More information can be found at *https://exploratory.io/note/2ac8ae888097/0937480844541865*.

For peer review only

Supplementary Figure 1. Number of patients registered with SLaM services each week by gender

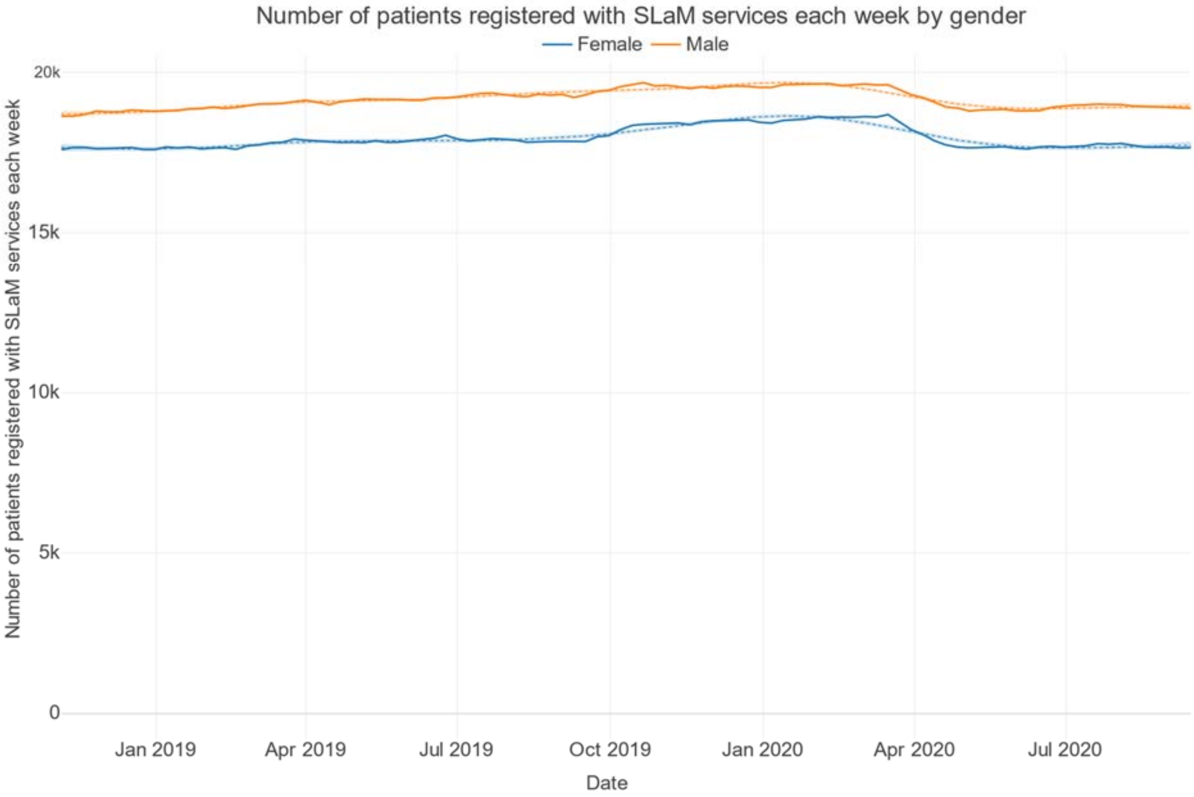

Supplementary Figure 2. Total number of clinical events each week by gender

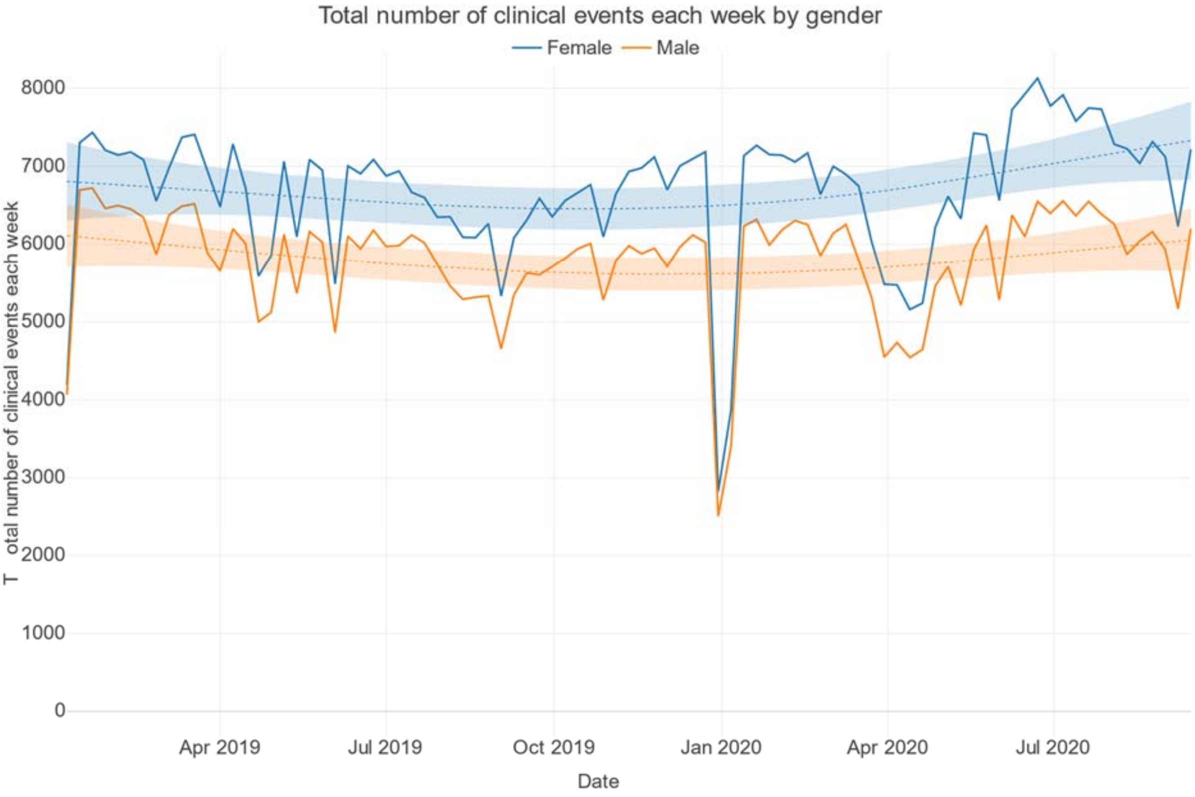

Supplementary Figure 3. Number of in-person, remote and non-attended clinical events each week by gender

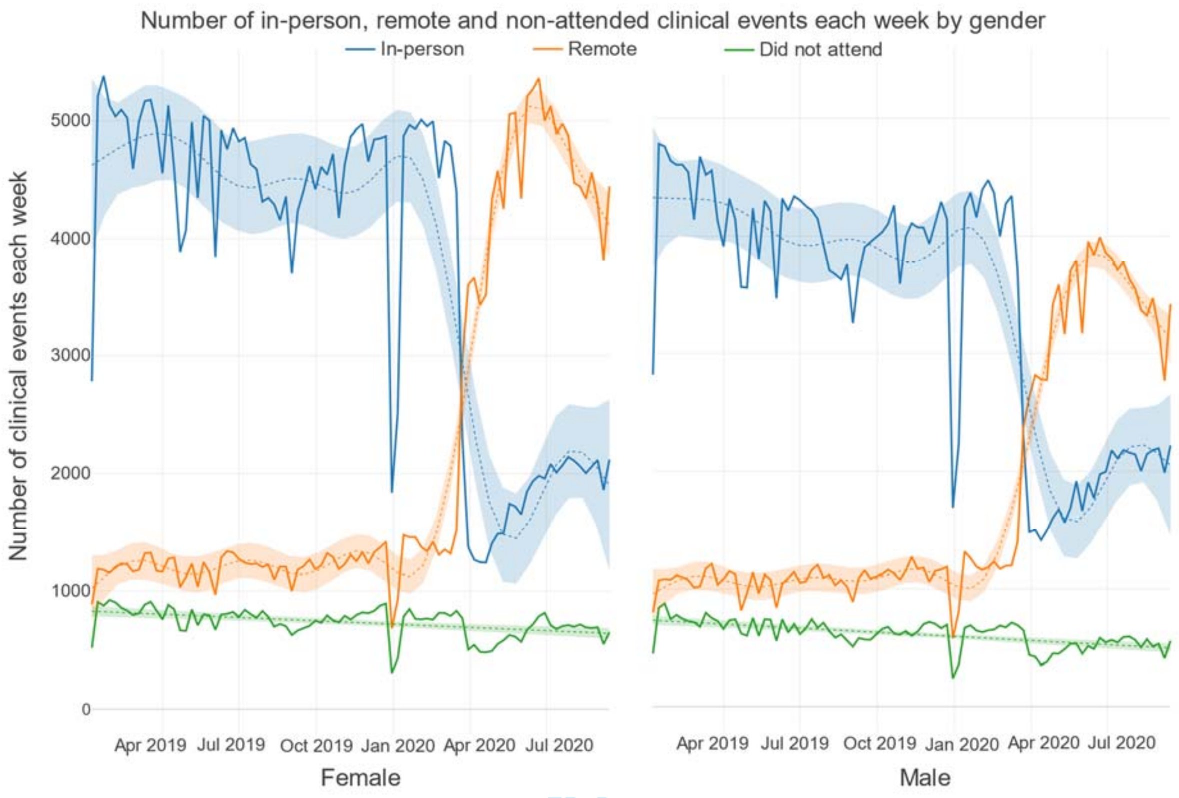

Supplementary Figure 4: Number of antipsychotics recorded each week

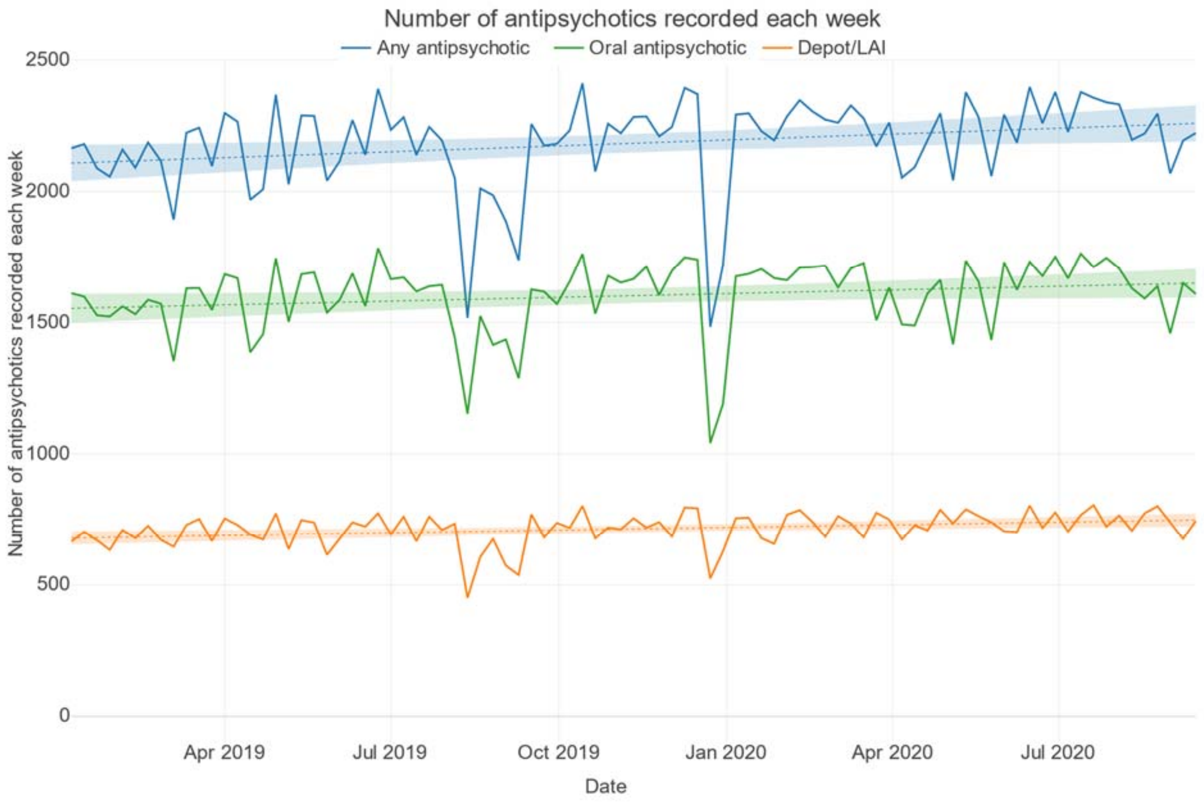

Supplementary Figure 5. Number of antipsychotics recorded each week by age group

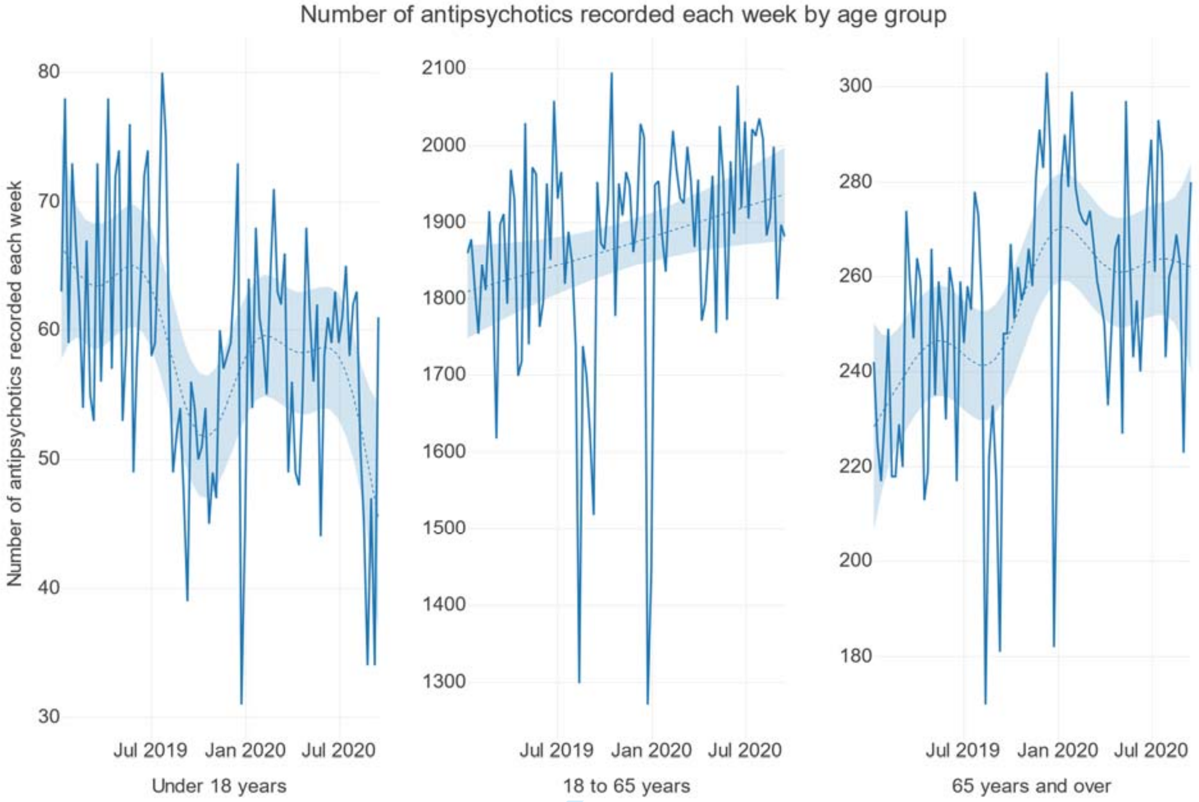

Note: The y-axes presented here do not all start at zero and are not standardised between charts.

Supplementary Figure 6. Number of antipsychotics recorded each week by gender

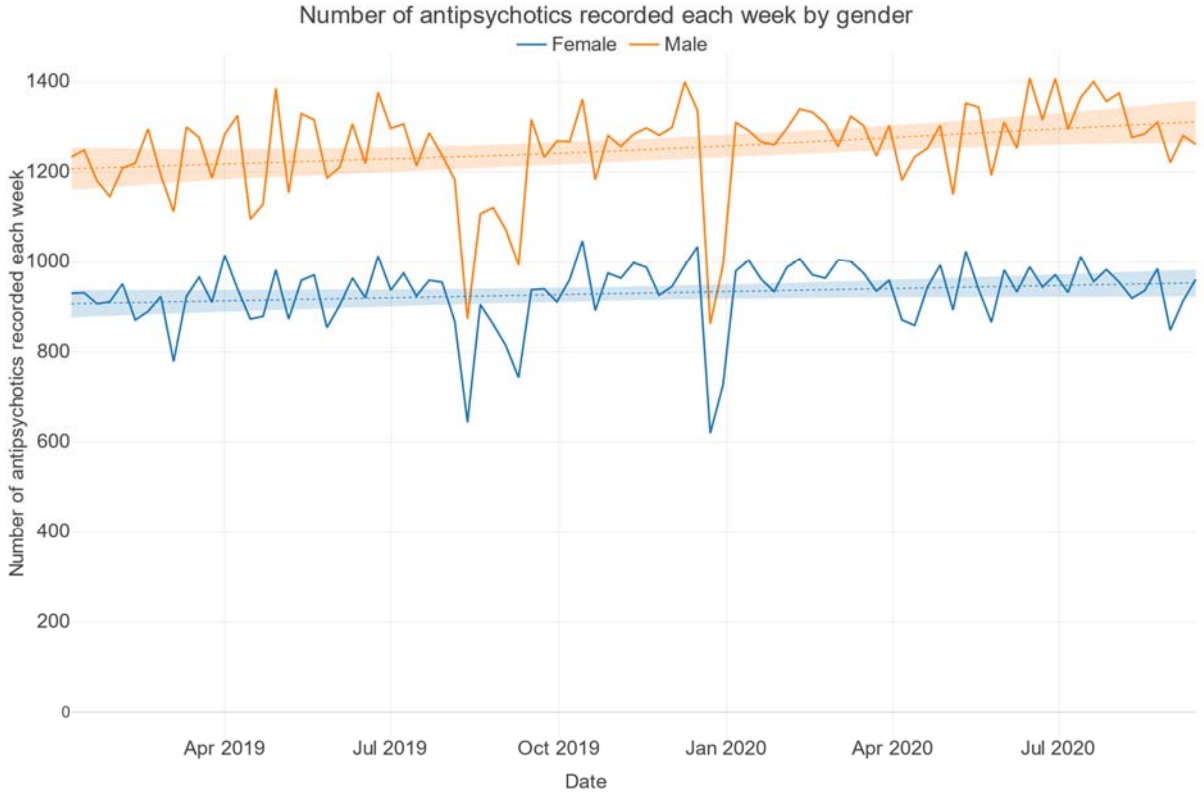

For peer review only - http://bmjopen.bmj.com/site/about/guidelines.xhtml

Supplementary Figure 7: Number of mood stabilisers recorded each week

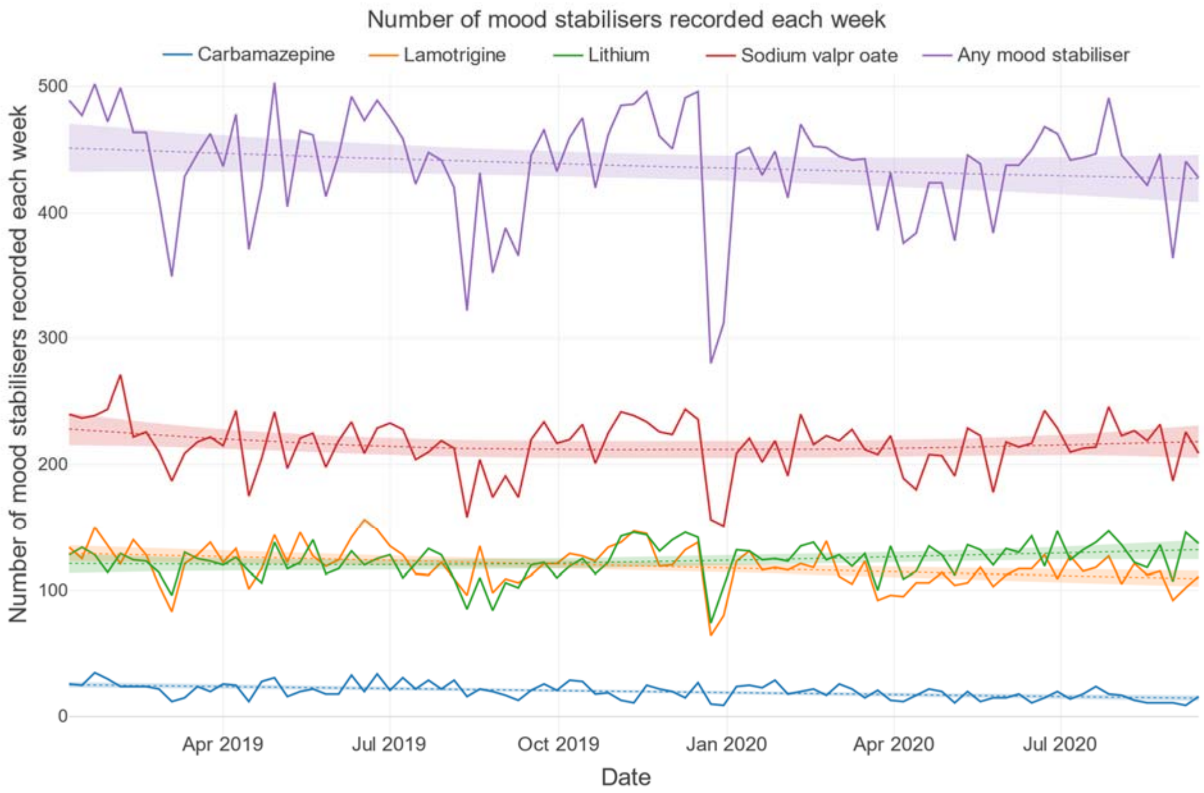

Supplementary Figure 8. Number of mood stabilisers recorded each week by gender

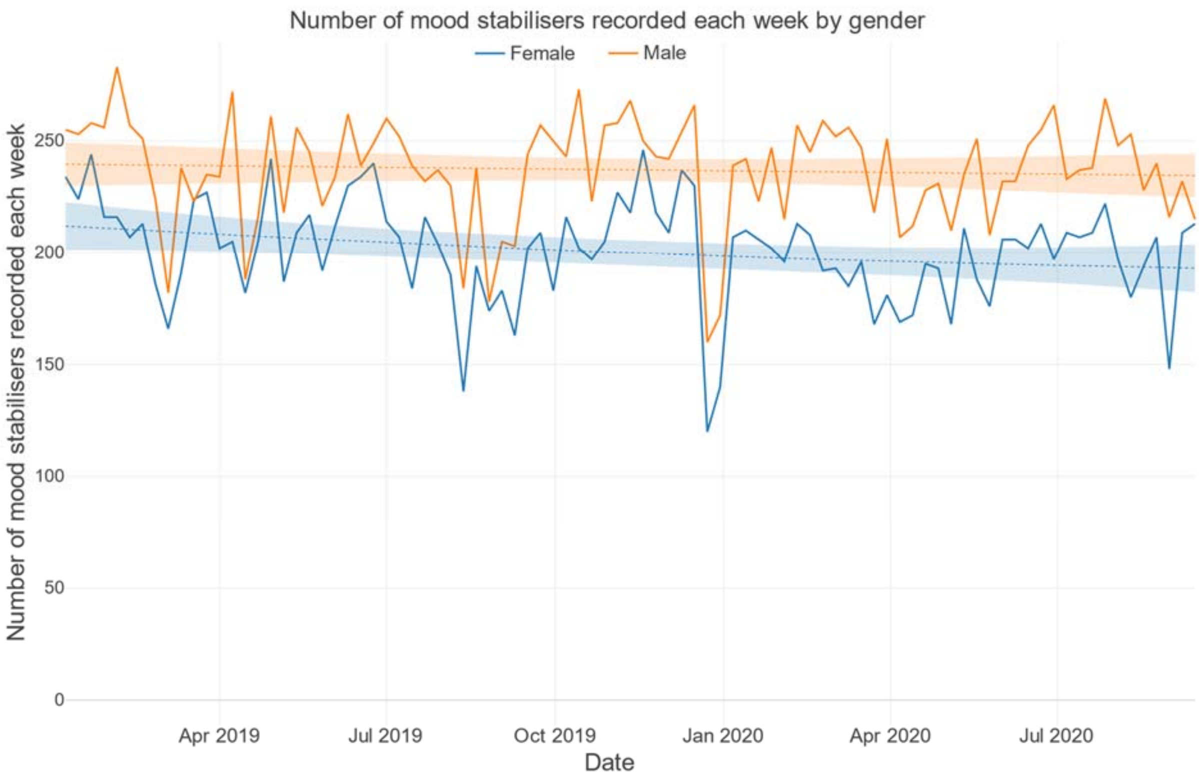

Supplementary Figure 9. Number of mood stabilisers recorded each week by age group

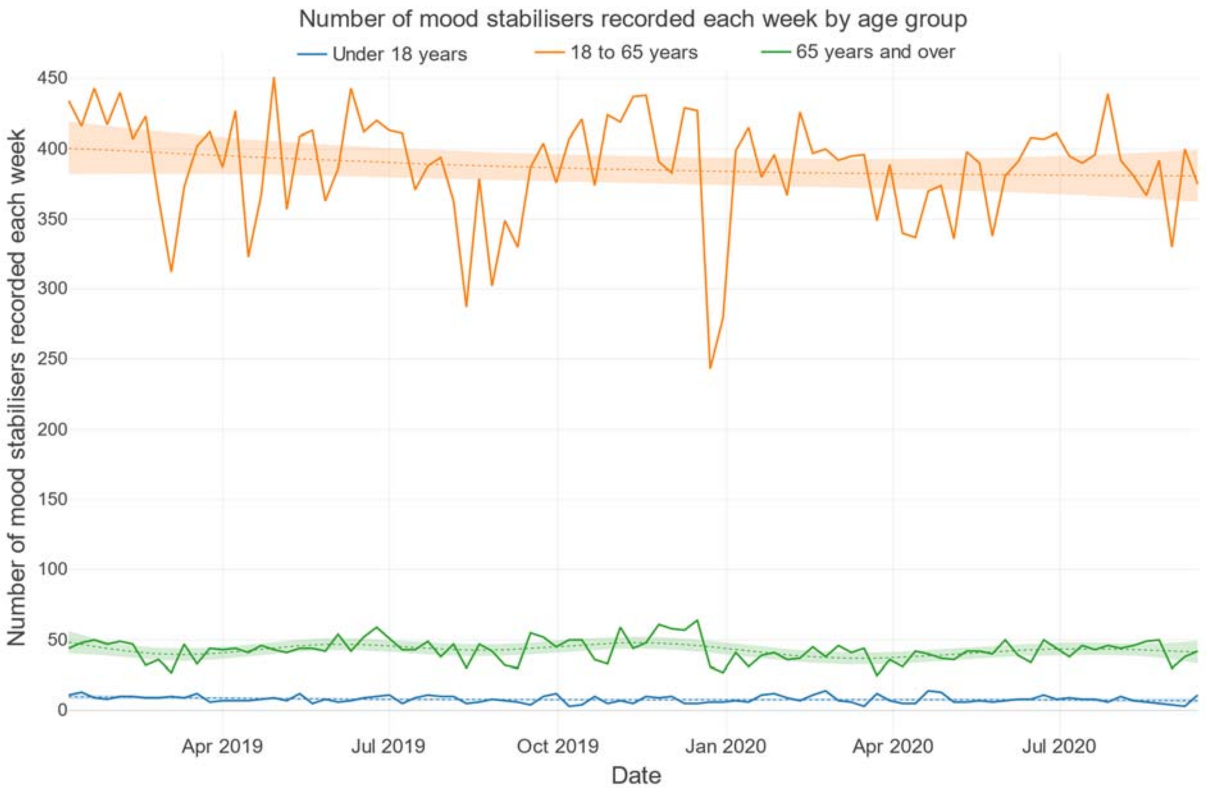

review only

Supplementary Table 1

Index period vs March 2020 and April 2020

Variable	Index period: 4 March 2019 to 1 March 2020	March 2020: β coefficient	p	95% CI Lower limit	95% CI Upper limit	April 2020: β coefficient	p	95% CI Lower limit	95% CI Upper limit
Number of patients enrolled in mental health services	**Mean (95% Confidence Interval)**	**β coefficient: mean number of patients**	**p**	**Lower limit**	**Upper limit**	**β coefficient: number of patients**	**p**	**Lower limit**	**Upper limit**
**Total**	37563.2 (37419.8 to 37706.6)	711.5	0.009	200.6	1222.5	-333.2	0.211	-849.6	183.2
**Female**	18111.4 (18026.3 to 18196.4)	470.1	0.004	167.3	773.0	-149.9	0.341	-455.8	156.1
**Male**	19355.6 (19296.5 to 19414.7)	221.9	0.044	11.0	432.8	-226.8	0.042	-440.5	-13.2
**Under 18 years**	8648.3 (8546.1 to 8750.4)	877.7	<0.001	514.7	1240.7	723.2	<0.001	360.2	1086.3
**18 to 64 years**	25810.7 (24945.4 to 25010.6)	-151.5	0.140	-349.7	46.8	-738.0	<0.001	-940.0	-535.9
**65 years and older**	3041.4 (3029.8 to 3053.0)	-21.2	0.365	-66.6	24.2	-349.4	<0.001	-396.2	-302.6
Number of contacts with mental health professionals	**Mean (95% Confidence Interval)**	**β coefficient: mean number of weekly contacts**	**p**	**Lower limit**	**Upper limit**	**β coefficient: number of weekly contacts**	**p**	**Lower limit**	**Upper limit**
**In person**	8581.8 (8325.7 to 8838.0)	-847.6	0.174	-2052.7	357.6	-5829.6	<0.001	-6919.5	-4739.6
**Remote**	2328.2 (2267.3 to 2389.2)	948.3	<0.001	546.5	1350.1	3338.5	<0.001	3074.4	3602.7
**Did not attend (DNA)**	1411.9 (1366.8 to 1456.9)	66.4	0.501	-125.7	258.5	-525.4	<0.001	-717.5	-333.2
Number of antipsychotic mentions	**Mean (95% Confidence Interval)**	**β coefficient: mean number of weekly antipsychotics**	**p**	**Lower limit**	**Upper limit**	**β coefficient: number of weekly antipsychotics**	**p**	**Lower limit**	**Upper limit**
**Any antipsychotic**	2056.0 (2020.1 to 2091.3)	89.8	0.256	-63.4	242.9	10.3	0.897	-144.3	164.9
**Any depot**	666.9 (655.0 to 678.7)	30.6	0.252	-21.2	82.5	20.1	0.443	-30.9	71.2
**Any oral**	1495.2 (1465.7 to 1524.6)	55.3	0.398	-72.0	182.7	-13.9	0.830	-140.6	112.8
Number of individual antipsychotic mentions	**Mean (95% Confidence Interval)**	**β coefficient: mean number of weekly antipsychotics**	**p**	**Lower limit**	**Upper limit**	**β coefficient: number of weekly antipsychotics**	**p**	**Lower limit**	**Upper limit**
**Amisulpiride**	90.3 (87.4 to 93.2)	1.2	0.855	-11.8	14.2	-1.5	0.813	-14.2	11.2
**Aripiprazole**	336.6 (327.3 to 346.0)	34.1	0.102	-6.0	74.3	-27.9	0.180	-68.1	12.4
**Clozapine**	348.3 (340.0 to 356.7)	14.7	0.445	-22.7	52.0	79.2	<0.001	42.5	115.8
**Haloperidol**	49.0 (47.2 to 50.7)	7.0	0.069	-0.4	14.5	7.3	0.058	-0.1	14.7
**Lurasidone**	38.0 (36.3 to 39.7)	5.5	0.152	-1.9	12.8	-0.3	0.939	-7.6	7.0
**Olanzapine**	424.5 (413.4 to 435.6)	11.0	0.589	-28.6	50.6	-38.8	0.063	-78.8	1.3
**Quetiapine**	189.0 (183.9 to 194.1)	4.3	0.707	-17.8	26.3	-11.3	0.325	-33.4	10.9
**Risperidone**	238.2 (232.8 to 243.7)	-11.0	0.364	-34.4	12.5	-32.0	0.009	-55.2	-8.7
**Sulpiride**	7.4 (6.7 to 8.0)	-2.3	0.092	-5.0	0.3	0.4	0.766	-2.2	3.1
**Zuclopenthixol**	9.8 (9.1 to 10.6)	-2.1	0.209	-5.3	1.1	0.9	0.573	-2.3	4.1
Number of antipsychotic depot mentions	**Mean (95% Confidence Interval)**	**β coefficient: mean number of weekly depots**	**p**	**Lower limit**	**Upper limit**	**β coefficient: number of weekly depots**	**p**	**Lower limit**	**Upper limit**
**Aripiprazole depot**	115.6 (111.8 to 119.4)	31.9	<0.001	15.6	48.1	17.4	0.040	1.2	33.5
**Paliperidone depot**	214.4 (207.9 to 221.0)	8.3	0.588	-21.6	38.2	0.3	0.741	-1.3	1.9
**Haloperidol depot**	69.6 (67.5 to 71.7)	-4.8	0.303	-14.0	4.3	7.3	0.058	-0.1	14.7
**Risperidone depot**	38.4 (36.8 to 39.9)	-7.1	0.038	-13.6	-0.5	-6.3	0.060	-12.8	0.1
**Zuclopenthixol depot**	111.2 (109.0 to 113.4)	4.5	0.373	-5.3	14.4	-6.2	0.223	-16.1	3.7
Number of mood stabiliser mentions	**Mean (95% Confidence Interval)**	**β coefficient: mean number of weekly mood stabilisers**	**p**	**Lower limit**	**Upper limit**	**β coefficient: number of weekly mood stabilisers**	**p**	**Lower limit**	**Upper limit**
**Any mood stabiliser**	405.1 (395.7 to 414.5)	-17.8	0.391	-58.2	22.6	-31.3	0.137	-72.1	9.4
Number of individual mood stabiliser mentions	**Mean (95% Confidence Interval)**	**β coefficient: mean number of weekly mood stabilisers**	**p**	**Lower limit**	**Upper limit**	**β coefficient: number of weekly mood stabilisers**	**p**	**Lower limit**	**Upper limit**
**Carbamazepine**	20.8 (19.4 to 22.1)	-1.0	0.729	-6.9	4.8	-6.3	0.041	-12.2	-0.4
**Lamotrigine**	116.7 (113.1 to 120.4)	-14.2	0.084	-30.1	1.6	-17.5	0.032	-33.1	-1.9
**Lithium**	112.4 (109.3 to 115.4)	-1.8	0.786	-15.1	11.4	2.9	0.673	-10.5	16.3
**Valproate**	191.9 (187.3 to 196.4)	-5.4	0.588	-24.7	13.9	-15.4	0.140	-35.5	4.8

Index period vs May, June and July 2020

Variable	May 2020: β coefficient	p	95% CI Lower limit	95% CI Upper limit	June 2020: β coefficient	p	95% CI Lower limit	95% CI Upper limit	July 2020: β coefficient	p	95% CI Lower limit	95% CI Upper limit
Number of patients enrolled in mental health services	**β coefficient: number of patients**	**p**	**Lower limit**	**Upper limit**	**β coefficient: number of patients**	**p**	**Lower limit**	**Upper limit**	**β coefficient: number of patients**	**p**	**Lower limit**	**Upper limit**
**Total**	-918.2	<0.001	-1373.5	-462.9	-916.2	0.001	-1425.7	-406.8	-688.4	0.005	-1143.9	-232.9
**Female**	-453.2	0.002	-723.1	-183.3	-461.1	0.004	-763.1	-159.2	-394.6	0.006	-664.7	-124.5
**Male**	-515.6	<0.001	-703.5	-327.7	-520.8	<0.001	-731.2	-310.5	-364.0	<0.001	-551.8	-176.2
**Under 18 years**	570.7	0.001	246.4	895.1	485.5	0.011	122.9	848.1	547.1	0.002	222.8	871.5
**18 to 64 years**	-1076.9	<0.001	-1252.3	-901.6	-967.0	<0.001	-1163.3	-770.6	-832.7	<0.001	-1007.8	-657.6
**65 years and older**	-456.6	<0.001	-493.5	-419.7	-495.4	<0.001	-536.8	-454.0	-460.8	<0.001	-498.0	-423.6
Number of contacts with mental health professionals	**β coefficient: number of weekly contacts**	**p**	**Lower limit**	**Upper limit**	**β coefficient: number of weekly contacts**	**p**	**Lower limit**	**Upper limit**	**β coefficient: number of weekly contacts**	**p**	**Lower limit**	**Upper limit**
**In person**	-5335.8	<0.001	-6312.5	-4359.1	-4902.6	<0.001	-5994.2	-3810.9	-4421.6	<0.001	-5396.5	-3446.7
**Remote**	4773.4	<0.001	4501.0	5045.8	5190.0	<0.001	4890.3	5489.7	4914.0	<0.001	4676.1	5151.8
**Did not attend (DNA)**	-359.9	<0.001	-533.0	-186.8	-222.4	<0.001	-417.1	-27.6	-139.1	0.118	-310.9	32.8
Number of antipsychotic mentions	**β coefficient: number of weekly antipsychotics**	**p**	**Lower limit**	**Upper limit**	**β coefficient: number of weekly antipsychotics**	**p**	**Lower limit**	**Upper limit**	**β coefficient: number of weekly antipsychotics**	**p**	**Lower limit**	**Upper limit**
**Any antipsychotic**	42.2	0.559	-98.5	182.9	104.8	0.189	-49.5	259.0	122.4	0.087	-15.1	259.9
**Any depot**	58.1	0.015	12.5	103.7	21.9	0.412	-30.0	73.7	32.5	0.177	-14.0	79.1
**Any oral**	-15.4	0.797	-131.8	101.1	81.8	0.208	-43.9	207.6	92.4	0.112	-19.8	204.7
Number of individual antipsychotic mentions	**β coefficient: number of weekly antipsychotics**	**p**	**Lower limit**	**Upper limit**	**β coefficient: number of weekly antipsychotics**	**p**	**Lower limit**	**Upper limit**	**β coefficient: number of weekly antipsychotics**	**p**	**Lower limit**	**Upper limit**
**Amisulpiride**	-7.9	0.179	-19.2	3.5	0.7	0.912	-11.9	13.3	-4.3	0.455	-15.5	6.9
**Aripiprazole**	-3.4	0.854	-39.6	32.7	31.4	0.130	-8.7	71.4	38.2	0.041	2.4	74.0
**Clozapine**	1.5	0.931	-31.8	34.7	18.2	0.342	-19.0	55.4	10.9	0.507	-21.0	42.8
**Haloperidol**	5.6	0.118	-1.3	12.6	2.0	0.596	-5.4	9.5	-3.2	0.352	-9.8	3.4
**Lurasidone**	0.6	0.868	-6.0	7.1	1.2	0.745	-6.0	8.5	4.0	0.238	-2.6	10.5
**Olanzapine**	-2.3	0.903	-39.2	34.6	13.5	0.513	-26.7	53.7	13.7	0.457	-22.1	49.5
**Quetiapine**	-8.8	0.385	-28.5	10.9	25.5	0.025	3.8	47.2	17.0	0.103	-3.1	37.1
**Risperidone**	-5.4	0.625	-27.0	16.2	4.8	0.688	-18.4	28.0	3.8	0.728	-17.4	25.0
**Sulpiride**	-0.3	0.778	-2.7	2.0	0.9	0.511	-1.8	3.6	-1.1	0.360	-3.6	1.3
**Zuclopenthixol**	0.0	0.985	-2.8	2.8	1.2	0.467	-2.0	4.3	2.6	0.080	-0.3	5.4
Number of antipsychotic depot mentions	**β coefficient: number of weekly depots**	**p**	**Lower limit**	**Upper limit**	**β coefficient: number of weekly depots**	**p**	**Lower limit**	**Upper limit**	**β coefficient: number of weekly depots**	**p**	**Lower limit**	**Upper limit**
**Aripiprazole depot**	32.4	<0.001	17.9	46.8	26.4	0.003	9.6	43.1	32.8	<0.001	17.6	47.9
**Paliperidone depot**	24.6	0.059	-0.4	49.6	7.3	0.618	-21.3	35.9	12.8	0.326	-12.5	38.1
**Haloperidol depot**	-4.6	0.281	-12.9	3.7	-5.1	0.291	-14.5	4.3	-3.6	0.405	-12.0	4.8
**Risperidone depot**	-4.3	0.176	-10.6	1.9	-13.6	<0.001	-20.1	-7.1	3.8	0.728	-17.4	25.0
**Zuclopenthixol depot**	4.2	0.342	-4.4	12.7	0.3	0.957	-9.4	10.0	-2.6	0.565	-11.5	6.3
Number of mood stabiliser mentions	**β coefficient: number of weekly mood stabilisers**	**p**	**Lower limit**	**Upper limit**	**β coefficient: number of weekly mood stabilisers**	**p**	**Lower limit**	**Upper limit**	**β coefficient: number of weekly mood stabilisers**	**p**	**Lower limit**	**Upper limit**
**Any mood stabiliser**	-36.5	0.053	-72.7	-0.3	-8.3	0.686	-48.4	31.8	-3.9	0.833	-39.7	32.0
Number of individual mood stabiliser mentions	**β coefficient: number of weekly mood stabilisers**	**p**	**Lower limit**	**Upper limit**	**β coefficient: number of weekly mood stabilisers**	**p**	**Lower limit**	**Upper limit**	**β coefficient: number of weekly mood stabilisers**	**p**	**Lower limit**	**Upper limit**
**Carbamazepine**	-6.2	0.025	-11.4	-0.9	-8.3	0.007	-14.1	-2.5	-3.4	0.211	-8.6	1.9
**Lamotrigine**	-14.5	0.046	-28.5	-0.6	-7.0	0.385	-22.6	8.6	-5.1	0.476	-19.1	8.9
**Lithium**	-0.1	0.981	-11.9	11.6	5.4	0.426	-7.8	18.6	9.5	0.122	-2.3	21.2
**Valproate**	-17.5	0.058	-35.2	0.2	-6.4	0.525	-25.9	13.1	-8.1	0.368	-25.5	9.4

Index period vs August and September 2020

Variable	August 2020: β coefficient	p	95% CI Lower limit	95% CI Upper limit	September 2020: β coefficient	p	95% CI Lower limit	95% CI Upper limit
Number of patients enrolled in mental health services	**β coefficient: number of patients**	**p**	**Lower limit**	**Upper limit**	**β coefficient: number of patients**	**p**	**Lower limit**	**Upper limit**
**Total**	-778.7	0.004	-1288.3	-269.1	-901.3	0.004	-1489.5	-313.0
**Female**	-398.2	0.013	-700.9	-95.5	-457.4	0.013	-806.8	-108.1
**Male**	-401.3	<0.001	-612.0	-190.7	-464.1	<0.001	-707.2	-220.9
**Under 18 years**	572.1	0.003	211.6	932.6	480.8	0.028	64.5	897.2
**18 to 64 years**	-951.2	<0.001	-1149.5	-752.9	-1020.1	<0.001	-1248.2	-792.1
**65 years and older**	-403.5	<0.001	-444.6	-362.4	-366.3	<0.001	-413.7	-318.9
Number of contacts with mental health professionals	**β coefficient: number of weekly contacts**	**p**	**Lower limit**	**Upper limit**	**β coefficient: number of weekly contacts**	**p**	**Lower limit**	**Upper limit**
**In person**	-4388.9	<0.001	-5478.9	-3299.0	-4401.4	<0.001	-5661.8	-3141.1
**Remote**	5571.0	<0.001	5308.0	5834.1	5008.2	<0.001	4672.4	5344.0
**Did not attend (DNA)**	-173.5	0.082	-365.0	18.1	-278.9	0.018	-502.5	-55.3
Number of antipsychotic mentions	**β coefficient: number of weekly antipsychotics**	**p**	**Lower limit**	**Upper limit**	**β coefficient: number of weekly antipsychotics**	**p**	**Lower limit**	**Upper limit**
**Any antipsychotic**	102.7	0.325	-99.8	305.2	3.0	0.980	-231.0	237.0
**Any depot**	59.3	0.110	12.3	130.9	17.2	0.683	-65.2	99.6
**Any oral**	51.9	0.522	-106.1	210.0	-17.3	0.854	-200.8	166.2
Number of individual antipsychotic mentions	**β coefficient: number of weekly antipsychotics**	**p**	**Lower limit**	**Upper limit**	**β coefficient: number of weekly antipsychotics**	**p**	**Lower limit**	**Upper limit**
**Amisulpiride**	-3.5	0.603	-16.8	9.7	-15.9	0.047	-31.2	-0.6
**Aripiprazole**	18.1	0.495	-33.6	69.8	-5.5	0.857	-65.3	54.2
**Clozapine**	31.4	0.100	-5.3	68.1	5.1	0.813	-37.2	47.5
**Haloperidol**	-7.6	0.087	-16.2	1.0	-1.4	0.785	-11.8	8.9
**Lurasidone**	2.9	0.472	-5.0	10.8	-2.6	0.581	-11.8	6.6
**Olanzapine**	-0.6	0.983	-53.0	51.9	-10.3	0.742	-71.6	50.9
**Quetiapine**	8.3	0.551	-18.7	35.2	4.9	0.758	-26.2	36.1
**Risperidone**	-20.5	0.183	-50.3	9.3	-23.5	0.190	-58.2	11.2
**Sulpiride**	-1.7	0.230	-4.4	1.0	-1.7	0.291	-4.8	1.4
**Zuclopenthixol**	3.3	0.052	0.0	6.6	-0.4	0.824	-4.1	3.3
Number of antipsychotic depot mentions	**β coefficient: number of weekly depots**	**p**	**Lower limit**	**Upper limit**	**β coefficient: number of weekly depots**	**p**	**Lower limit**	**Upper limit**
**Aripiprazole depot**	41.5	<0.001	23.6	59.5	37.6	0.001	16.7	58.6
**Paliperidone depot**	18.4	0.242	-12.1	49.0	-8.0	0.655	-42.9	26.9
**Haloperidol depot**	-5.3	0.393	-17.3	6.7	-3.0	0.671	-16.9	10.8
**Risperidone depot**	-9.7	0.008	-16.6	-2.8	-9.8	0.018	-17.7	-1.9
**Zuclopenthixol depot**	12.4	0.122	-3.1	27.8	4.9	0.596	-13.1	22.8
Number of mood stabiliser mentions	**β coefficient: number of weekly mood stabilisers**	**p**	**Lower limit**	**Upper limit**	**β coefficient: number of weekly mood stabilisers**	**p**	**Lower limit**	**Upper limit**
**Any mood stabiliser**	-0.3	0.989	-48.8	48.1	-26.6	0.361	-83.2	30.0
Number of individual mood stabiliser mentions	**β coefficient: number of weekly mood stabilisers**	**p**	**Lower limit**	**Upper limit**	**β coefficient: number of weekly mood stabilisers**	**p**	**Lower limit**	**Upper limit**
**Carbamazepine**	-8.3	0.009	-14.3	-2.3	-9.3	0.012	-16.3	-2.3
**Lamotrigine**	-9.2	0.308	-26.7	8.3	-20.8	0.049	-41.0	-0.5
**Lithium**	6.6	0.409	-9.0	22.2	8.6	0.363	-9.8	27.0
**Valproate**	12.2	0.293	-10.3	34.8	-5.7	0.673	-32.1	20.7

< 0.001	<0.01	<0.05

For peer review only - http://bmjopen.bmj.com/site/about/guidelines.xhtml

The RECORD statement – checklist of items, extended from the STROBE statement, that should be reported in observational studies using routinely collected health data.

	Item No.	STROBE items	Location in manuscript where items are reported	RECORD items	Location in manuscript where items are reported
**Title and abstract**					
	1	(a) Indicate the study's design with a commonly used term in the title or the abstract (b) Provide in the abstract an informative and balanced summary of what was done and what was found	Pages 1 and 3	RECORD 1.1: The type of data used should be specified in the title or abstract. When possible, the name of the databases used should be included.<br><br>RECORD 1.2: If applicable, the geographic region and timeframe within which the study took place should be reported in the title or abstract.<br><br>RECORD 1.3: If linkage between databases was conducted for the study, this should be clearly stated in the title or abstract.	Page 3
**Introduction**					
Background rationale	2	Explain the scientific background and rationale for the investigation being reported	Page 5		
Objectives	3	State specific objectives, including any prespecified hypotheses	Page 5		
**Methods**					
Study Design	4	Present key elements of study design early in the paper	Page 6		
Setting	5	Describe the setting, locations, and relevant dates, including periods of recruitment, exposure, follow-up, and data collection	Page 6		

For peer review only

Participants	6	*(a) Cohort study* - Give the eligibility criteria, and the sources and methods of selection of participants. Describe methods of follow-up<br>*Case-control study* - Give the eligibility criteria, and the sources and methods of case ascertainment and control selection. Give the rationale for the choice of cases and controls<br>*Cross-sectional study* - Give the eligibility criteria, and the sources and methods of selection of participants<br><br>*(b) Cohort study* - For matched studies, give matching criteria and number of exposed and unexposed<br>*Case-control study* - For matched studies, give matching criteria and the number of controls per case	Page 6	RECORD 6.1: The methods of study population selection (such as codes or algorithms used to identify subjects) should be listed in detail. If this is not possible, an explanation should be provided.<br><br>RECORD 6.2: Any validation studies of the codes or algorithms used to select the population should be referenced. If validation was conducted for this study and not published elsewhere, detailed methods and results should be provided.<br><br>RECORD 6.3: If the study involved linkage of databases, consider use of a flow diagram or other graphical display to demonstrate the data linkage process, including the number of individuals with linked data at each stage.	Page 6
Variables	7	Clearly define all outcomes, exposures, predictors, potential confounders, and effect modifiers. Give diagnostic criteria, if applicable.	Pages 6 to 7	RECORD 7.1: A complete list of codes and algorithms used to classify exposures, outcomes, confounders, and effect modifiers should be provided. If these cannot be reported, an explanation should be provided.	Pages 6 to 7
Data sources/ measurement	8	For each variable of interest, give sources of data and details of methods of assessment (measurement).<br>Describe comparability of assessment methods if there is more than one group	Pages 6 to 7		

For peer review only

Bias	9	Describe any efforts to address potential sources of bias	Pages 6 to 7		
Study size	10	Explain how the study size was arrived at	Page 6		
Quantitative variables	11	Explain how quantitative variables were handled in the analyses. If applicable, describe which groupings were chosen, and why	Page 7		
Statistical methods	12	(a) Describe all statistical methods, including those used to control for confounding<br>(b) Describe any methods used to examine subgroups and interactions<br>(c) Explain how missing data were addressed<br>(d) *Cohort study* - If applicable, explain how loss to follow-up was addressed<br>*Case-control study* - If applicable, explain how matching of cases and controls was addressed<br>*Cross-sectional study* - If applicable, describe analytical methods taking account of sampling strategy<br>(e) Describe any sensitivity analyses	Page 7		
Data access and cleaning methods		..		RECORD 12.1: Authors should describe the extent to which the investigators had access to the database population used to create the study population.	Page 6

For peer review only

				RECORD 12.2: Authors should provide information on the data cleaning methods used in the study.	
Linkage		..		RECORD 12.3: State whether the study included person-level, institutional-level, or other data linkage across two or more databases. The methods of linkage and methods of linkage quality evaluation should be provided.	N/A
**Results**					
Participants	13	(a) Report the numbers of individuals at each stage of the study (*e.g.*, numbers potentially eligible, examined for eligibility, confirmed eligible, included in the study, completing follow-up, and analysed)<br>(b) Give reasons for non-participation at each stage.<br>(c) Consider use of a flow diagram	Page 8/Supplementary Table 1	RECORD 13.1: Describe in detail the selection of the persons included in the study (*i.e.,* study population selection) including filtering based on data quality, data availability and linkage. The selection of included persons can be described in the text and/or by means of the study flow diagram.	N/A
Descriptive data	14	(a) Give characteristics of study participants (*e.g.*, demographic, clinical, social) and information on exposures and potential confounders<br>(b) Indicate the number of participants with missing data for each variable of interest<br>(c) *Cohort study* - summarise follow-up time (*e.g.*, average and total amount)	Page 8/Supplementary Table 1		
Outcome data	15	*Cohort study* - Report numbers of outcome events or summary measures over time<br>*Case-control study* - Report numbers in each exposure	Pages 8 to 9/Supplementary Table 1		

For peer review only

		category, or summary measures of exposure<br>*Cross-sectional study* - Report numbers of outcome events or summary measures			
Main results	16	(a) Give unadjusted estimates and, if applicable, confounder-adjusted estimates and their precision (e.g., 95% confidence interval). Make clear which confounders were adjusted for and why they were included<br>(b) Report category boundaries when continuous variables were categorized<br>(c) If relevant, consider translating estimates of relative risk into absolute risk for a meaningful time period	Supplementary Table 1		
Other analyses	17	Report other analyses done—e.g., analyses of subgroups and interactions, and sensitivity analyses	N/A		
**Discussion**					
Key results	18	Summarise key results with reference to study objectives	Page 10		
Limitations	19	Discuss limitations of the study, taking into account sources of potential bias or imprecision. Discuss both direction and magnitude of any potential bias	Pages 11 to 12	RECORD 19.1: Discuss the implications of using data that were not created or collected to answer the specific research question(s). Include discussion of misclassification bias, unmeasured confounding, missing data, and changing eligibility over time, as they pertain to the study being reported.	Pages 11 to 12
Interpretation	20	Give a cautious overall interpretation of results considering objectives,	Pages 11 to 12		

For peer review only

		limitations, multiplicity of analyses, results from similar studies, and other relevant evidence			
Generalisability	21	Discuss the generalisability (external validity) of the study results	Pages 11 to 12		
**Other Information**					
Funding	22	Give the source of funding and the role of the funders for the present study and, if applicable, for the original study on which the present article is based	Page 2		
Accessibility of protocol, raw data, and programming code		..		RECORD 22.1: Authors should provide information on how to access any supplemental information such as the study protocol, raw data, or programming code.	Page 2

*Reference: Benchimol EI, Smeeth L, Guttmann A, Harron K, Moher D, Petersen I, Sørensen HT, von Elm E, Langan SM, the RECORD Working Committee. The REporting of studies Conducted using Observational Routinely-collected health Data (RECORD) Statement. *PLoS Medicine* 2015; in press.

*Checklist is protected under Creative Commons Attribution (CC BY) license.

For peer review only

BMJ Open

Impact of the COVID-19 pandemic on remote mental healthcare and prescribing in psychiatry: an electronic health record study

Journal:	*BMJ Open*
Manuscript ID	bmjopen-2020-046365.R1
Article Type:	Original research
Date Submitted by the Author:	17-Feb-2021
Complete List of Authors:	Patel, Rashmi; King's College London Institute of Psychiatry Psychology and Neuroscience, Department of Psychosis Studies<br>Irving, Jessica; King's College London Institute of Psychiatry Psychology and Neuroscience, Department of Psychosis Studies<br>Brinn, Aimee; King's College London Institute of Psychiatry Psychology and Neuroscience, Department of Psychosis Studies<br>Broadbent, Matthew; South London and Maudsley Mental Health NHS Trust<br>Shetty, Hitesh; South London and Maudsley Mental Health NHS Trust, Biomedical Research Centre Nucleus<br>Pritchard, Megan; South London and Maudsley Mental Health NHS Trust, Institute of Psychiatry, Psychology and Neuroscience<br>Downs, Johnny; King's College London Institute of Psychiatry Psychology and Neuroscience<br>Stewart, Robert; King's College London Institute of Psychiatry Psychology and Neuroscience<br>Harland, Robert; South London and Maudsley Mental Health NHS Trust<br>McGuire, Philip; King's College London Institute of Psychiatry Psychology and Neuroscience, Department of Psychosis Studies
**Primary Subject Heading**:	Mental health
Secondary Subject Heading:	Epidemiology, Health informatics, Health services research, Mental health
Keywords:	EPIDEMIOLOGY, Telemedicine < BIOTECHNOLOGY & BIOINFORMATICS, Health informatics < BIOTECHNOLOGY & BIOINFORMATICS, MENTAL HEALTH, PSYCHIATRY

SCHOLARONE™
Manuscripts

I, the Submitting Author has the right to grant and does grant on behalf of all authors of the Work (as defined in the below author licence), an exclusive licence and/or a non-exclusive licence for contributions from authors who are: i) UK Crown employees; ii) where BMJ has agreed a CC-BY licence shall apply, and/or iii) in accordance with the terms applicable for US Federal Government officers or employees acting as part of their official duties; on a worldwide, perpetual, irrevocable, royalty-free basis to BMJ Publishing Group Ltd ("BMJ") its licensees and where the relevant Journal is co-owned by BMJ to the co-owners of the Journal, to publish the Work in this journal and any other BMJ products and to exploit all rights, as set out in our licence.

The Submitting Author accepts and understands that any supply made under these terms is made by BMJ to the Submitting Author unless you are acting as an employee on behalf of your employer or a postgraduate student of an affiliated institution which is paying any applicable article publishing charge ("APC") for Open Access articles. Where the Submitting Author wishes to make the Work available on an Open Access basis (and intends to pay the relevant APC), the terms of reuse of such Open Access shall be governed by a Creative Commons licence – details of these licences and which Creative Commons licence will apply to this Work are set out in our licence referred to above.

Other than as permitted in any relevant BMJ Author's Self Archiving Policies, I confirm this Work has not been accepted for publication elsewhere, is not being considered for publication elsewhere and does not duplicate material already published. I confirm all authors consent to publication of this Work and authorise the granting of this licence.

For peer review only

Impact of the COVID-19 pandemic on remote mental healthcare and prescribing in psychiatry: an electronic health record study

Rashmi Patel*[1,2], Jessica Irving*[1], Aimee Brinn[1], Matthew Broadbent[2], Hitesh Shetty[2], Megan Pritchard[1,2], Johnny Downs[1,2], Robert Stewart[1,2], Robert Harland[2], Philip McGuire[1,2]

1. King's College London (Institute of Psychiatry, Psychology and Neuroscience), London, UK
2. South London and Maudsley NHS Foundation Trust, London, UK

*Equal contributions to authorship

Correspondence to:

Rashmi Patel

E-mail: bmj@rpatel.co.uk

Manuscript word count (excluding references): 4,931

Abstract word count: 281

Keywords: SARS-CoV2, telepsychiatry, telemedicine, electronic health records, mental health

Licence declaration: I, the Submitting Author has the right to grant and does grant on behalf of all authors of the Work (as defined in the below author licence), an exclusive licence and/or a non-exclusive licence for contributions from authors who are: i) UK Crown employees; ii) where BMJ has agreed a CC-BY licence shall apply, and/or iii) in accordance with the terms applicable for US Federal Government officers or employees acting as part of their official duties; on a worldwide, perpetual, irrevocable, royalty-free basis to BMJ Publishing Group Ltd ("BMJ") its licensees and where the relevant Journal is co-owned by BMJ to the co-owners of the Journal, to publish the Work in BMJ Open and any other BMJ products and to exploit all rights, as set out in our licence.

The Submitting Author accepts and understands that any supply made under these terms is made by BMJ to the Submitting Author unless you are acting as an employee on behalf of your employer or a postgraduate student of an affiliated institution which is paying any applicable article publishing charge ("APC") for Open Access articles. Where the Submitting Author wishes to make the Work available on an Open Access basis (and intends to pay the relevant APC), the terms of reuse of such Open Access shall be governed by a Creative Commons licence – details of these licences and which Creative Commons licence will apply to this Work are set out in our licence referred to above.

For peer review only

Impact of the COVID-19 pandemic on remote mental healthcare and prescribing in psychiatry: an electronic health record study

For peer review only

For peer review only - http://bmjopen.bmj.com/site/about/guidelines.xhtml

Competing interests: All authors have completed the ICMJE uniform disclosure form at www.icmje.org/coi_disclosure.pdf and declare: RS has received funding from Janssen, GSK and Takeda outside the submitted work. RP has received funding from Janssen, Induction Healthcare and Holmusk outside the submitted work. The other authors declare no competing interests.

Author contributions: The study was conceived by RP. Data extraction and statistical analysis were performed by JI supervised by RP and supported by MB and HS. Reporting of findings were carried out by RP, JI and AB. All authors (RP, JI, AB, MB, HS, MP, JD, RS, RH, PM) contributed to study design, manuscript preparation and approved the final version.

Ethics approval: The CRIS data resource received ethical approval as a de-identified dataset for secondary mental health research analyses from Oxfordshire REC C (Ref: 18/SC/0372).

Source of funding: MB, HS, MP, RS and PM have received funding from the National Institute for Health Research (NIHR) Biomedical Research Centre at South London and Maudsley NHS Foundation Trust and King's College London, which also supports the development and maintenance of the BRC Case Register. RP has received funding from a Medical Research Council (MRC) Health Data Research UK Fellowship (MR/S003118/1) and a Starter Grant for Clinical Lecturers (SGL015/1020) supported by the Academy of Medical Sciences, The Wellcome Trust, MRC, British Heart Foundation, Arthritis Research UK, the Royal College of Physicians and Diabetes UK. RS has received funding from a Medical Research Council (MRC) Mental Health Data Pathfinder Award to King's College London, an NIHR Senior Investigator Award and the National Institute for Health Research (NIHR) Applied Research Collaboration South London (NIHR ARC South London) at King's College Hospital NHS Foundation Trust.

Role of funder: The views expressed are those of the authors and not necessarily those of the funders. The funders had no role in the design and conduct of the study; collection, management, analysis, and interpretation of the data; preparation, review, or approval of the manuscript; and decision to submit the manuscript for publication.

Data sharing: The data accessed by CRIS remain within an NHS firewall and governance is provided by a patient-led oversight committee. Access to data is restricted to honorary or substantive employees of the South London and Maudsley NHS Foundation Trust and governed by a local oversight committee who review and approve applications to extract and analyse data for research. Subject to these conditions, data access is encouraged and those interested should contact RS (robert.stewart@kcl.ac.uk), CRIS academic lead.

For peer review only

Abstract

Objectives:

The recent COVID-19 pandemic has disrupted mental healthcare delivery, with many services shifting from in-person to remote patient contact. We investigated the impact of the pandemic on the use of remote consultation and on the prescribing of psychiatric medications.

Design and setting:

The Clinical Record Interactive Search tool (CRIS) was used to examine de-identified electronic health records (EHRs) of people receiving mental healthcare from the South London and Maudsley (SLaM) NHS Foundation Trust. Data from the period before and after the onset of the pandemic were analysed using linear regression, and visualised using locally estimated scatterplot smoothing (LOESS).

Participants:

All patients receiving care from SLaM between 7th January 2019 and 20th September 2020 (around 37,500 patients per week).

Outcome measures:

i) The number of clinical contacts (in-person, remote or non-attended) with mental healthcare professionals per week
ii) Prescribing of antipsychotic and mood stabiliser medications per week

Results:

Following the onset of the pandemic, the frequency of in-person contacts was significantly reduced compared to that in the previous year (β coefficient: -5829.6 contacts, 95% CI -6919.5 to -4739.6, p<0.001), while the frequency of remote contacts significantly increased (β coefficient: 3338.5 contacts, 95% CI 3074.4 to 3602.7, p<0.001). Rates of remote consultation were lower in older adults than in working age adults, children and adolescents. Despite this change in the type of patient contact, antipsychotic and mood stabiliser prescribing remained at similar levels.

Conclusions:

The COVID-19 pandemic has been associated with a marked increase in remote consultation, particularly among younger patients. However, there was no evidence that this has led to changes in psychiatric prescribing. Nevertheless, further work is needed to ensure that older patients are able to access mental healthcare remotely.

For peer review only - http://bmjopen.bmj.com/site/about/guidelines.xhtml

Article Summary

Strengths and limitations

- The use of electronic health record data allowed rapid analysis of the associations of the COVID-19 pandemic with remote mental healthcare in a large sample of patients.
- Natural language processing (NLP) tools enabled access to free text data on medication prescribing in electronic health records.
- The large sample size increases the robustness of our findings and generalisability to real-world clinical practice.
- A short follow-up period precluded the investigation of associations between remote consultation, diagnoses and clinical outcomes.
- The study analysed secondary mental healthcare data which may not have included information on psychiatric prescribing in primary care.

For peer review only

Introduction

The COVID-19 pandemic caused by the SARS-CoV-2 coronavirus, and accompanying physical distancing and travel restrictions, has had a tremendous impact on the delivery of healthcare worldwide. It has particularly affected mental healthcare, making it more difficult for people to be seen in-person and harder to deliver treatments.[1–3] The pandemic has also increased the exposure of patients to factors known to exacerbate mental health disorders, including isolation, unemployment, inactivity and decreased social support.[4] The extent to which these changes are impacting on mental health is unknown,[5] but is critical to the ongoing reconfiguration of services in response to the current pandemic.[4]

The recent advent of technology to support remote communication in healthcare (telemedicine)[6] has enabled a shift from in-person to remote clinical contact in response to the COVID-19 pandemic. This has facilitated healthcare following the adoption of travel and physical distancing restrictions originally developed by the UK government in response to the spread of SARS-CoV-2.[7]

Prior to the COVID-19 pandemic, the application of telemedicine in mental health has been limited, and little is known about the clinical and sociodemographic factors that may influence the effectiveness of remote consultation in this context.[8] As the current pandemic evolves, while travel and physical distancing restrictions are expected to continue,[9–11] remote consultation is likely to constitute a major component of mental healthcare until the pandemic is over. Furthermore, it is possible that in the absence of a pandemic, remote consultation could help to improve access to and reduce costs of delivering community mental healthcare.[7] Thus, it is critical to determine the impact, potential benefits and disadvantages of remote consultation in mental healthcare.

Electronic health record (EHR) studies provide an opportunity to look at patterns of health care service delivery across populations.[12] Natural language processing (NLP) techniques can be used to enrich EHR datasets by extracting clinical relevant data from assessments, reviews and correspondence documented as unstructured free text.[13–16] Web-based data visualisation platforms can then be used to present population data derived from EHRs to support healthcare policy making.[17,18]

In the present study, we analysed EHR data from a large provider of mental healthcare in South London (UK) to assess the impact of the current COVID-19 pandemic on rates of remote consultation and prescribing of psychiatric medications.

For peer review only

For peer review only - http://bmjopen.bmj.com/site/about/guidelines.xhtml

Methods

Participants and setting

We extracted data from de-identified EHRs for individuals accessing secondary mental health care within the South London and Maudsley (SLaM) NHS Foundation Trust from 7th January 2019 to 20th September 2020. SLaM holds EHRs for over 450,000 people who have received mental healthcare since 2007.[12] The Trust provides community and inpatient mental healthcare within its catchment area which includes the London boroughs of Lambeth, Southwark, Croydon and Lewisham. Its service provision covers the following specialty groupings: Addictions; Behavioural and Developmental Psychiatry; Child and Adolescent Mental Health Services; Mental Health of Older Adults and Dementia; Mood, Anxiety and Personality; Psychological Medicine, and Psychosis. Its services are structured into three age groups: children and adolescents (under 18 years), working age adults (18 to 64 years) and older adults (65 years plus).[12] SLaM's catchment area varies considerably in terms of ethnic composition, education, urbanicity and area-level deprivation.[12,19] Overall the SLaM catchment boroughs are representative of London as a whole in terms of age, gender, education and socioeconomic status.[19]

Data extraction

We extracted separate datasets for each week from January 2019 to September 2020. We chose this time period to provide a whole calendar year of data representing typical clinical activity prior to the onset of the COVID-19 pandemic, which was declared by the World Health Organisation in March 2020.[20] The cohort for each week included all patients with an active referral to SLaM, defined as having been accepted to a SLaM community or inpatient service on, or before the Sunday of each week and having been discharged on, or after the Monday of the same week (for those patients discharged from SLaM clinical services prior to September 2020). This method was chosen to enable assessment of changes in the frequency of data in the context of the number of active patients within a given week, taking into account fluctuations in rates of acceptance to, and discharge from clinical services during the period of data analysis, and the fact that some patients may have had an active referral to SLaM for a duration of less than a week (e.g. patients who receive a single assessment but are then discharged without further follow-up).

The Clinical Record Interactive Search (CRIS) tool was used to extract data. CRIS is a bespoke platform and governance framework to support an electronic mental health case register whose infrastructure is based on Microsoft Structured Query Language (SQL) and provides an automated pipeline for EHR data de-identification, database assembly and query to facilitate analyses of EHR-derived data of patients receiving care from SLaM.[12,19] The source EHR data are generated by SLaM clinicians, including psychiatrists, psychologists, nurses and other health and social care professionals who document clinical assessments, reviews and progress in the provision of mental healthcare in the community or psychiatric hospitals. The dataset has received ethical approval from Oxfordshire REC C (Ref: 18/SC/0372) for secondary analysis of de-identified EHR data to support mental health research. Access to data is restricted to honorary or substantive employees of SLaM.

Patient and public involvement

The project was approved by the local SLaM CRIS Oversight Committee (chaired by a service user representative) who review and approve applications to extract data for research. The CRIS infrastructure was designed and is managed with ongoing service user input to ensure all research projects comply with data governance, legal and ethical guidelines. Further details are available on the SLaM CRIS website.[21]

For peer review only - http://bmjopen.bmj.com/site/about/guidelines.xhtml

Defined variables

Data were extracted on the number of active patients at any point in a given week (referred to as "patients registered with SLaM services"), age, gender, the number of contacts with mental healthcare professionals that were attended in-person, remotely or where a patient did not attend (DNA), and psychotropic medications (antipsychotic drugs and mood stabilisers).

Data on contacts with mental healthcare professionals were obtained from the *Events* input field in the EHR. Clinicians use the *Events* field to record the content and outcome of clinical appointments with patients. When an appointment for the patient to be assessed or reviewed by a clinician is scheduled, the clinician (or a healthcare service administrator) will create an *Event* and record the appointment date and time, whether the appointment was attended by the patient, and the *Event Type* (i.e. in-person or a remote contact). Data on contacts with mental healthcare professionals were defined as follows:

(i) In-person contacts: appointments attended by a patient and clinician recorded as a "Face To Face" or "Group Contact" *Event Type*. The "Face To Face" *Event Type* refers to an appointment which is conducted with both the patient and clinician present in the same physical space which could be at a mental health service clinic/outpatient department or an alternative location such as the patient's own home. The "Group Contact" *Event Type* refers to an appointment conducted in a group setting within the same physical space (i.e. multiple patients and one or more clinicians, e.g. as part of group psychological therapy).

(ii) Remote contacts: defined as appointments attended by a patient and clinician recorded as a "Phone" or "Video (virtual) appointment" *Event Type*. We did not analyse data on written forms of remote contact between patients and clinicians (recorded as "Email", "Letter", "Mail" or "Short Message Text (SMS)" *Event Type*) as these methods of contact are not necessarily attended by the patient and clinician contemporaneously and the date of the recorded *Event* may not correspond with the date of the written communication being received by the patient.

(iii) Did not attend (DNA) contacts: defined as any unplanned appointment cancellation (in-person or remote).

Age was determined according to the age of each active patient on the Sunday of each week and was categorised in groups defined as under 18 years, 18 to 64 years and 65 years and over. These groups were chosen to reflect the age group structure of SLaM clinical services which provide care for children and adolescents, working age adults and older adults respectively.[12]

We extracted recorded mentions of antipsychotics and mood stabilisers using two sources: structured input fields within the EHR to record prescribed psychotropic medications and natural language processing (NLP) tools applied to free text EHR data including documentation of clinical assessments, and progress notes, clinical reports and correspondence between clinicians. The development of these NLP tools and example use cases have been previously described;[12,16,22,23] details on manual validation can be found on the CRIS website.[24]

Antipsychotics were defined according to the Maudsley Guidelines for Advanced Prescribing in Psychosis[25] and grouped as oral antipsychotics and long-acting injectable (LAI) depot antipsychotics. A further analysis of the most frequently prescribed antipsychotics was conducted including amisulpride, aripiprazole, clozapine, haloperidol, lurasidone, quetiapine, olanzapine, risperidone, sulpiride, zuclopenthixol, aripiprazole LAI, paliperidone LAI (including both 1-monthly and 3-monthly preparations), haloperidol LAI, risperidone LAI and zuclopenthixol LAI. Non-antipsychotic mood stabilisers were defined as carbamazepine, lamotrigine, lithium and valproate. The analyses focused on antipsychotics and mood stabilisers because these are the most frequently prescribed psychotropic medications in UK secondary mental healthcare. We chose not to analyse data on antidepressants, as these are often initiated in primary care and these data are not comprehensively represented in the secondary mental healthcare dataset analysed in this study.

For peer review only - http://bmjopen.bmj.com/site/about/guidelines.xhtml

Data analysis and visualisation

Data were cleaned and visualised using Exploratory Version 6.2.2 (https://docs.exploratory.io/), a user interface frontend to R 4.0.x for data analysis and visualisation. Results were communicated via an interactive dashboard which enables users to zoom and scroll within charts to highlight specific data points or series. This platform also facilitates updates to the dataset over time beyond the date of publication of this article and enables rapid visualisation of data to inform future healthcare policy. A brief guide on how to visualise data from the present study (available at http://rpatel.co.uk/TelepsychiatryDashboard) is provided in the supplementary material.

Based on the declaration of the COVID-19 pandemic in March 2020, we chose an index period of Monday 4th March 2019 to Sunday 1st March 2020 against which to compare subsequent data. We estimated the mean and 95% confidence interval for all weeks within the index period for each analysed variable. We performed separate univariate linear regression analyses to compare the weekly counts for each variable in each of the calendar months of March, April, May, June, July, August and September 2020 against the mean weekly counts in the index period. The β coefficient of each analyses represents the estimated difference from the index period mean for each variable within each analysed month. This method of monthly comparisons was chosen to enable an estimation of changes in frequency of each variable after the onset of the pandemic while taking into account weekly variations which would have occurred due to the non-working days of Easter in April and the UK bank holidays during May.

Data were aggregated and presented in charts using Exploratory software. Each data point in a given chart represents the count for the analysed variable on the Sunday of each week. Locally estimated scatterplot smoothing (LOESS) was used to superimpose a smoothed trendline (represented by a dotted line and shaded area for the confidence interval). LOESS was used to smooth out variation between each week and highlight data points for weeks indicating significant variation away from the lower or upper bound of the confidence interval. In order to effectively visualise data in limited screen space, the y-axes of charts were adjusted to fit the majority of the data points within the viewable area for each chart. When viewing the results, care should be taken to examine the y-axis to determine scale and intercept prior to interpreting data points and fitted LOESS curves within the charts. Patients with missing age and gender data were excluded from stratified age and gender charts respectively but were included in all non-stratified charts.

For peer review only - http://bmjopen.bmj.com/site/about/guidelines.xhtml

Results

Patients registered with mental health services

A mean number of 37,563 patients (95% CI 37,420 to 37,707) were registered with clinical services each week during the index period. Regression analyses indicated consistent decreases for each month of April 2020 to September 2020 in the number of patients registered per week relative to the index period, with the exception of registration for those under 18 years, which showed the opposite trend (Supplementary Table 1, Figure 1 and Supplementary Figure 1). However, the relative changes in the total number of patients registered with SLaM services during each week after April 2020 were modest, representing a maximum change of around 2% following the index period (Figure 1 and Supplementary Figure 1). The number of patients newly registered with SLaM services each week dropped between March and April followed by a modest recovery in May (Supplementary Figure 2).

For peer review only

Contacts with mental health professionals

The number of weekly contacts reduced considerably between December 2019 and January 2020, reflecting a slowdown in clinical activity during the Christmas and New Year period (Figure 2). From March 2020, in-person contacts reduced substantially from around 9,000 per week to 3,000 per week in early April 2020, corresponding with the implementation of travel and physical distancing restrictions. Over the same period there was a substantial increase in remote contacts from around 2,500 per week in early March 2020 to around 8,000 per week by the end of April 2020 (Figure 3), while the total number of clinical contacts per week dropped from around 12,500 in mid-March to around 10,000 in mid-April 2020 (Figure 2). Before March 2020 very few video consultations were conducted. Following March 2020, the frequency of video consultations increased to around almost 30% of all remote consultations by July 2020 (Supplementary Figures 3 and 4).

These visual trends were confirmed through linear regression analyses, which found consistent decreases in mean weekly in-person contacts each month from March to September relative to the previous year. This was matched by consistent increases in remote contacts compared with the previous year. The number of unattended appointments temporarily reduced in April, May, June and September 2020 (Supplementary Table 1).

It is notable that while there were reductions in clinical contacts during the early phase of the COVID-19 pandemic, these were smaller than those observed between December 2019 and January 2020, prior to the pandemic.

Visual inspection of Figure 4 illustrates the breakdown of clinical contacts per week by age. Children and adolescents under 18, working age adults and older adults show varying degrees of reduction in weekly contacts following the onset of the COVID-19 pandemic. The total number of clinical contacts for children and adolescents between mid-March and mid-May reduced to a lesser degree than for working age adults and older adults.

Visual inspection of Figure 5 indicates a compensatory increase in remote consultations for children and adolescents, with a less pronounced increase seen in patients of working age and in older adults. Although there was no clear difference between number of weekly clinical events by gender (Supplementary Figure 5), there was a greater increase in remote consultations following March 2020 in female than male patients (Supplementary Figure 6).

Antipsychotics

Visual inspection of antipsychotic reporting trends yielded two main findings: first, there was no evidence of a substantial change in total antipsychotic mentions in EHRs following the onset of the pandemic (for either oral

For peer review only - http://bmjopen.bmj.com/site/about/guidelines.xhtml

or LAI depot antipsychotics). Second, there was a large decrease in mentions of antipsychotics between December 2019 and January 2020 (Supplementary Figure 7). Free text mentions of antipsychotics outnumbered mentions in structured input fields by a factor of roughly three (Supplementary Figure 8). When grouped by age, antipsychotic mentions reduced during 2019, before increasing towards January 2020 in children and adolescents. Working age adults showed a reduction in antipsychotic mentions during 2019, with a nadir between December 2019 and January 2020. In contrast, antipsychotic mentions steadily increased among older adults over 65 (Supplementary Figure 9). There were no clear differences in antipsychotic mentions by gender (Supplementary Figure 10).

There was a statistically significant and consistent increase in weekly aripiprazole depot mentions in the months of March 2020 to September 2020 compared with mean rates across the index period. Risperidone depot mentions were reduced in March, June, August and September 2020 compared to the index period. A statistically significant increase in clozapine mentions was observed in April only ($\beta = 79.2$, $p < 0.001$) compared with the previous year (Supplementary Table 1).

Mood stabilisers

Total mentions of mood stabilisers slowly declined from the beginning of the observation period in January 2019 (Supplementary Figures 11, 12 and 13). In terms of individual mood stabilisers, from January 2019, lithium mentions increased and mentions of valproate and lamotrigine decreased (Supplementary Figure 11). Regression analysis found small decreases of marginal significance in carbamazepine and lamotrigine mentions between April 2020 and September 2020 relative to the pre-pandemic index period (Supplementary Table 1).

For peer review only - http://bmjopen.bmj.com/site/about/guidelines.xhtml

For peer review only

Discussion

We found that the onset of the COVID-19 pandemic was associated with a rapid and major shift in the mode of mental healthcare consultations, from in-person to remote in the early phase (between March and May 2020), followed by a gradual shift back towards in-person consultations over the following months. Reassuringly, these substantial changes do not appear to have been associated with dramatic shifts in the clinical activity of mental health services, as measured by the number of mentions of psychotropic medications in free text EHRs. A compensatory increase in remote mental health consultation in response to the COVID-19 pandemic mirrors similar developments within physical healthcare[26] and is in keeping with a previous analysis of working age adults using the same dataset.[27]

The shift from in-person to remote consultation was most pronounced for children and adolescents (and their parents and carers), less marked in working age adults, and least dramatic in older adults. Mental health services for older adults have not utilised remote methods for consultation to the same degree as services for working age adults and children, and this has been associated with a reduction in total clinical contacts during the early phase of the pandemic between March and May 2020. Importantly, some forms of remote contact rely on the availability of electronic hardware (a smartphone or computer), access to a stable electricity supply and high-speed internet connection, and the ability to use the technology. To enable successful remote consultation, these resources are required by both patients and clinicians, and both groups need to be able to trust the technology (with respect to safety, reliability and privacy) and be motivated to use it.[28]

From April 2020 the number of patients registered per week in mental health services decreased relative to the period of March 2019 to March 2020. This is in keeping with an earlier analysis of the same dataset examining the period from 1st February to 31st March 2020.[29] This reduction in patients registered following April 2020 may reflect the discharge of patients in the initial stages of the pandemic who may have previously had limited input from mental health services. This is supported by the fact that there was no demonstrable change in clinical activity measured through mentions of antipsychotic and mood stabiliser medications during the same period. Nonetheless, the relative reduction in number of active patients in a given week during the early stages of the pandemic was modest, representing around 2% of the total mean number of active patients between March 2019 and March 2020.

Throughout the pandemic period, the number of mentions of antipsychotics (whether oral or depot) remained steady. The only exception was an increase in mentions of aripiprazole depot, but this may reflect its recent addition to the NHS formulary. We found a reduction in mentions of antipsychotics for children and adolescents. This may be due to a reduction in the number of inpatient psychiatric beds for children and adolescents in SLaM due to the closure of two inpatient units during the same period which could have led to reduced documentation of antipsychotics in this patient group. We observed a steady increase in mentions of antipsychotics for older adults which predated the COVID-19 pandemic. The gradual increase in documentation may or may not reflect a change in prescribing (see *Strengths and limitations*), and could be related to service reconfigurations associated with changing frequency of clinical documentation and the increasing focus on monitoring of antipsychotic use in dementia.

Clozapine prescribing differs from that for other antipsychotics, in that it requires regular blood monitoring, making in-person contact essential. The increase in clozapine mentions observed in April 2020 relative to the previous year may be due to the introduction of emergency guidelines regarding frequency of full blood count monitoring to manage possible disruptions during the initial period of travel and physical distancing restrictions.[30] The increase in mentions of clozapine in April could reflect increased activity among clinicians creating treatment plans to navigate the challenges of managing full blood count monitoring for these patients.

Our analysis also revealed a decreasing trend in overall mood stabiliser mentions over time that was not related to the COVID-19 pandemic period. Notably, lithium mentions have increased and mentions of valproate and lamotrigine have decreased. The decreasing trend in valproate mentions may be explained by a reduction in prescriptions among female patients of child-bearing age due to increased awareness of its

For peer review only - http://bmjopen.bmj.com/site/about/guidelines.xhtml

teratogenic effects.[31,32] It may be that the decrease in valproate prescribing is associated with a compensatory increase in mentions of lithium, or this could be related to efforts to increase access to lithium for people suffering with treatment resistant depression or bipolar disorder.[33,34]

Implications

The lack of digital technology resources among certain vulnerable groups creates barriers to accessing remote mental healthcare. People with low incomes (over-represented among people with serious mental disorders) may not be able to afford the necessary hardware/data connection.[35] Serious mental illnesses are associated with major impairments in motivation which impair the ability of affected individuals to use remote consultation technology.[36] And many people may not have had the opportunity to access training to gain skills in using digital healthcare tools.[28] Furthermore, people with serious mental illnesses are overrepresented in deprived areas, which are the very populations at increased risk for uncontained COVID-19 transmission resulting in greater travel and physical distancing restrictions which would necessitate remote consultation to gain access to mental healthcare.[7,37]

Our research indicated that the incidence of missed appointments decreased as appointments changed from in-person to remote between April and June. Adherence to a treatment plan is an essential component for successful mental healthcare management; for people diagnosed with schizophrenia, failure to attend outpatient care following admission increases risk of relapse and readmission.[38] Relapse of serious mental illness poses large additional costs the NHS: an estimate of four times greater costs for patients who relapse, compared to those who do not.[39] Considering non-attendance at mental health care appointments is reported to be greater than most other healthcare settings,[40,41] remote methods could be harnessed more often to reduce the financial burden of non-attended appointments.[41]

Future work must urgently identify how to remove the barriers faced in access to and ability to use digital technology for remote consultation. A Canadian study found that patients receiving telephone appointments felt able to present the same information as in-person and were equally as satisfied as patients receiving in-person appointments. However, the telepsychiatry group reported lower levels of support and encouragement than in-person patients.[42] Furthermore, a systematic review, which analysed the acceptability of online and mobile phone app interventions for serious mental illness, indicated that whilst hypothetical acceptability of online and mobile phone interventions was low, actual acceptability was generally high.[43] It is possible that certain groups may feel less able to communicate their mental health difficulties remotely, whilst others may find the opposite. At the same time, clinicians may feel less able to adequately assess the mental state of patients through remote technology compared to in-person assessment. Further in-depth, qualitative research is required to elucidate the views and experiences of different patient and clinician groups.

Strengths and limitations

The availability of electronic health records in SLaM has enabled the rapid development of a data visualisation platform to assess key metrics of mental health service delivery during a period of tremendous challenge to service provision during the COVID-19 pandemic. Our findings will support mental healthcare strategy to reduce barriers to those who face difficulties accessing remote care. This data visualisation approach could be applied to other forms of EHR derived healthcare data on a real-time basis to enable healthcare service providers and policy makers to adapt service provision in time of crisis, and to measure the impact of changes to service provision on performance metrics such as access to remote care.

We were able to examine changes in the frequency of different types of mental health service consultation and mentions of psychotropic prescribing over time. However, it was not possible to analyse the nature of in-person and remote consultations. Remote consultations can take many forms and the ability to perform certain clinical tasks may vary depending on the modality employed. For example, e-mail and text messaging may enable communication between patients and clinicians, but opportunities for real-time clinical assessment and review are limited. In contrast, a phone call can provide some information on history and mental state, but the availability of a video stream enables a more detailed assessment of mental state and environment. Certain clinical tasks may have been better suited to different forms of remote consultation. NLP

For peer review only - http://bmjopen.bmj.com/site/about/guidelines.xhtml

For peer review only

could be used to interrogate the content of free text documentation of remote consultations and to compare these to documentation of in-person consultations to identify barriers to performing different clinical activities and how these could be overcome.

While we are able to examine associations with age and gender, it was not possible to meaningfully investigate variation in rates and nature of remote consultation based on psychiatric diagnosis owing to the short window of data available within the present study, and because diagnostic data are not comprehensively documented at every clinical encounter. A cohort study comparing people with different psychiatric diagnoses on the frequency of in-person, remote and non-attended consultations would help to determine which groups of patients and treating clinicians have more avidly taken up remote forms of consultation during the early phase of the pandemic and what can be done to ensure digital access across all clinical subgroups.

It is important to note that changes in frequency of clinical documentation of psychotropic medications are not necessarily correlated with changes in prescribing *per se*, and could reflect differences in the nature and frequency of EHR documentation in different clinical settings. Clinicians document medication prescribing in structured fields and free text as part of the intended treatment plan agreed with the patient, but this does not necessarily equal the number of prescriptions being dispensed by a pharmacy or being subsequently taken by the patient. It is likely that a proportion of prescriptions are not dispensed and a proportion of those which are dispensed are not taken, and that this may vary depending on the clinical setting or patient group receiving treatment. However, it is unlikely that psychiatric medications would be prescribed or dispensed in the absence of documentation as part of a treatment plan and so if there had been increases in the rates of medication prescribing, this would be correlated with an increase in the frequency of recording in the EHR.

SLaM is a secondary mental health care setting. Patients with chronic serious mental disorder may however be predominantly managed by their general practitioner meaning their psychotropic medication prescribing may not be represented within secondary mental health EHR data. This is a consistent shortcoming in secondary care EHR research. Better linkages between primary and secondary care electronic health record systems at the individual patient level would help to facilitate more comprehensive analysis of psychotropic prescribing in people receiving secondary mental healthcare. The approach taken in the present study to examine psychotropic prescribing trends is only possible in healthcare settings with electronic case registers that capture prescribing data. Only ~50% of upper-middle- and high-income countries (n=23) have adopted national EHR systems (2016 data, World Health Organisation).[44] Adoption rates of EHRs are much lower in the lower-middle (35%; n=10) and low-income countries (15%; n=3). Adoption rates for psychiatric case registers for secondary analysis in research studies, such as that held by SLaM, are far lower.

While the present dashboard focuses on medication it will also be important to assess impacts on psychological and occupational therapies. One question is whether the shift to remote contacts is associated with changes to the delivery of psychological therapies and if certain groups of people are benefitting, or are marginalised, from remote therapies. This approach could be used to identify hard-to-reach groups and develop interventions tailored towards these groups and challenges they may face accessing remote care.

Conclusions

In summary, our findings indicate that the COVID-19 pandemic has led to a rapid shift from in-person to remote methods of mental healthcare consultation, without a significant change in the overall level of clinical activity. In addition, we found marked differences in the application of remote consultation by patient age, with lower rates of among older adults, and the highest among children and adolescents. Given that travel and physical distancing restrictions related to the COVID-19 pandemic are likely to persist to some degree in the near future, it is important to ensure all people who receive and provide mental healthcare have access to the digital technology, training and clinical and social support required to access remote consultation, which is likely to continue to be an important modality to support mental health service delivery in the years following the pandemic.

For peer review only - http://bmjopen.bmj.com/site/about/guidelines.xhtml

Figure captions

Figure 1: Number of patients registered with SLaM services each week by age group

Figure 2: Total number of clinical events each week

Figure 3: Number of in-person, remote and did-not-attend (DNA) clinical events each week

Figure 4: Total number of clinical events each week by age group

Figure 5: Modes of clinical contact in different age groups

For peer review only

For peer review only - http://bmjopen.bmj.com/site/about/guidelines.xhtml

References

1 Chevance A, Gourion D, Hoertel N, *et al.* Ensuring mental health care during the SARS-CoV-2 epidemic in France: A narrative review. Encephale. 2020;**46**:193–201. doi:10.1016/j.encep.2020.04.005

2 Kozloff N, Mulsant BH, Stergiopoulos V, *et al.* The COVID-19 Global Pandemic: Implications for People With Schizophrenia and Related Disorders. *Schizophr Bull* 2020;**46**:752–7. doi:10.1093/schbul/sbaa051

3 Garriga M, Agasi I, Fedida E, *et al.* The role of mental health home hospitalization care during the COVID-19 pandemic. *Acta Psychiatr Scand* 2020;**141**:479–80. doi:10.1111/acps.13173

4 Moreno C, Wykes T, Galderisi S, *et al.* How mental health care should change as a consequence of the COVID-19 pandemic. The Lancet Psychiatry. 2020;**7**:813–24. doi:10.1016/S2215-0366(20)30307-2

5 Holmes EA, O'Connor RC, Perry VH, *et al.* Multidisciplinary research priorities for the COVID-19 pandemic: a call for action for mental health science. The Lancet Psychiatry. 2020;**7**:547–60. doi:10.1016/S2215-0366(20)30168-1

6 Bashshur R, Doarn CR, Frenk JM, *et al.* Telemedicine and the COVID-19 Pandemic, Lessons for the Future. *Telemed e-Health* 2020;**26**:571–3. doi:10.1089/tmj.2020.29040.rb

7 Chen S, Jones PB, Underwood BR, *et al.* The early impact of COVID-19 on mental health and community physical health services and their patients' mortality in Cambridgeshire and Peterborough, UK. *J Psychiatr Res* 2020;**131**:244–54. doi:10.1016/j.jpsychires.2020.09.020

8 Bokolo Anthony Jnr. Use of Telemedicine and Virtual Care for Remote Treatment in Response to COVID-19 Pandemic. *J Med Syst* 2020;**44**:1–9. doi:10.1007/s10916-020-01596-5

9 Iacobucci G. Covid-19: New restrictions to curb rising infection rate are set throughout UK. *BMJ* 2020;**370**:m3712. doi:10.1136/bmj.m3712

10 Xu S, Li Y. Beware of the second wave of COVID-19. Lancet. 2020;**395**:1321–2. doi:10.1016/S0140-6736(20)30845-X

11 Panovska-Griffiths J, Kerr CC, Stuart RM, *et al.* Determining the optimal strategy for reopening schools, the impact of test and trace interventions, and the risk of occurrence of a second COVID-19 epidemic wave in the UK: a modelling study. *Lancet Child Adolesc Heal* Published Online First: August 2020. doi:10.1016/S2352-4642(20)30250-9

12 Perera G, Broadbent M, Callard F, *et al.* Cohort profile of the South London and Maudsley NHS Foundation Trust Biomedical Research Centre (SLaM BRC) Case Register: current status and recent enhancement of an Electronic Mental Health Record-derived data resource. *BMJ Open* 2016;**6**. doi:10.1136/bmjopen-2015-008721

13 Jackson RG, Patel R, Jayatilleke N, *et al.* Natural language processing to extract symptoms of severe mental illness from clinical text: The Clinical Record Interactive Search Comprehensive Data Extraction (CRIS-CODE) project. *BMJ Open* 2017;**7**. doi:10.1136/bmjopen-2016-012012

14 Kadra G, Stewart R, Shetty H, *et al.* Antipsychotic polypharmacy prescribing and risk of hospital readmission. *Psychopharmacology (Berl)* 2018;**235**:281–9. doi:10.1007/s00213-017-4767-6

15 Kesserwani J, Kadra G, Downs J, *et al.* Risk of readmission in patients with schizophrenia and schizoaffective disorder newly prescribed clozapine. *J Psychopharmacol* 2019;**33**:449–58. doi:10.1177/0269881118817387

16 Soysal P, Perera G, Isik AT, *et al.* The relationship between polypharmacy and trajectories of cognitive decline in people with dementia: A large representative cohort study. *Exp Gerontol* 2019;**120**:62–7. doi:10.1016/j.exger.2019.02.019

17 Jean-Baptiste R, Toubiana L, Le Mignot L, *et al.* A Web-based GIS for health care decision-support. *AMIA Annu Symp Proc* 2005;**2005**:365–9.

18 Ghetian CB, Parrott R, Volkman JE, *et al.* Cancer registry policies in the United States and geographic information systems applications in comprehensive cancer control. *Health Policy (New York)* 2008;**87**:185–93. doi:10.1016/j.healthpol.2007.12.007

For peer review only - http://bmjopen.bmj.com/site/about/guidelines.xhtml

19 Stewart R, Soremekun M, Perera G, *et al.* The South London and Maudsley NHS Foundation Trust Biomedical Research Centre (SLAM BRC) case register: Development and descriptive data. *BMC Psychiatry* 2009;**9**:51. doi:10.1186/1471-244X-9-51

20 Ghebreyesus TA. WHO Director-General's opening remarks at the media briefing on COVID-19 - 11 March 2020. 2020.https://www.who.int/dg/speeches/detail/who-director-general-s-opening-remarks-at-the-media-briefing-on-covid-19---11-march-2020

21 South London and Maudsley NHS Foundation Trust. Clinical Record Interactive Search (CRIS). https://slam.nhs.uk/quality-and-research/clinical-record-interactive-search-cris/

22 Downs J, Dean H, Lechler S, *et al.* Negative Symptoms in Early-Onset Psychosis and Their Association With Antipsychotic Treatment Failure. *Schizophr Bull* 2019;**45**:69–79. doi:10.1093/schbul/sbx197

23 Downs J, Hotopf M, Ford T, *et al.* Clinical predictors of antipsychotic use in children and adolescents with autism spectrum disorders: a historical open cohort study using electronic health records. *Eur Child Adolesc Psychiatry* 2016;**25**:649–58. doi:10.1007/s00787-015-0780-7

24 CRIS Natural Language Processing Applications Library. https://www.maudsleybrc.nihr.ac.uk/facilities/clinical-record-interactive-search-cris/cris-natural-language-processing/

25 Morrison P, Taylor DM, McGuire P. *The Maudsley Guidelines on Advanced Prescribing in Psychosis*. Wiley 2019. https://books.google.co.uk/books?id=88C_DwAAQBAJ

26 Stokel-Walker C. Why telemedicine is here to stay. *BMJ* 2020;**371**:m3603. doi:10.1136/bmj.m3603

27 Stewart R, Martin E, Broadbent M. Mental health service activity during COVID-19 lockdown: South London and Maudsley data on working age community and home treatment team services and mortality from February to mid-May 2020. *medRxiv* 2020;:2020.06.13.20130419. doi:10.1101/2020.06.13.20130419

28 Scott Kruse C, Karem P, Shifflett K, *et al.* Evaluating barriers to adopting telemedicine worldwide: A systematic review. *J Telemed Telecare* 2016;**24**:4–12. doi:10.1177/1357633X16674087

29 Stewart R, Martin E, Bakolis I, *et al.* Comparison of mental health service activity before and shortly after UK social distancing responses to the COVID-19 pandemic: February-March 2020. *medRxiv* 2020;:2020.09.26.20202150. doi:10.1101/2020.09.26.20202150

30 Clozapine drug monitoring during COVID-19 for stable adult patients – SPS - Specialist Pharmacy Service – The first stop for professional medicines advice. https://www.sps.nhs.uk/articles/clozapine-drug-monitoring-in-primary-care-during-covid-19-for-stable-patients/

31 Alsdorf R, Wyszynski DF. Teratogenicity of sodium valproate. Expert Opin. Drug Saf. 2005;**4**:345–53. doi:10.1517/14740338.4.2.345

32 Taylor D, Barnes TRE, Young AH. *The Maudsley Prescribing Guidelines in Psychiatry (Thirteenth edition)*. 13th ed. 2018.

33 Voineskos D, Daskalakis ZJ, Blumberger DM. Management of Treatment-Resistant Depression: Challenges and Strategies. *Neuropsychiatr Dis Treat* 2020;**16**:221–34. doi:10.2147/NDT.S198774

34 Rybakowski JK. Challenging the Negative Perception of Lithium and Optimizing Its Long-Term Administration . Front. Mol. Neurosci. . 2018;**11**:349.https://www.frontiersin.org/article/10.3389/fnmol.2018.00349

35 Choi NG, DiNitto DM. The Digital Divide Among Low-Income Homebound Older Adults: Internet Use Patterns, eHealth Literacy, and Attitudes Toward Computer/Internet Use. *J Med Internet Res* 2013;**15**:e93. doi:10.2196/jmir.2645

36 Krzystanek M, Krysta K, Skałacka K. Treatment Compliance in the Long-Term Paranoid Schizophrenia Telemedicine Study. *J Technol Behav Sci* 2017;**2**:84–7. doi:10.1007/s41347-017-0016-4

37 Wang Q, Xu R, Volkow ND. Increased risk of COVID-19 infection and mortality in people with mental disorders: analysis from electronic health records in the United States. *World Psychiatry* 2020;**n/a**. doi:10.1002/wps.20806

For peer review only - http://bmjopen.bmj.com/site/about/guidelines.xhtml

38 Balikci A, Erdem M, Zincir S, *et al.* Adherence with Outpatient Appointments and Medication: A Two-Year Prospective Study of Patients with Schizophrenia. *Klin Psikofarmakol Bülteni-Bulletin Clin Psychopharmacol* 2013;**23**:57–64. doi:10.5455/bcp.20121130085931

39 Almond S, Knapp M, Francois C, *et al.* Relapse in schizophrenia: Costs, clinical outcomes and quality of life. *Br J Psychiatry* 2004;**184**:346–51. doi:10.1192/bjp.184.4.346

40 Mitchell AJ, Selmes T. Why don't patients attend their appointments? Maintaining engagement with psychiatric services. *Adv Psychiatr Treat* 2007;**13**:423–34. doi:10.1192/apt.bp.106.003202

41 Magnes RM. Out-patient appointments: A necessary evil? A literature review and survey of patient attendance records. Psychiatr. Bull. 2008;**32**:458–60. doi:10.1192/pb.bp.108.020065

42 Urness D, Wass M, Gordon A, *et al.* Client acceptability and quality of life - telepsychiatry compared to in-person consultation. *J Telemed Telecare* Published Online First: 2006. doi:10.1258/135763306777889028

43 Berry N, Lobban F, Emsley DcPR, *et al.* Acceptability of interventions delivered online and through mobile phones for people who experience severe mental health problems: A systematic review. J. Med. Internet Res. 2016. doi:10.2196/jmir.5250

44 World Health Organization. *Global diffusion of eHealth: Making universal health coverage achievable*. 2016. https://www.who.int/goe/publications/global_diffusion/en/

For peer review only - http://bmjopen.bmj.com/site/about/guidelines.xhtml

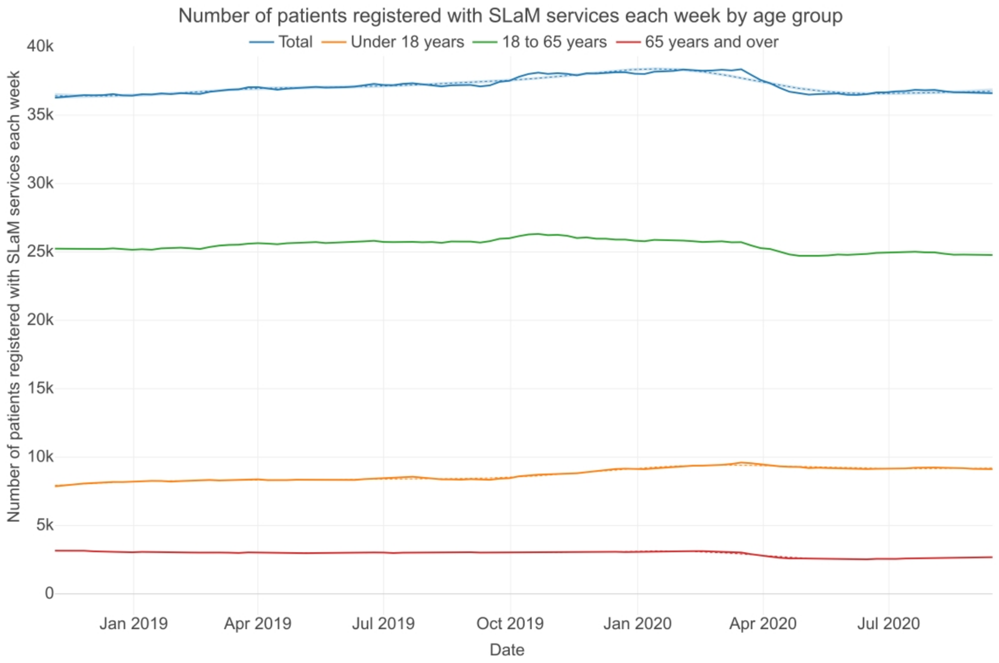

Figure 1. Number of patients registered with SLaM services each week by age group.

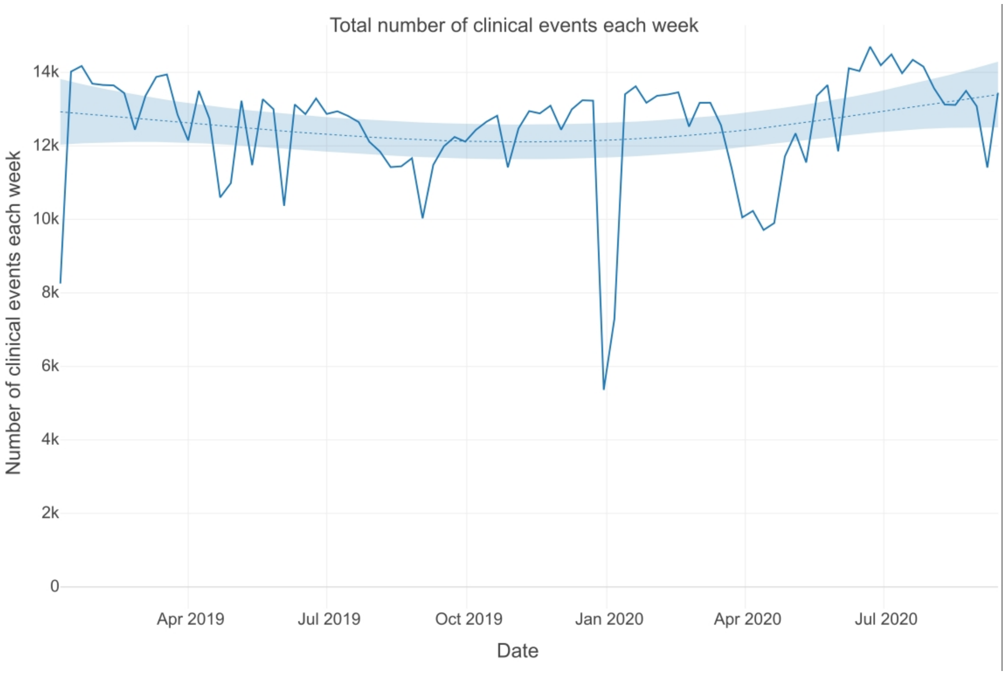

Figure 2: Total number of clinical events each week.

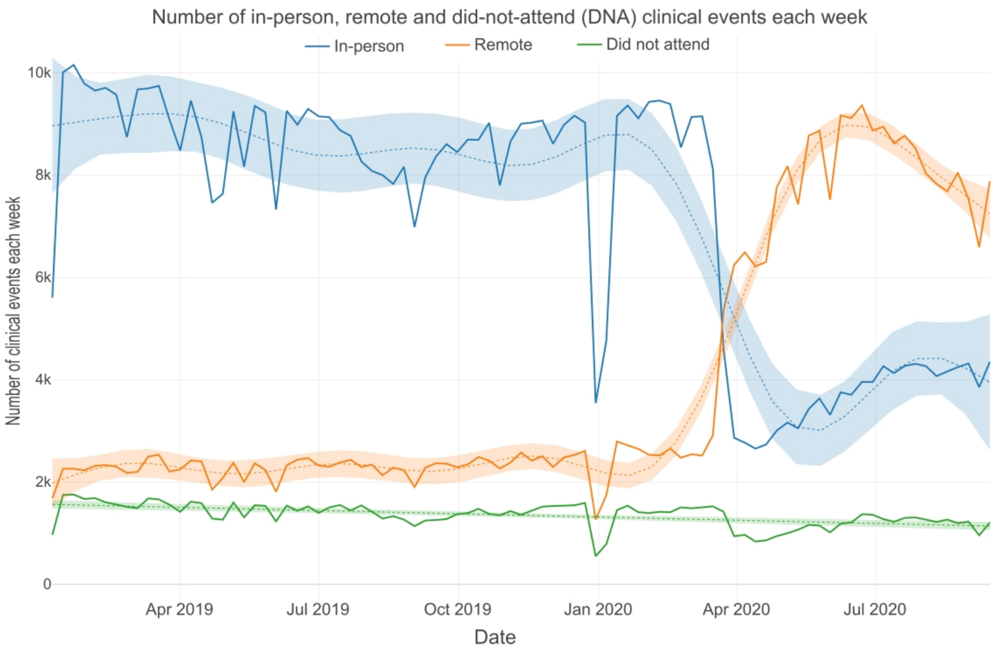

Figure 3: Number of in-person, remote and did-not-attend (DNA) clinical events each week.

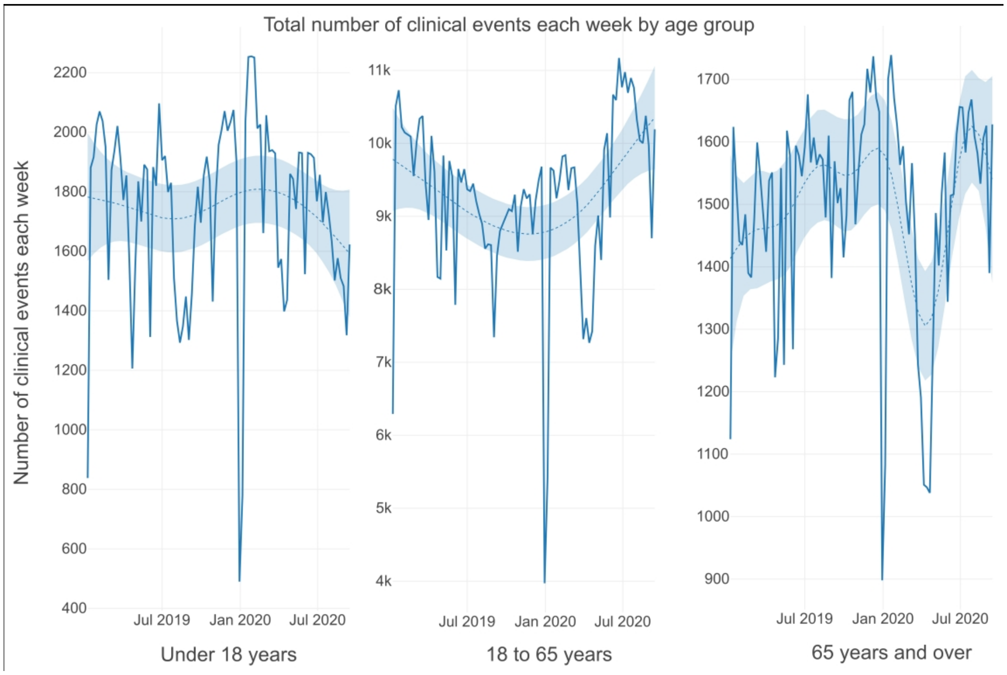

Figure 4: Total number of clinical events each week by age group. Note: The y-axes presented here do not start at zero and are not standardised between charts.

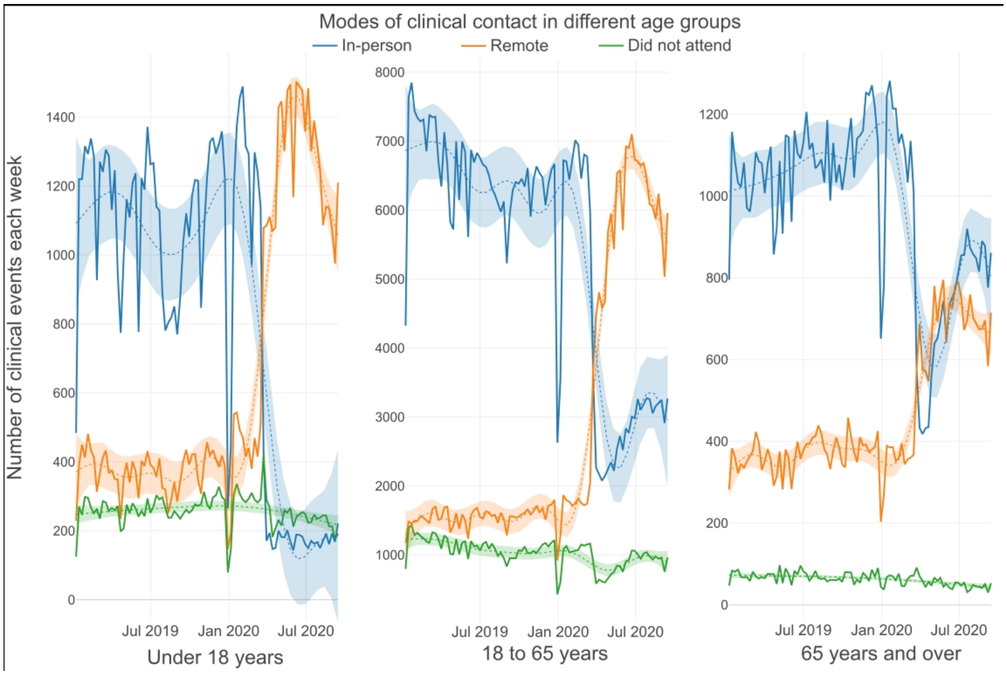

Figure 5: Modes of clinical contact in different age groups. Note: The y-axes presented here do not all start at zero and are not standardised between charts.

Supplementary Table 1

Variable	Index period: 4 March 2019 to 1 March 2020	Index period vs March 2020		95% CI	
Number of patients enrolled in mental health services	Mean (95% Confidence Interval)	β coefficient: mean number of patients	p	Lower limit	Upper limit
Total	37563.2 (37419.8 to 37706.6)	711.5	0.009	200.6	1222.5
Female	18111.4 (18026.3 to 18196.4)	470.1	0.004	167.3	773.0
Male	19355.6 (19296.5 to 19414.7)	221.9	0.044	11.0	432.8
Under 18 years	8648.3 (8546.1 to 8750.4)	877.7	<0.001	514.7	1240.7
18 to 64 years	25810.7 (24945.4 to 25010.6)	-151.5	0.140	-349.7	46.8
65 years and older	3041.4 (3029.8 to 3053.0)	-21.2	0.365	-66.6	24.2
Number of contacts with mental health professionals	Mean (95% Confidence Interval)	β coefficient: mean number of weekly contacts	p	Lower limit	Upper limit
In person	8581.8 (8325.7 to 8838.0)	-847.6	0.174	-2052.7	357.6
Remote	2328.2 (2267.3 to 2389.2)	948.3	<0.001	546.5	1350.1
Did not attend (DNA)	1411.9 (1366.8 to 1456.9)	66.4	0.501	-125.7	258.5
Number of antipsychotic mentions	Mean (95% Confidence Interval)	β coefficient: mean number of weekly antipsychotics	p	Lower limit	Upper limit
Any antipsychotic	2056.0 (2020.1 to 2091.3)	89.8	0.256	-63.4	242.9
Any depot	666.9 (655.0 to 678.7)	30.6	0.252	-21.2	82.5
Any oral	1495.2 (1465.7 to 1524.6)	55.3	0.398	-72.0	182.7
Number of individual antipsychotic mentions	Mean (95% Confidence Interval)	β coefficient: mean number of weekly antipsychotics	p	Lower limit	Upper limit
Amisulpiride	90.3 (87.4 to 93.2)	1.2	0.855	-11.8	14.2
Aripiprazole	336.6 (327.3 to 346.0)	34.1	0.102	-6.0	74.3
Clozapine	348.3 (340.0 to 356.7)	14.7	0.445	-22.7	52.0
Haloperidol	49.0 (47.2 to 50.7)	7.0	0.069	-0.4	14.5
Lurasidone	38.0 (36.3 to 39.7)	5.5	0.152	-1.9	12.8
Olanzapine	424.5 (413.4 to 435.6)	11.0	0.589	-28.6	50.6
Quetiapine	189.0 (183.9 to 194.1)	4.3	0.707	-17.8	26.3
Risperidone	238.2 (232.8 to 243.7)	-11.0	0.364	-34.4	12.5
Sulpiride	7.4 (6.7 to 8.0)	-2.3	0.092	-5.0	0.3
Zuclopenthixol	9.8 (9.1 to 10.6)	-2.1	0.209	-5.3	1.1
Number of antipsychotic depot mentions	Mean (95% Confidence Interval)	β coefficient: mean number of weekly depots	p	Lower limit	Upper limit
Aripiprazole depot	115.6 (111.8 to 119.4)	31.9	<0.001	15.6	48.1
Paliperidone depot	214.4 (207.9 to 221.0)	8.3	0.588	-21.6	38.2
Haloperidol depot	69.6 (67.5 to 71.7)	-4.8	0.303	-14.0	4.3
Risperidone depot	38.4 (36.8 to 39.9)	-7.1	0.038	-13.6	-0.5
Zuclopenthixol depot	111.2 (109.0 to 113.4)	4.5	0.373	-5.3	14.4
Number of mood stabiliser mentions	Mean (95% Confidence Interval)	β coefficient: mean number of weekly mood stabilisers	p	Lower limit	Upper limit
Any mood stabiliser	405.1 (395.7 to 414.5)	-17.8	0.391	-58.2	22.6
Number of individual mood stabiliser mentions	Mean (95% Confidence Interval)	β coefficient: mean number of weekly mood stabilisers	p	Lower limit	Upper limit
Carbamazepine	20.8 (19.4 to 22.1)	-1.0	0.729	-6.9	4.8
Lamotrigine	116.7 (113.1 to 120.4)	-14.2	0.084	-30.1	1.6
Lithium	112.4 (109.3 to 115.4)	-1.8	0.786	-15.1	11.4
Valproate	191.9 (187.3 to 196.4)	-5.4	0.588	-24.7	13.9

Variable	April 2020		95% CI		May 2020		95% CI	
Number of patients enrolled in mental health services	β coefficient: number of patients	p	Lower limit	Upper limit	β coefficient: number of patients	p	Lower limit	Upper limit
Total	-333.2	0.211	-849.6	183.2	-918.2	<0.001	-1373.5	-462.9
Female	-149.9	0.341	-455.8	156.1	-453.2	0.002	-723.1	-183.3
Male	-226.8	0.042	-440.5	-13.2	-515.6	<0.001	-703.5	-327.7
Under 18 years	723.2	<0.001	360.2	1086.3	570.7	0.001	246.4	895.1
18 to 64 years	-738.0	<0.001	-940.0	-535.9	-1076.9	<0.001	-1252.3	-901.6
65 years and older	-349.4	<0.001	-396.2	-302.6	-456.6	<0.001	-493.5	-419.7
Number of contacts with mental health professionals	β coefficient: number of weekly contacts	p	Lower limit	Upper limit	β coefficient: number of weekly contacts	p	Lower limit	Upper limit
In person	-5829.6	<0.001	-6919.5	-4739.6	-5335.8	<0.001	-6312.5	-4359.1
Remote	3338.5	<0.001	3074.4	3602.7	4773.4	<0.001	4501.0	5045.8
Did not attend (DNA)	-525.4	<0.001	-717.5	-333.2	-359.9	<0.001	-533.0	-186.8
Number of antipsychotic mentions	β coefficient: number of weekly antipsychotics	p	Lower limit	Upper limit	β coefficient: number of weekly antipsychotics	p	Lower limit	Upper limit
Any antipsychotic	10.3	0.897	-144.3	164.9	42.2	0.559	-98.5	182.9
Any depot	20.1	0.443	-30.9	71.2	58.1	0.015	12.5	103.7
Any oral	-13.9	0.830	-140.6	112.8	-15.4	0.797	-131.8	101.1
Number of individual antipsychotic mentions	β coefficient: number of weekly antipsychotics	p	Lower limit	Upper limit	β coefficient: number of weekly antipsychotics	p	Lower limit	Upper limit
Amisulpiride	-1.5	0.813	-14.2	11.2	-7.9	0.179	-19.2	3.5
Aripiprazole	-27.9	0.180	-68.1	12.4	-3.4	0.854	-39.6	32.7
Clozapine	79.2	<0.001	42.5	115.8	1.5	0.931	-31.8	34.7
Haloperidol	7.3	0.058	-0.1	14.7	5.6	0.118	-1.3	12.6
Lurasidone	-0.3	0.939	-7.6	7.0	0.6	0.868	-6.0	7.1
Olanzapine	-38.8	0.063	-78.8	1.3	-2.3	0.903	-39.2	34.6
Quetiapine	-11.3	0.325	-33.4	10.9	-8.8	0.385	-28.5	10.9
Risperidone	-32.0	0.009	-55.2	-8.7	-5.4	0.625	-27.0	16.2
Sulpiride	0.4	0.766	-2.2	3.1	-0.3	0.778	-2.7	2.0
Zuclopenthixol	0.9	0.573	-2.3	4.1	0.0	0.985	-2.8	2.8
Number of antipsychotic depot mentions	β coefficient: number of weekly depots	p	Lower limit	Upper limit	β coefficient: number of weekly depots	p	Lower limit	Upper limit
Aripiprazole depot	17.4	0.040	1.2	33.5	32.4	<0.001	17.9	46.8
Paliperidone depot	0.3	0.741	-1.3	1.9	24.6	0.059	-0.4	49.6
Haloperidol depot	7.3	0.058	-0.1	14.7	-4.6	0.281	-12.9	3.7
Risperidone depot	-6.3	0.060	-12.8	0.1	-4.3	0.176	-10.6	1.9
Zuclopenthixol depot	-6.2	0.223	-16.1	3.7	4.2	0.342	-4.4	12.7
Number of mood stabiliser mentions	β coefficient: number of weekly mood stabilisers	p	Lower limit	Upper limit	β coefficient: number of weekly mood stabilisers	p	Lower limit	Upper limit
Any mood stabiliser	-31.3	0.137	-72.1	9.4	-36.5	0.053	-72.7	-0.3
Number of individual mood stabiliser mentions	β coefficient: number of weekly mood stabilisers	p	Lower limit	Upper limit	β coefficient: number of weekly mood stabilisers	p	Lower limit	Upper limit
Carbamazepine	-6.3	0.041	-12.2	-0.4	-6.2	0.025	-11.4	-0.9
Lamotrigine	-17.5	0.032	-33.1	-1.9	-14.5	0.046	-28.5	-0.6
Lithium	2.9	0.673	-10.5	16.3	-0.1	0.981	-11.9	11.6
Valproate	-15.4	0.140	-35.5	4.8	-17.5	0.058	-35.2	0.2

Variable	June 2020		95% CI		July 2020		95% CI	
Number of patients enrolled in mental health services	β coefficient: number of patients	p	Lower limit	Upper limit	β coefficient: number of patients	p	Lower limit	Upper limit
Total	-916.2	0.001	-1425.7	-406.8	-688.4	0.005	-1143.9	-232.9
Female	-461.1	0.004	-763.1	-159.2	-394.6	0.006	-664.7	-124.5
Male	-520.8	<0.001	-731.2	-310.5	-364.0	<0.001	-551.8	-176.2
Under 18 years	485.5	0.011	122.9	848.1	547.1	0.002	222.8	871.5
18 to 64 years	-967.0	<0.001	-1163.3	-770.6	-832.7	<0.001	-1007.8	-657.6
65 years and older	-495.4	<0.001	-536.8	-454.0	-460.8	<0.001	-498.0	-423.6
Number of contacts with mental health professionals	β coefficient: number of weekly contacts	p	Lower limit	Upper limit	β coefficient: number of weekly contacts	p	Lower limit	Upper limit
In person	-4902.6	<0.001	-5994.2	-3810.9	-4421.6	<0.001	-5396.5	-3446.7
Remote	5190.0	<0.001	4890.3	5489.7	4914.0	<0.001	4676.1	5151.8
Did not attend (DNA)	-222.4	<0.001	-417.1	-27.6	-139.1	0.118	-310.9	32.8
Number of antipsychotic mentions	β coefficient: number of weekly antipsychotics	p	Lower limit	Upper limit	β coefficient: number of weekly antipsychotics	p	Lower limit	Upper limit
Any antipsychotic	104.8	0.189	-49.5	259.0	122.4	0.087	-15.1	259.9
Any depot	21.9	0.412	-30.0	73.7	32.5	0.177	-14.0	79.1
Any oral	81.8	0.208	-43.9	207.6	92.4	0.112	-19.8	204.7
Number of individual antipsychotic mentions	β coefficient: number of weekly antipsychotics	p	Lower limit	Upper limit	β coefficient: number of weekly antipsychotics	p	Lower limit	Upper limit
Amisulpiride	0.7	0.912	-11.9	13.3	-4.3	0.455	-15.5	6.9
Aripiprazole	31.4	0.130	-8.7	71.4	38.2	0.041	2.4	74.0
Clozapine	18.2	0.342	-19.0	55.4	10.9	0.507	-21.0	42.8
Haloperidol	2.0	0.596	-5.4	9.5	-3.2	0.352	-9.8	3.4
Lurasidone	1.2	0.745	-6.0	8.5	4.0	0.238	-2.6	10.5
Olanzapine	13.5	0.513	-26.7	53.7	13.7	0.457	-22.1	49.5
Quetiapine	25.5	0.025	3.8	47.2	17.0	0.103	-3.1	37.1
Risperidone	4.8	0.688	-18.4	28.0	3.8	0.728	-17.4	25.0
Sulpiride	0.9	0.511	-1.8	3.6	-1.1	0.360	-3.6	1.3
Zuclopenthixol	1.2	0.467	-2.0	4.3	2.6	0.080	-0.3	5.4
Number of antipsychotic depot mentions	β coefficient: number of weekly depots	p	Lower limit	Upper limit	β coefficient: number of weekly depots	p	Lower limit	Upper limit
Aripiprazole depot	26.4	0.003	9.6	43.1	32.8	<0.001	17.6	47.9
Paliperidone depot	7.3	0.618	-21.3	35.9	12.8	0.326	-12.5	38.1
Haloperidol depot	-5.1	0.291	-14.5	4.3	-3.6	0.405	-12.0	4.8
Risperidone depot	-13.6	<0.001	-20.1	-7.1	3.8	0.728	-17.4	25.0
Zuclopenthixol depot	0.3	0.957	-9.4	10.0	-2.6	0.565	-11.5	6.3
Number of mood stabiliser mentions	β coefficient: number of weekly mood stabilisers	p	Lower limit	Upper limit	β coefficient: number of weekly mood stabilisers	p	Lower limit	Upper limit
Any mood stabiliser	-8.3	0.686	-48.4	31.8	-3.9	0.833	-39.7	32.0
Number of individual mood stabiliser mentions	β coefficient: number of weekly mood stabilisers	p	Lower limit	Upper limit	β coefficient: number of weekly mood stabilisers	p	Lower limit	Upper limit
Carbamazepine	-8.3	0.007	-14.1	-2.5	-3.4	0.211	-8.6	1.9
Lamotrigine	-7.0	0.385	-22.6	8.6	-5.1	0.476	-19.1	8.9
Lithium	5.4	0.426	-7.8	18.6	9.5	0.122	-2.3	21.2
Valproate	-6.4	0.525	-25.9	13.1	-8.1	0.368	-25.5	9.4

Variable	August 2020		95% CI		September 2020		95% CI	
Number of patients enrolled in mental health services	β coefficient: number of patients	p	Lower limit	Upper limit	β coefficient: number of patients	p	Lower limit	Upper limit
Total	-778.7	0.004	-1288.3	-269.1	-901.3	0.004	-1489.5	-313.0
Female	-398.2	0.013	-700.9	-95.5	-457.4	0.013	-806.8	-108.1
Male	-401.3	<0.001	-612.0	-190.7	-464.1	<0.001	-707.2	-220.9
Under 18 years	572.1	0.003	211.6	932.6	480.8	0.028	64.5	897.2
18 to 64 years	-951.2	<0.001	-1149.5	-752.9	-1020.1	<0.001	-1248.2	-792.1
65 years and older	-403.5	<0.001	-444.6	-362.4	-366.3	<0.001	-413.7	-318.9
Number of contacts with mental health professionals	β coefficient: number of weekly contacts	p	Lower limit	Upper limit	β coefficient: number of weekly contacts	p	Lower limit	Upper limit
In person	-4388.9	<0.001	-5478.9	-3299.0	-4401.4	<0.001	-5661.8	-3141.1
Remote	5571.0	<0.001	5308.0	5834.1	5008.2	<0.001	4672.4	5344.0
Did not attend (DNA)	-173.5	0.082	-365.0	18.1	-278.9	0.018	-502.5	-55.3
Number of antipsychotic mentions	β coefficient: number of weekly antipsychotics	p	Lower limit	Upper limit	β coefficient: number of weekly antipsychotics	p	Lower limit	Upper limit
Any antipsychotic	102.7	0.325	-99.8	305.2	3.0	0.980	-231.0	237.0
Any depot	59.3	0.110	12.3	130.9	17.2	0.683	-65.2	99.6
Any oral	51.9	0.522	-106.1	210.0	-17.3	0.854	-200.8	166.2
Number of individual antipsychotic mentions	β coefficient: number of weekly antipsychotics	p	Lower limit	Upper limit	β coefficient: number of weekly antipsychotics	p	Lower limit	Upper limit
Amisulpiride	-3.5	0.603	-16.8	9.7	-15.9	0.047	-31.2	-0.6
Aripiprazole	18.1	0.495	-33.6	69.8	-5.5	0.857	-65.3	54.2
Clozapine	31.4	0.100	-5.3	68.1	5.1	0.813	-37.2	47.5
Haloperidol	-7.6	0.087	-16.2	1.0	-1.4	0.785	-11.8	8.9
Lurasidone	2.9	0.472	-5.0	10.8	-2.6	0.581	-11.8	6.6
Olanzapine	-0.6	0.983	-53.0	51.9	-10.3	0.742	-71.6	50.9
Quetiapine	8.3	0.551	-18.7	35.2	4.9	0.758	-26.2	36.1
Risperidone	-20.5	0.183	-50.3	9.3	-23.5	0.190	-58.2	11.2
Sulpiride	-1.7	0.230	-4.4	1.0	-1.7	0.291	-4.8	1.4
Zuclopenthixol	3.3	0.052	0.0	6.6	-0.4	0.824	-4.1	3.3
Number of antipsychotic depot mentions	β coefficient: number of weekly depots	p	Lower limit	Upper limit	β coefficient: number of weekly depots	p	Lower limit	Upper limit
Aripiprazole depot	41.5	<0.001	23.6	59.5	37.6	0.001	16.7	58.6
Paliperidone depot	18.4	0.242	-12.1	49.0	-8.0	0.655	-42.9	26.9
Haloperidol depot	-5.3	0.393	-17.3	6.7	-3.0	0.671	-16.9	10.8
Risperidone depot	-9.7	0.008	-16.6	-2.8	-9.8	0.018	-17.7	-1.9
Zuclopenthixol depot	12.4	0.122	-3.1	27.8	4.9	0.596	-13.1	22.8
Number of mood stabiliser mentions	β coefficient: number of weekly mood stabilisers	p	Lower limit	Upper limit	β coefficient: number of weekly mood stabilisers	p	Lower limit	Upper limit
Any mood stabiliser	-0.3	0.989	-48.8	48.1	-26.6	0.361	-83.2	30.0
Number of individual mood stabiliser mentions	β coefficient: number of weekly mood stabilisers	p	Lower limit	Upper limit	β coefficient: number of weekly mood stabilisers	p	Lower limit	Upper limit
Carbamazepine	-8.3	0.009	-14.3	-2.3	-9.3	0.012	-16.3	-2.3
Lamotrigine	-9.2	0.308	-26.7	8.3	-20.8	0.049	-41.0	-0.5
Lithium	6.6	0.409	-9.0	22.2	8.6	0.363	-9.8	27.0
Valproate	12.2	0.293	-10.3	34.8	-5.7	0.673	-32.1	20.7

< 0.001	<0.01	<0.05

For peer review only

Impact of the COVID-19 pandemic on remote mental healthcare and prescribing in psychiatry: an electronic health record study

Supplementary Material

Table of Contents

Interpretation of the interactive dashboard 1

Supplementary Figure 1. Number of patients registered with SLaM services each week by gender 2

Supplementary Figure 2. Number of patients newly registered with SLaM services each week...................... 3

Supplementary Figure 3. Number of remote consultations by video call vs telephone each month 3

Supplementary Figure 4. Remote consultations by video-call vs telephone as percentages of the total 4

Supplementary Figure 5. Total number of clinical events each week by gender.. 4

Supplementary Figure 6. Number of in-person, remote and non-attended clinical events each week by gender 5

Supplementary Figure 7. Number of antipsychotics recorded each week 5

Supplementary Figure 8. Number of antipsychotics recorded per week in structured fields vs free text 6

Supplementary Figure 9. Number of antipsychotics recorded each week by age group 6

Supplementary Figure 10. Number of antipsychotics recorded each week by gender 7

Supplementary Figure 11: Number of mood stabilisers recorded each week 7

Supplementary Figure 12. Number of mood stabilisers recorded each week by gender................................ 8

Supplementary Figure 13. Number of mood stabilisers recorded each week by age group 8

Interpretation of the interactive dashboard

The data visualisation dashboard at http://rpatel.co.uk/TelepsychiatryDashboard has a number of interactive capabilities:

1. Click and drag along the axes to scroll to different values
2. To zoom in on a time period, click and drag horizontally in the chart space to highlight the time period of interest
3. To zoom in on a data series, click and drag vertically in the chart space to highlight the data series of interest
4. Double click to exit zoom mode and revert to default
5. Click on a legend item to hide that category of data from the chart temporarily

For peer review only - http://bmjopen.bmj.com/site/about/guidelines.xhtml

More information can be found at https://exploratory.io/note/2ac8ae888097/0937480844541865.

Supplementary Figure 1. Number of patients registered with SLaM services each week by gender

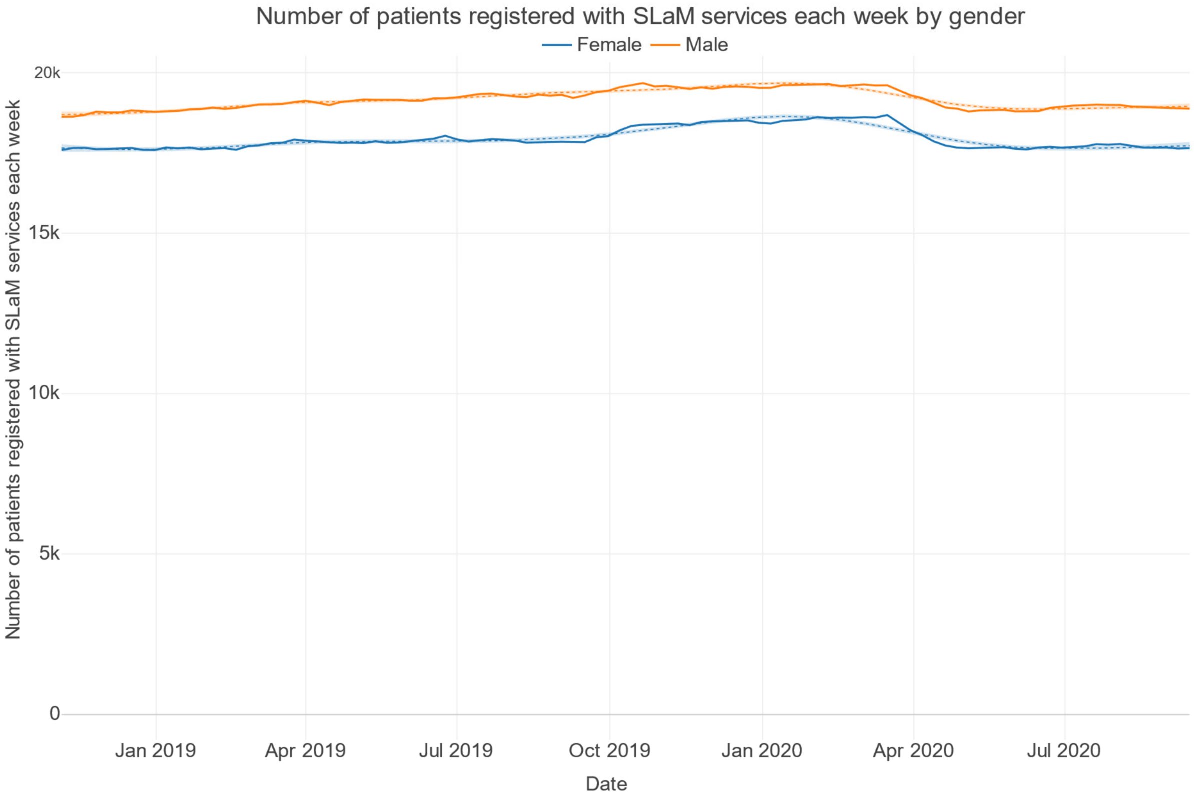

Supplementary Figure 2. Number of patients newly registered with SLaM services each week

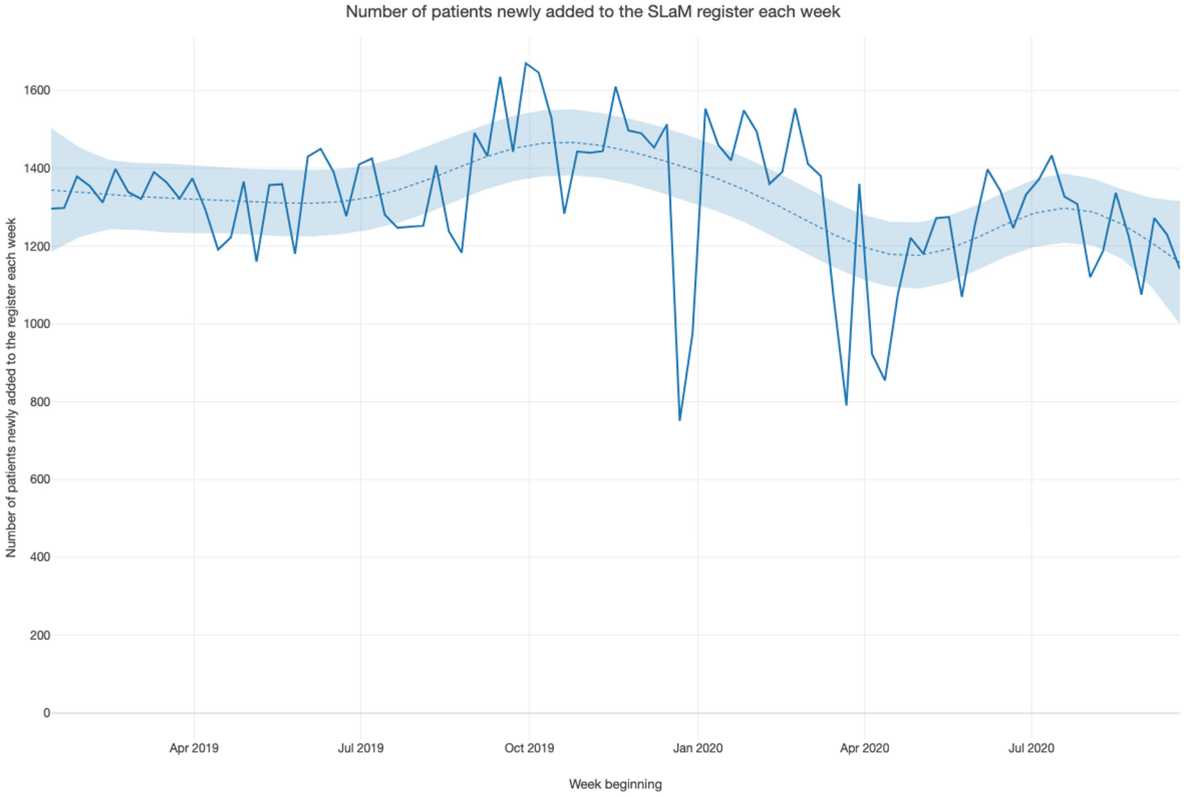

Supplementary Figure 3. Number of remote consultations by video call vs telephone each month

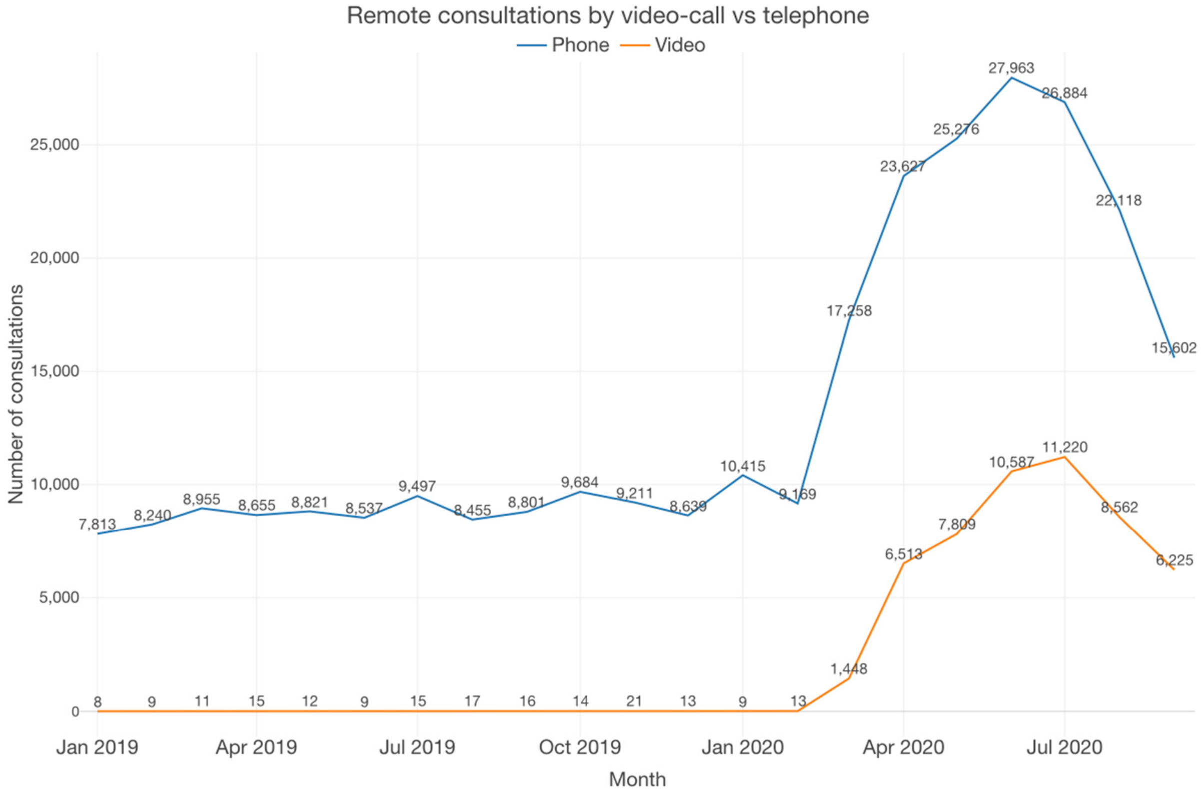

Supplementary Figure 4. Percentage of remote consultations by video-call vs telephone

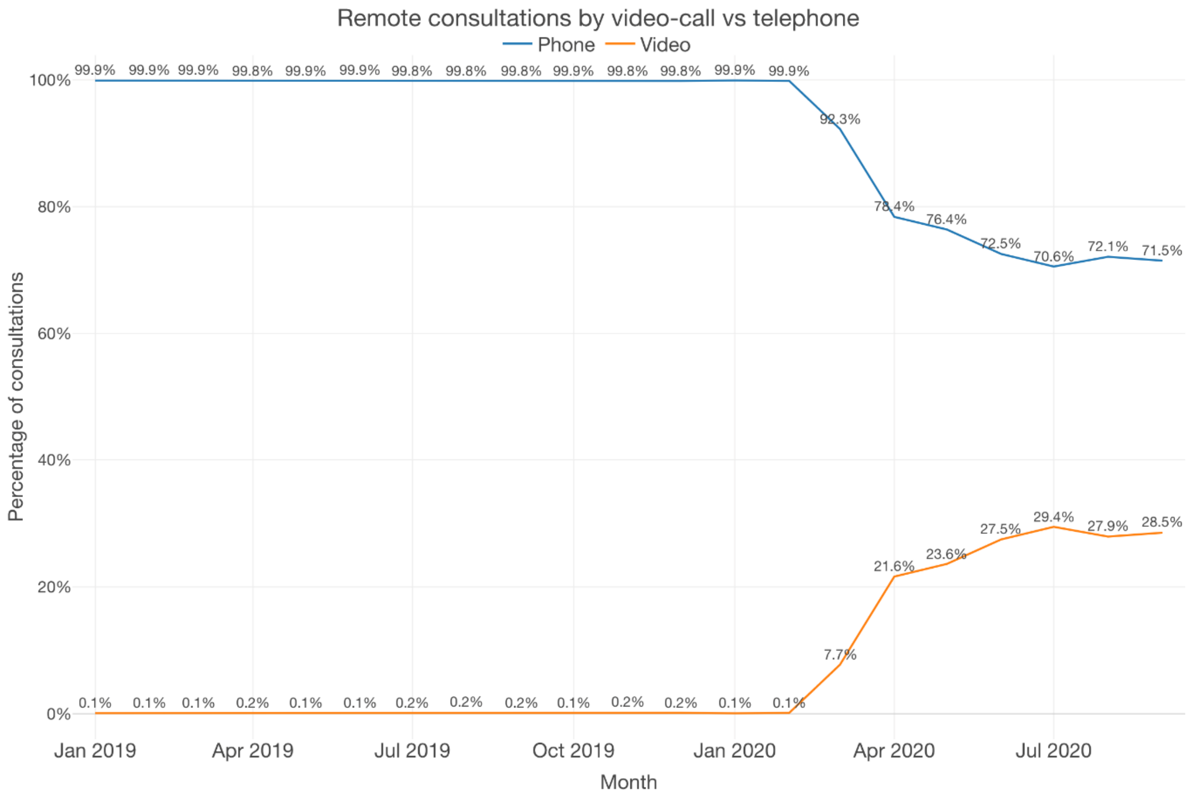

Supplementary Figure 5. Total number of clinical events each week by gender

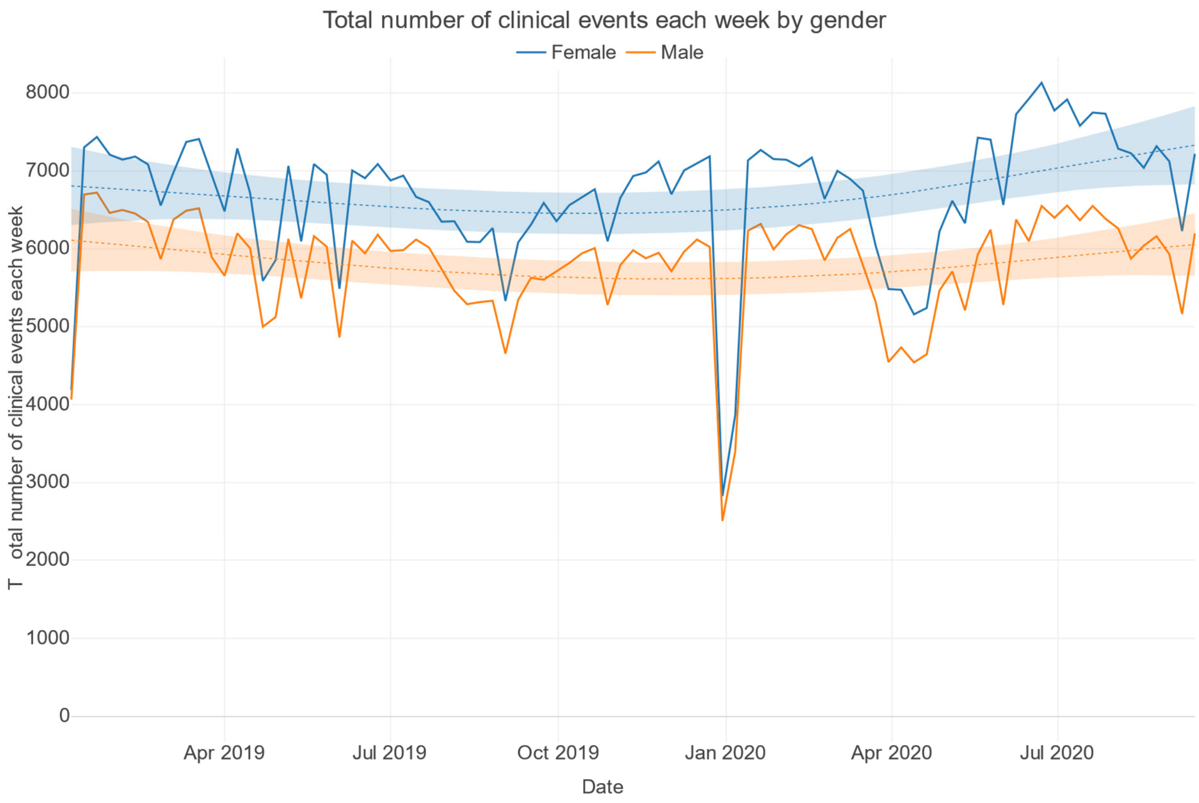

Supplementary Figure 6. Number of in-person, remote and non-attended clinical events each week by gender

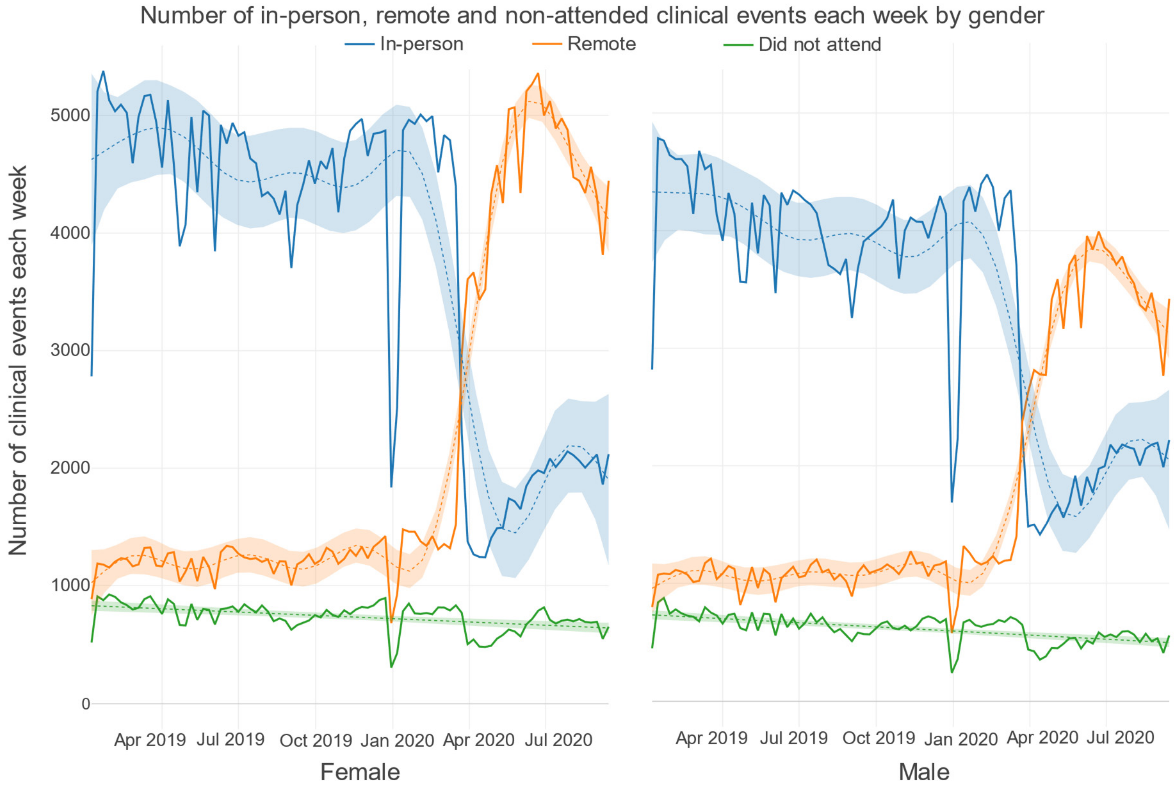

Supplementary Figure 7. Number of antipsychotics recorded each week

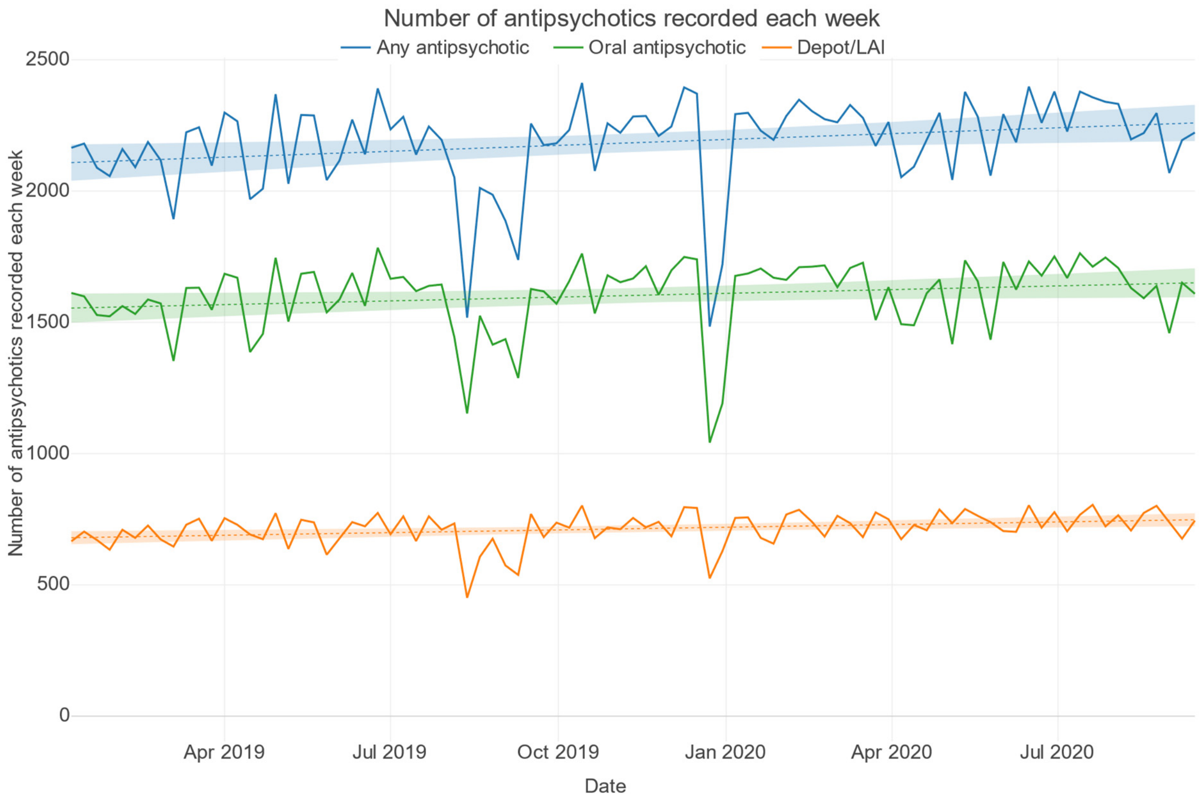

Supplementary Figure 8. Number of antipsychotics recorded per week in structured fields vs free text

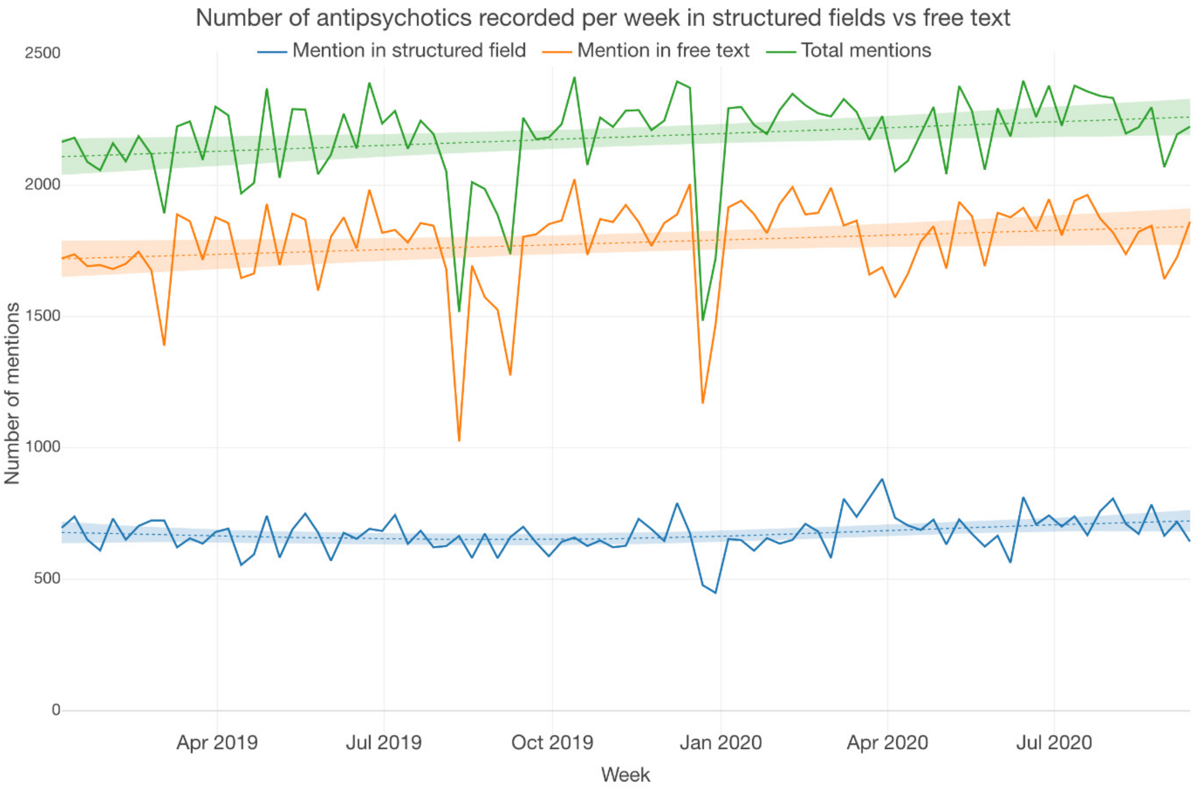

Supplementary Figure 9. Number of antipsychotics recorded each week by age group

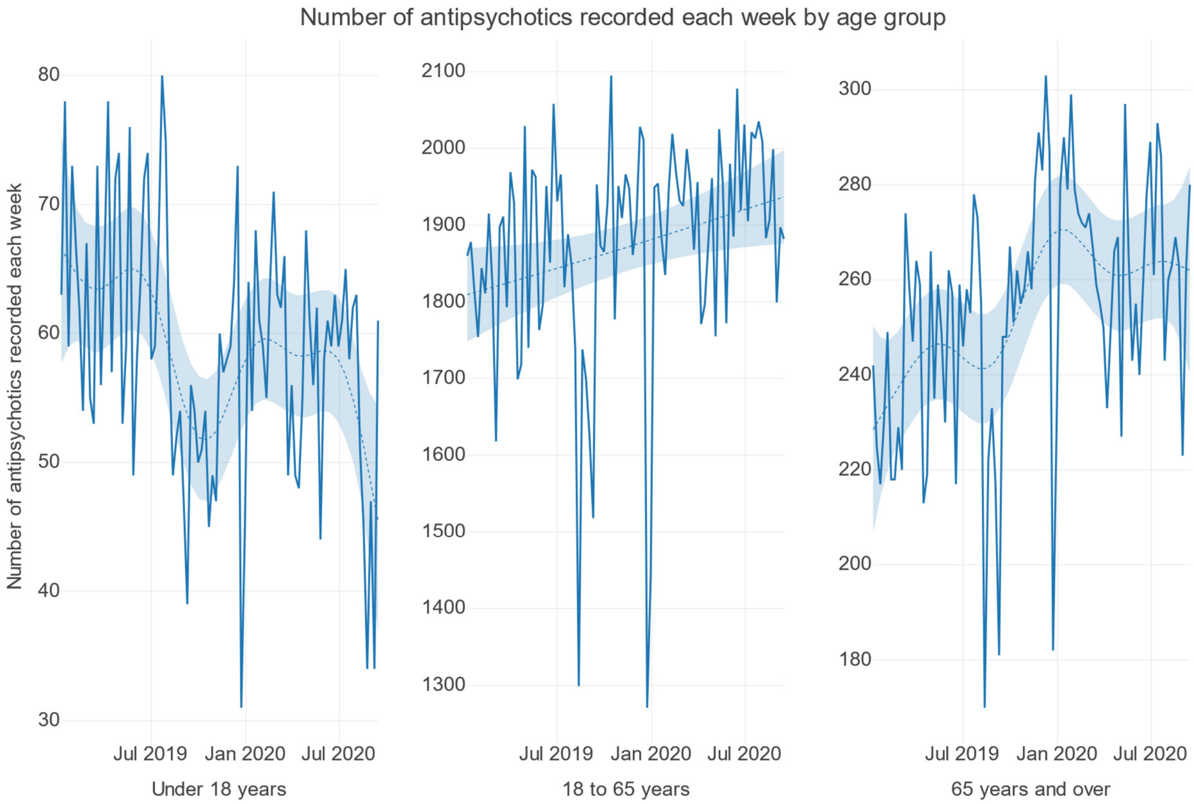

Note: The y-axes presented here do not all start at zero and are not standardised between charts.

Supplementary Figure 10. Number of antipsychotics recorded each week by gender

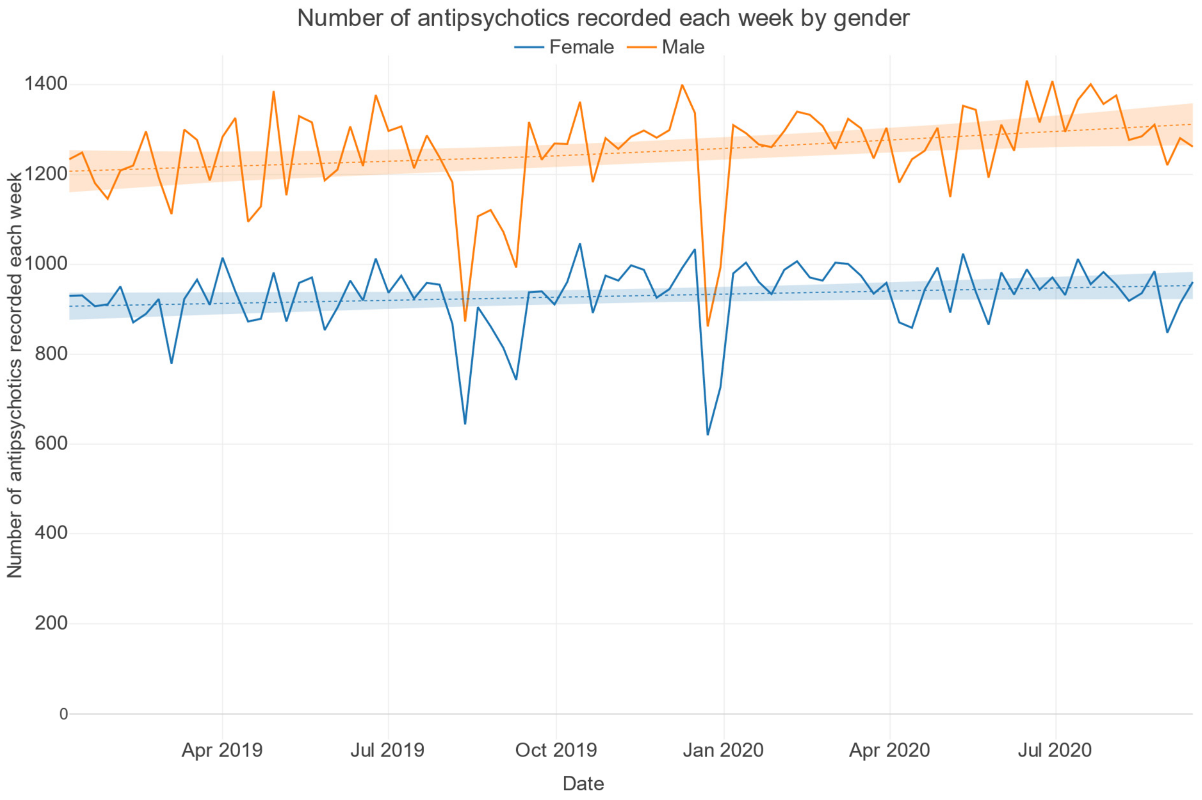

Supplementary Figure 11: Number of mood stabilisers recorded each week

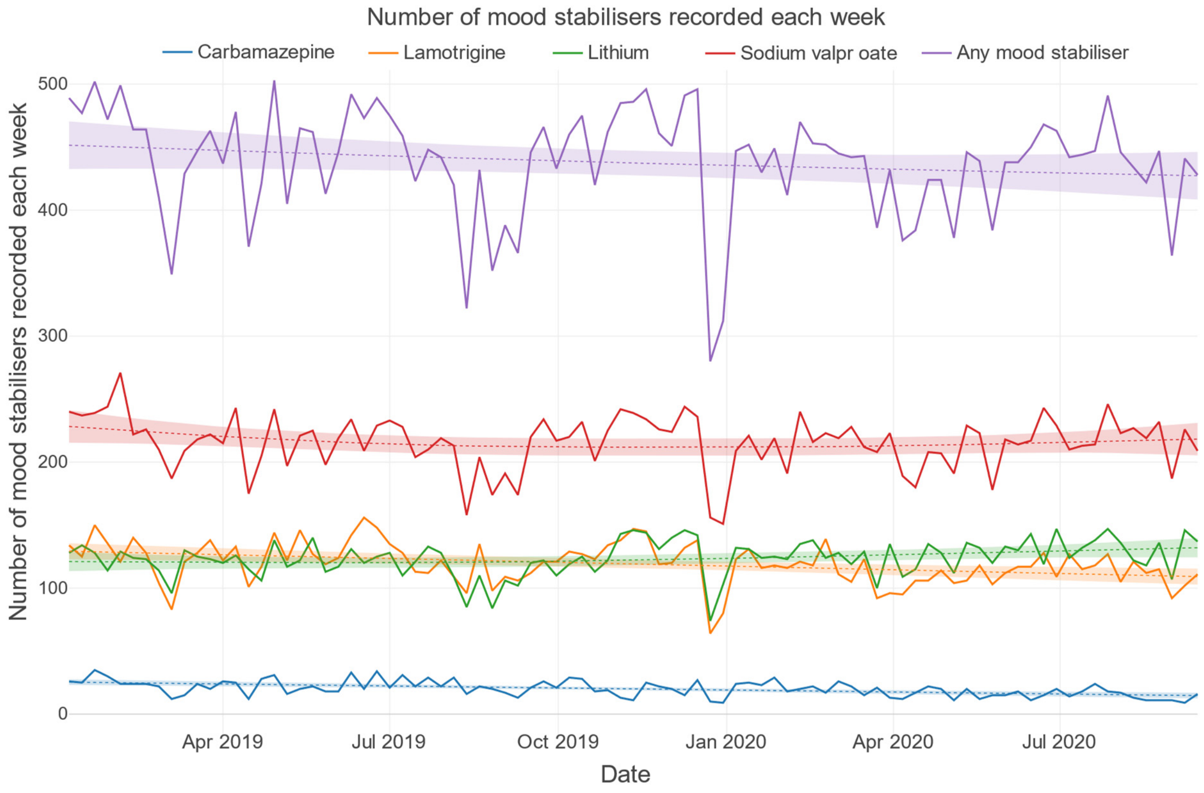

Supplementary Figure 12. Number of mood stabilisers recorded each week by gender

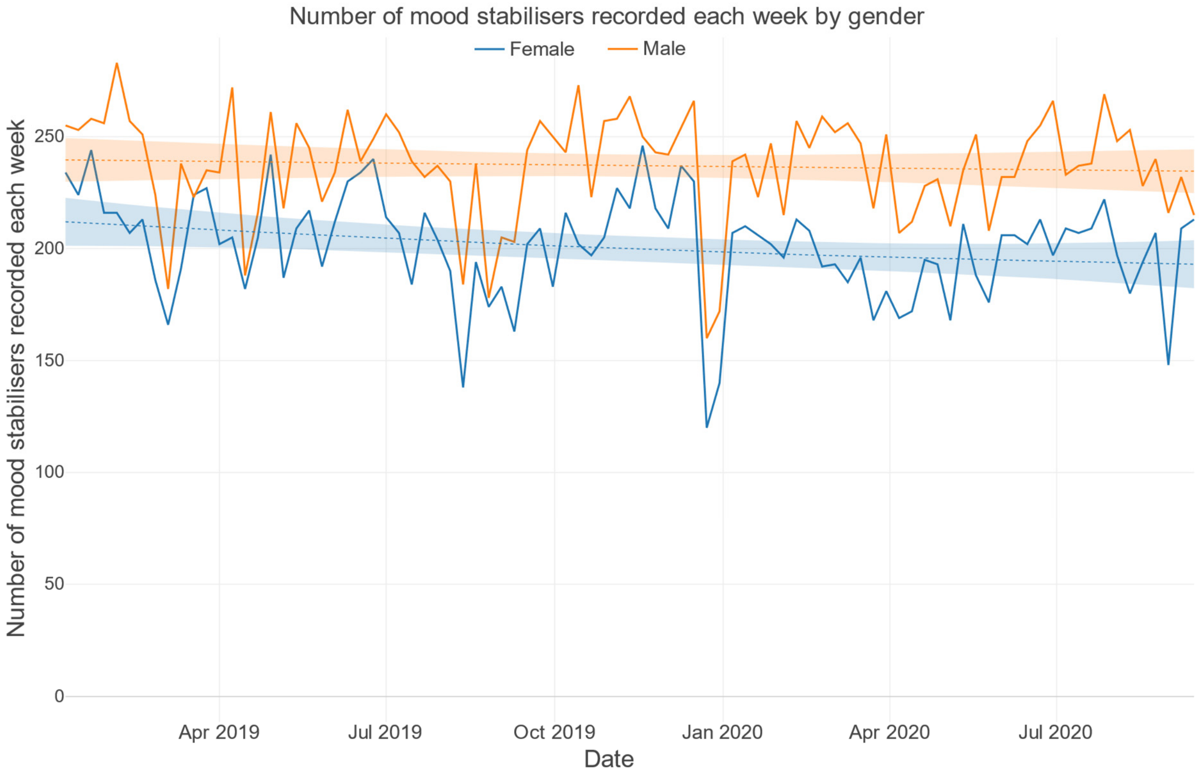

Supplementary Figure 13. Number of mood stabilisers recorded each week by age group

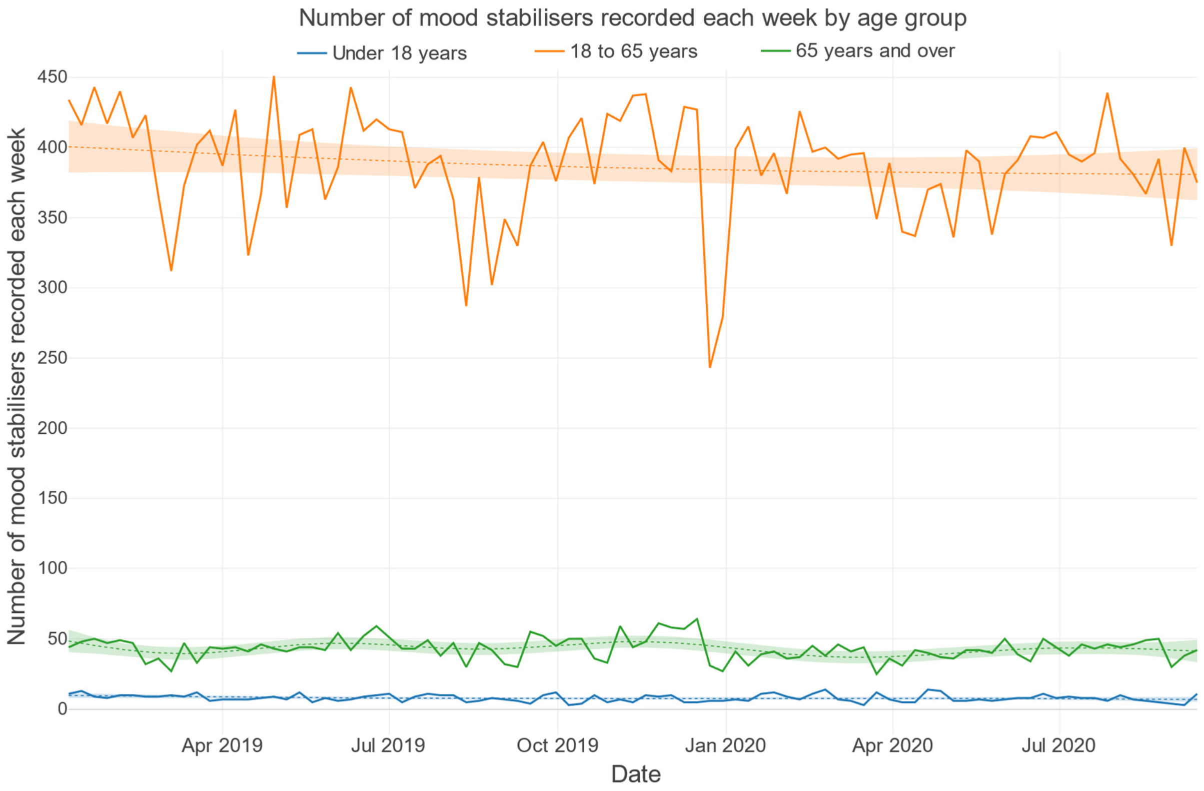

For peer review only - http://bmjopen.bmj.com/site/about/guidelines.xhtml

The RECORD statement – checklist of items, extended from the STROBE statement, that should be reported in observational studies using routinely collected health data.

	Item No.	STROBE items	Location in manuscript where items are reported	RECORD items	Location in manuscript where items are reported
**Title and abstract**					
	1	(a) Indicate the study's design with a commonly used term in the title or the abstract (b) Provide in the abstract an informative and balanced summary of what was done and what was found	Pages 1 and 3	RECORD 1.1: The type of data used should be specified in the title or abstract. When possible, the name of the databases used should be included.<br><br>RECORD 1.2: If applicable, the geographic region and timeframe within which the study took place should be reported in the title or abstract.<br><br>RECORD 1.3: If linkage between databases was conducted for the study, this should be clearly stated in the title or abstract.	Page 3<br><br>Page 3<br><br>Not applicable
**Introduction**					
Background rationale	2	Explain the scientific background and rationale for the investigation being reported	Page 5		
Objectives	3	State specific objectives, including any prespecified hypotheses	Page 5		
**Methods**					
Study Design	4	Present key elements of study design early in the paper	Page 6		
Setting	5	Describe the setting, locations, and relevant dates, including periods of recruitment, exposure, follow-up, and data collection	Page 6		

For peer review only

Participants	6	*(a) Cohort study* - Give the eligibility criteria, and the sources and methods of selection of participants. Describe methods of follow-up<br>*Case-control study* - Give the eligibility criteria, and the sources and methods of case ascertainment and control selection. Give the rationale for the choice of cases and controls<br>*Cross-sectional study* - Give the eligibility criteria, and the sources and methods of selection of participants<br><br>*(b) Cohort study* - For matched studies, give matching criteria and number of exposed and unexposed<br>*Case-control study* - For matched studies, give matching criteria and the number of controls per case	Page 6 Participants and setting / Data extraction<br><br>Not applicable<br><br>Not applicable	RECORD 6.1: The methods of study population selection (such as codes or algorithms used to identify subjects) should be listed in detail. If this is not possible, an explanation should be provided.<br><br>RECORD 6.2: Any validation studies of the codes or algorithms used to select the population should be referenced. If validation was conducted for this study and not published elsewhere, detailed methods and results should be provided.<br><br>RECORD 6.3: If the study involved linkage of databases, consider use of a flow diagram or other graphical display to demonstrate the data linkage process, including the number of individuals with linked data at each stage.	Page 6 Data extraction / Defined variables<br><br>Page 6 Data extraction<br><br>Not applicable
Variables	7	Clearly define all outcomes, exposures, predictors, potential confounders, and effect modifiers. Give diagnostic criteria, if applicable.	Pages 6 to 7	RECORD 7.1: A complete list of codes and algorithms used to classify exposures, outcomes, confounders, and effect modifiers should be provided. If these cannot be reported, an explanation should be provided.	Pages 6 to 7
Data sources/ measurement	8	For each variable of interest, give sources of data and details of methods of assessment (measurement).<br>Describe comparability of assessment methods if there is more than one group	Pages 6 to 7		

For peer review only

Bias	9	Describe any efforts to address potential sources of bias	Pages 6 to 7		
Study size	10	Explain how the study size was arrived at	Page 6		
Quantitative variables	11	Explain how quantitative variables were handled in the analyses. If applicable, describe which groupings were chosen, and why	Page 7		
Statistical methods	12	(a) Describe all statistical methods, including those used to control for confounding<br>(b) Describe any methods used to examine subgroups and interactions<br>(c) Explain how missing data were addressed<br>(d) *Cohort study* - If applicable, explain how loss to follow-up was addressed<br>*Case-control study* - If applicable, explain how matching of cases and controls was addressed<br>*Cross-sectional study* - If applicable, describe analytical methods taking account of sampling strategy<br>(e) Describe any sensitivity analyses	Page 7<br>Page 7<br>Page 7<br>Not applicable<br>Not applicable		
Data access and cleaning methods		..		RECORD 12.1: Authors should describe the extent to which the investigators had access to the database population used to create the study population.	Page 6

For peer review only

				RECORD 12.2: Authors should provide information on the data cleaning methods used in the study.	Page 7
Linkage		..		RECORD 12.3: State whether the study included person-level, institutional-level, or other data linkage across two or more databases. The methods of linkage and methods of linkage quality evaluation should be provided.	Not applicable
**Results**					
Participants	13	(a) Report the numbers of individuals at each stage of the study (*e.g.*, numbers potentially eligible, examined for eligibility, confirmed eligible, included in the study, completing follow-up, and analysed)<br>(b) Give reasons for non-participation at each stage.<br>(c) Consider use of a flow diagram	Page 8/Supplementary Table 1<br><br>Not applicable<br><br>Not applicable	RECORD 13.1: Describe in detail the selection of the persons included in the study (*i.e.,* study population selection) including filtering based on data quality, data availability and linkage. The selection of included persons can be described in the text and/or by means of the study flow diagram.	Not applicable
Descriptive data	14	(a) Give characteristics of study participants (*e.g.*, demographic, clinical, social) and information on exposures and potential confounders<br>(b) Indicate the number of participants with missing data for each variable of interest<br>(c) *Cohort study* - summarise follow-up time (*e.g.*, average and total amount)	Page 8/Supplementary Table 1<br><br>Not applicable<br><br>Not applicable		
Outcome data	15	*Cohort study* - Report numbers of outcome events or summary measures over time<br>*Case-control study* - Report numbers in each exposure	Pages 8 to 9/Supplementary Table 1		

For peer review only

		category, or summary measures of exposure<br>*Cross-sectional study* - Report numbers of outcome events or summary measures			
Main results	16	(a) Give unadjusted estimates and, if applicable, confounder-adjusted estimates and their precision (e.g., 95% confidence interval). Make clear which confounders were adjusted for and why they were included<br>(b) Report category boundaries when continuous variables were categorized<br>(c) If relevant, consider translating estimates of relative risk into absolute risk for a meaningful time period	Supplementary Table 1<br>Page 6<br>Not applicable		
Other analyses	17	Report other analyses done—e.g., analyses of subgroups and interactions, and sensitivity analyses	Not applicable		
**Discussion**					
Key results	18	Summarise key results with reference to study objectives	Page 10		
Limitations	19	Discuss limitations of the study, taking into account sources of potential bias or imprecision. Discuss both direction and magnitude of any potential bias	Pages 11 to 12	RECORD 19.1: Discuss the implications of using data that were not created or collected to answer the specific research question(s). Include discussion of misclassification bias, unmeasured confounding, missing data, and changing eligibility over time, as they pertain to the study being reported.	Pages 11 to 12
Interpretation	20	Give a cautious overall interpretation of results considering objectives,	Pages 11 to 12		

For peer review only - http://bmjopen.bmj.com/site/about/guidelines.xhtml

		limitations, multiplicity of analyses, results from similar studies, and other relevant evidence			
Generalisability	21	Discuss the generalisability (external validity) of the study results	Pages 11 to 12		
**Other Information**					
Funding	22	Give the source of funding and the role of the funders for the present study and, if applicable, for the original study on which the present article is based	Page 2		
Accessibility of protocol, raw data, and programming code		..		RECORD 22.1: Authors should provide information on how to access any supplemental information such as the study protocol, raw data, or programming code.	Page 2

*Reference: Benchimol EI, Smeeth L, Guttmann A, Harron K, Moher D, Petersen I, Sørensen HT, von Elm E, Langan SM, the RECORD Working Committee. The REporting of studies Conducted using Observational Routinely-collected health Data (RECORD) Statement. *PLoS Medicine* 2015; in press.

*Checklist is protected under Creative Commons Attribution (CC BY) license.

For peer review only
